# Uncertain dynamic response of mid-latitude winter precipitation

**DOI:** 10.1038/s41586-026-10474-y

**Published:** 2026-04-29

**Authors:** Lei Gu, Dominik L. Schumacher, Sebastian Sippel, Erich M. Fischer, Istvan Dunkl, Robin Noyelle, Jitendra Singh, Lorenzo Pierini, Reto Knutti

**Affiliations:** 1 Institute for Atmospheric and Climate Science, ETH Zurich, Zurich, Switzerland; 2 Atmospheric, Oceanic, and Planetary Physics, University of Oxford, Oxford, UK; 3 Leipzig Institute for Meteorology, Leipzig University, Leipzig, Germany

**Keywords:** Atmospheric science, Hydrology, Climate change, Hydrology

## Abstract

Understanding changes in precipitation is crucial for society and ecosystems^1,2^. Studies have documented the respective contributions of anthropogenic forcing and internal variability to precipitation trends^3,4^, yet discrepancies persist between observed and simulated patterns. In Northern Hemisphere winter, these mismatches are often attributed to unforced internal variability that dominates observed trends^5^. However, growing evidence also indicates that climate models underestimate the total response of precipitation to human forcings^6–8^. Here we show that the thermodynamic contribution is broadly reproduced by climate models, whereas the dynamic contribution can diverge more substantially. Our approach disentangles the anthropogenic forced thermodynamic and dynamic components from internal variability in winter precipitation trends (1950–2022) to investigate their contribution to the trend discrepancies. In the Mediterranean, the forced dynamic signal from model simulations explains only about 10% of the observed dynamic trend, making detection challenging. Under continued anthropogenic emissions, the projected circulation response intensifies and more closely resembles observed trend patterns. Although internal variability in the observed record may contribute to this similarity, the results indicate an uncertain yet potentially emerging role of dynamic response in shaping regional winter precipitation trends. A reliable representation of the forced large-scale circulation response in climate models remains key for increasing confidence in regional precipitation projections.

## Abstract (web summary)

Separating the anthropogenic forced thermodynamic and dynamic components from internal variability in winter precipitation trends in the mid-latitudes shows that thermodynamic effects agree with models and observations but the role of dynamic response remains uncertain.

## Main

Terrestrial precipitation is vital to ecosystems, agriculture and water and energy security^9,10^. Yet, the drivers of multidecadal precipitation trends at the regional scale remain unclear^11^. In the Northern Hemisphere, winter precipitation is largely governed by the large-scale atmospheric circulation, in particular the Hadley circulation and the mid-latitude westerly jet^12,13^. The North Atlantic Oscillation (NAO) represents a dominant mode of jet variability: in its positive phase, a strengthened jet brings wet conditions to eastern North America (ENA) and northern Europe (NEU) and dry conditions to southern Europe (SEU)^7,14,15^. Also, precipitation is modulated by coupled ocean–atmosphere variability, notably El Niño–Southern Oscillation^16,17^.

However, anthropogenic forcings are increasingly affecting atmospheric circulation patterns, resulting in regionally varied climate responses^18^. For instance, the upward trend in the NAO, possibly responding to human emissions, underscores the potential for forced dynamic shifts in precipitation^19,20^. Meanwhile, extensive evidence shows that human forcing drives thermodynamic changes in precipitation through altered tropospheric temperatures and humidity^21–23^, modified land–ocean thermal contrasts^24^, lapse rate changes^25^ and land–atmosphere feedbacks^26,27^. Together with circulation changes, these processes shape observed trends in terrestrial precipitation^28^.

Despite these insights, discrepancies persist between observed and simulated precipitation trends, particularly for the winter precipitation in the Northern Hemisphere^8^. Simulated trends involve substantial uncertainties^29^, partly because of inherent unforced internal variability, and observed trends often fall near the bounds of model distributions^6^. One possible explanation is that rare internal variability is underrepresented in climate model ensembles. For example, the winter precipitation drying over the Mediterranean since 1950 has been found to be consistent with internal variability, yet the observed trend is barely consistent with the range simulated by climate models^5^. Alternatively, recent detection and attribution studies imply that such discrepancies could originate from misrepresentation of regional responses to anthropogenic emissions^7,30,31^. Present climate models do not reproduce the response of the North Atlantic jet stream to external forcing^7,32^. Also, the biased representation of climate change effects on precipitation–temperature–humidity coupling in climate models contributes to the mismatch^33^. These competing interpretations highlight the need to distinguish between internal-variability-driven mismatches and those arising from an underestimation of forced thermodynamic or circulation-related changes.

Here we develop a statistical learning framework combined with targeted climate model experiments using nudged winds, that is, constrained atmospheric circulation. Our goal is to disentangle the thermodynamic and dynamic contributions to winter precipitation trends and to separate the role of anthropogenic forcing from unforced internal variability. The analysis focuses on mid-to-high-latitude land regions in the Northern Hemisphere over the period 1950–2022 (Supplementary Fig. 1). The statistical models are calibrated using an ensemble of pre-industrial simulations from the Coupled Model Intercomparison Project Phase 6 (CMIP6) and then applied to several reanalysis datasets, the 100-member Community Earth System Model v2 Large Ensemble (CESM2-LE)^34^, and CMIP6 outputs under historical and Shared Socioeconomic Pathway (SSP) scenarios (Methods). The CESM2 nudging experiments consist of two experiments (Extended Data Table 1): first, pre-industrial simulations are nudged towards standard fully coupled historical and SSP simulations; second, the atmospheric circulation from both pre-industrial and historical–SSP simulations is nudged towards the European Centre for Medium-Range Weather Forecasts Reanalysis v5 (ERA5). This dual approach improves the reliability of the decomposition of precipitation trends.

## Precipitation trends in boreal winter

**Fig. 1:**
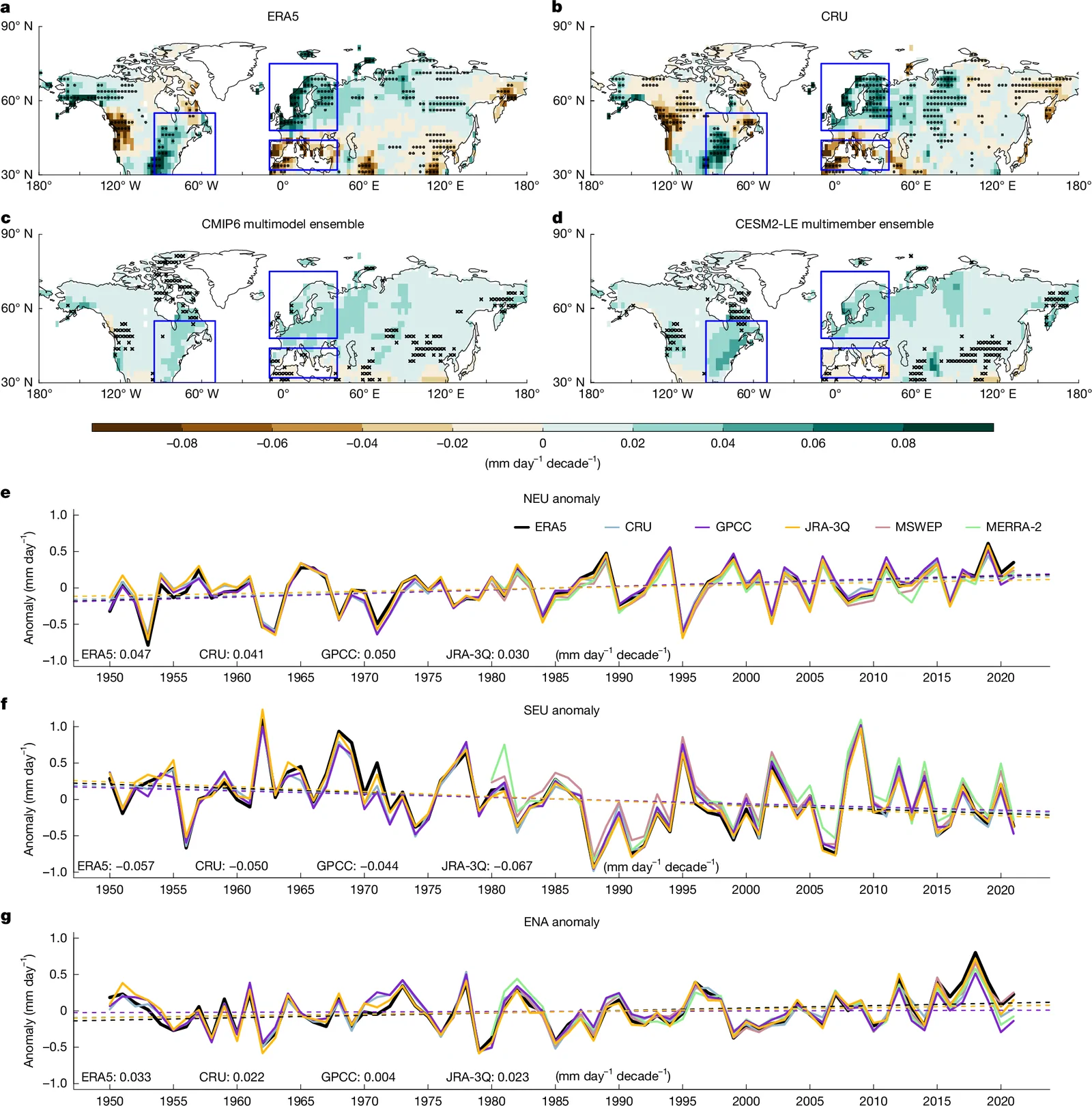
Observed and simulated trends in winter precipitation (1950–2022). **a**,**b**, Winter (DJF) precipitation trends from ERA5 (**a**) and CRU (**b**). Dot stippling in **a**,**b** indicates regions in which the trend is significant (*P* value < 0.05). **c**,**d**, Multimodel ensemble (from CMIP6) average precipitation (**c**) and multimember ensemble (from CESM2-LE) average precipitation (**d**). Cross stippling in **c**,**d** indicates regions in which ERA5 trends lie outside the ensemble range (minimum and maximum values) of CMIP6 or CESM2-LE. The blue boxes represent three regions: ENA (95° W–50° W, 30° N–55° N), NEU (10° W–40° E, 48° N–75° N) and SEU (10° W–40° E, 32° N–44° N). **e**–**g**, Evolution and trends of winter precipitation series over NEU (**e**), SEU (**f**) and ENA (**g**) estimated from ERA5, CRU, GPCC, JRA-3Q, MSWEP and MERRA-2 datasets.

**Extended Data Fig. 1:**
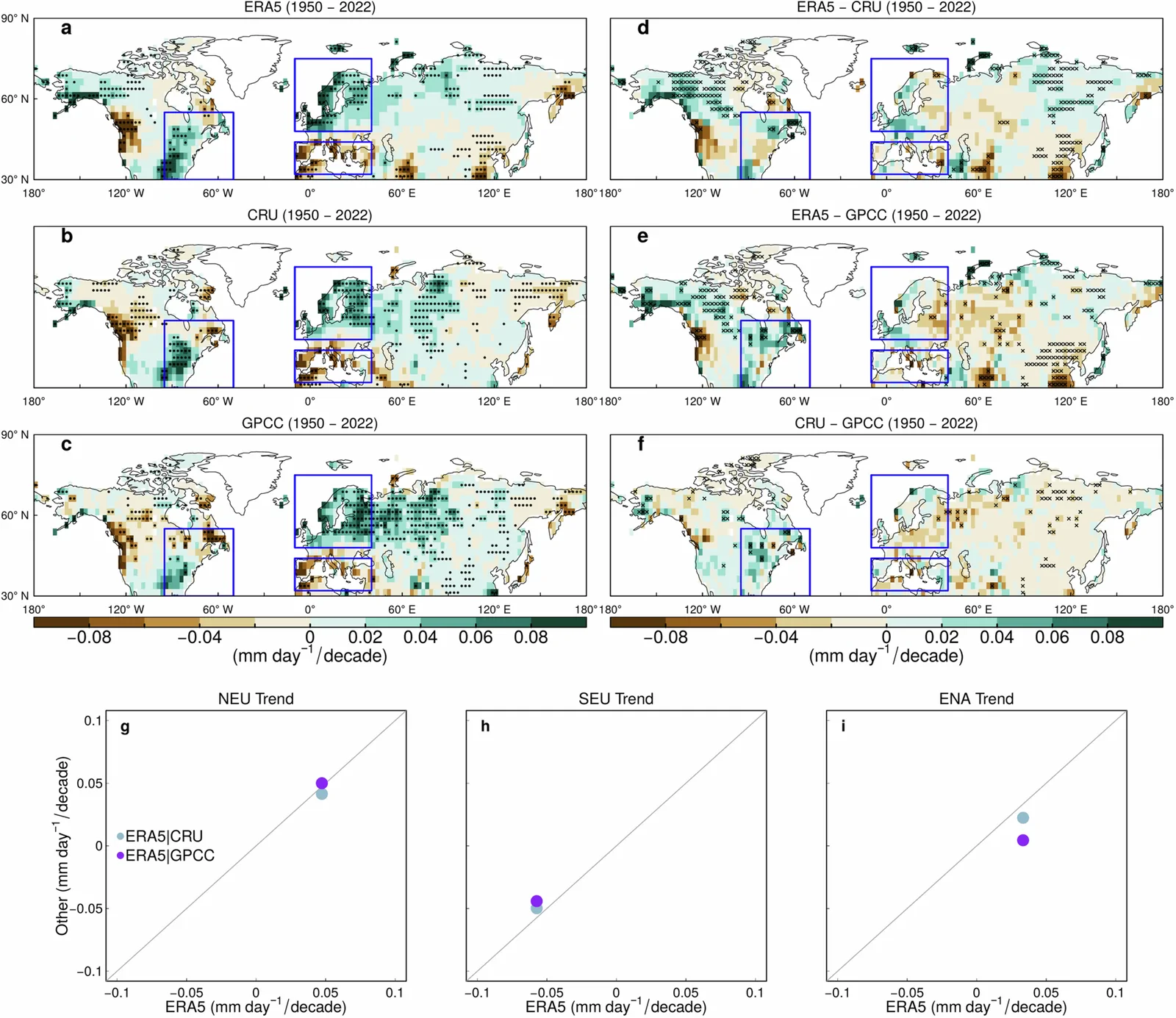
The observation-based winter precipitation trends (1950–2022). **a**–**c**, The observation-based winter precipitation trends from ERA5 (**a**), CRU (**b**) and GPCC (**c**) datasets. Dot stippling indicates regions in which trends are significant at the 0.05 level (*P*-value < 0.05). **d**–**f**, Differences between ERA5 and CRU (**d**), ERA5 and GPCC (**e**) and CRU and GPCC (**f**). Crosses mark the trends estimated by the ERA5 (or CRU) significantly differ from the trends estimated by CRU and GPCC (or GPCC) (two-sided *t*-test, *P*-value < 0.05). **g**–**i**, Scatter plots comparing winter precipitation trends in ERA5 with other observational datasets (CRU and GPCC) over NEU (**g**), SEU (**h**) and ENA (**i**).

We begin by examining precipitation trends during winter in the Northern Hemisphere (December–February, DJF) using data from the ERA5, Climatic Research Unit gridded Time Series version 4 (CRU TS v4) and Global Precipitation Climatology Centre (GPCC) over the period 1950–2022 (Fig. 1). The patterns show considerable spatial heterogeneity. For example, ENA and NEU exhibit significant (*P* value < 0.05) increases in precipitation (0.03 and 0.05 mm day^−1^ decade^−1^), whereas SEU shows a significantly negative trend of −0.06 mm day^−1^ decade^−1^, despite some grid-scale discrepancies among datasets (Extended Data Fig. 1). The upward trends in ENA and NEU are accompanied by increasing precipitation variability^2^, indicating an intensified hydrological cycle in these regions. In other areas of the mid-latitudes, the trends are generally uncertain (Fig. 1, Extended Data Fig. 1 and Supplementary Fig. 2).

Next we quantify trends from 29 CMIP6 models and the 100-member CESM2 ensemble and estimate externally forced responses of winter precipitation^35^ (Fig. 1). The two ensembles capture the trends well, with the reanalysis-based trends mostly falling within the range of the CMIP6/CESM2 ensemble. For example, CESM2 estimates predominantly positive precipitation trends over ENA and NEU (−0.01 to 0.09 and −0.02 to 0.08 mm day^−1^ decade^−1^, respectively) but close to zero or weakly negative trends over SEU (−0.06 to 0.04 mm day^−1^ decade^−1^).

**Extended Data Fig. 2:**
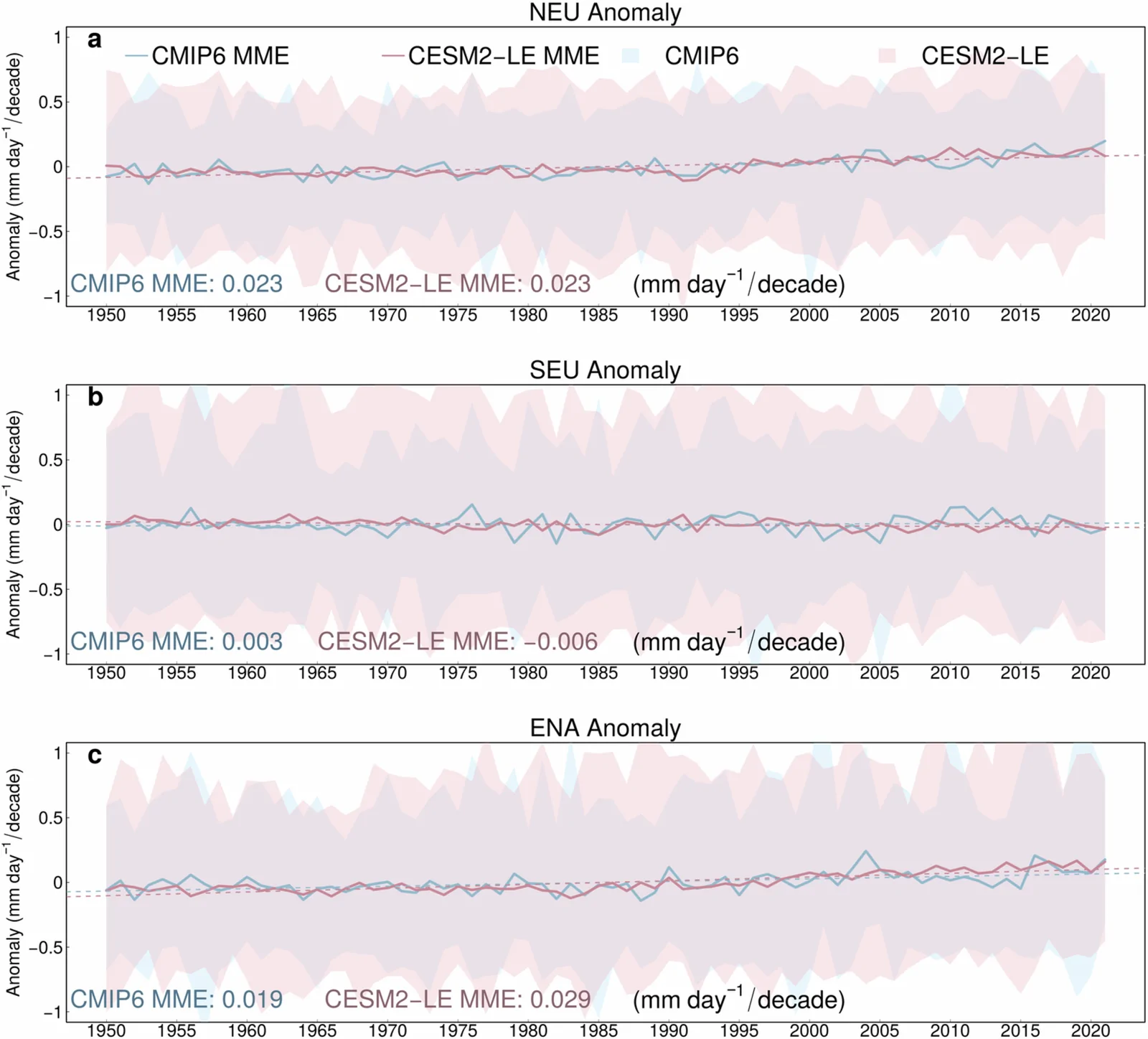
Simulated winter precipitation trends (1950–2022). **a**, Evolution and trends of winter precipitation over NEU from CMIP6 and CESM2-LE ensembles. Lines denote ensemble means (multimodel mean for CMIP6; multimember mean for CESM2-LE) and shading denotes the spread (minimum to maximum) of precipitation evolution from the CESM2-LE and CMIP6 ensembles. **b**,**c**, As in **a** but for SEU (**b**) and ENA (**c**).

Comparing the observed trends over the three selected regions with the response seen in the multimodel/multimember mean, we find a similar dipole pattern between NEU and SEU and positive trends over ENA. However, the mean response simulated by model ensembles is much weaker than observed trends over most land areas. For instance, the total forced trends are approximately 0.02 and −0.01 mm day^−1^ decade^−1^ (accounting for nearly 50% and 10% of the ERA5-based trends) over NEU and SEU according to the CESM2 ensemble mean (Extended Data Fig. 2). This raises the questions of how to understand the significant precipitation trends in observations and whether simulated winter precipitation responses to anthropogenic emissions adequately reflect the forced signal in the real world.

## Precipitation trends decomposition

**Fig. 2:**
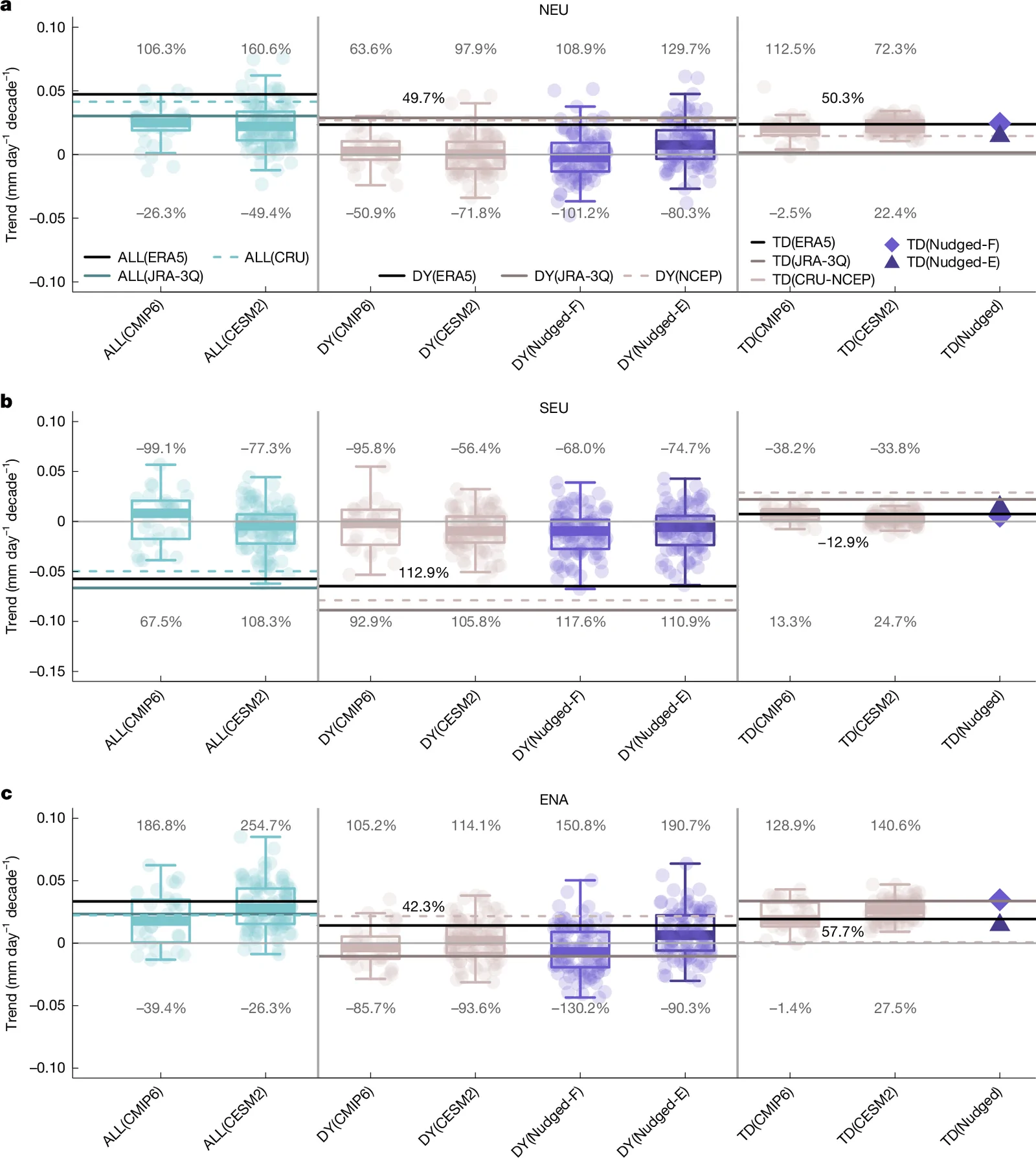
Total, dynamic and thermodynamic components of winter precipitation trends. **a**, Decomposition of winter precipitation trends (ALL) over NEU during 1950–2022 into dynamic (DY) and thermodynamic (TD) components (mm day^−1^ decade^−1^). ‘CMIP6’ and ‘CESM2’ denote estimates derived from the elastic-net model trained on the CMIP6 and CESM2 ensembles, respectively. ‘Nudged’ indicates estimates from nudged climate model experiments. ‘Nudged-F’ denotes the mean thermodynamic trends from the three paired free-running nudged simulations. ‘Nudged-E’ denotes thermodynamic trends from the nudged experiments derived with ERA5 winds. Horizontal lines represent trends from ERA5, JRA-3Q and CRU. DY(NCEP) is estimated by using SLP from the NCEP reanalysis. TD(CRU-NCEP) is estimated by subtracting the DY(NCEP) from the ALL(CRU) precipitation trends. The centre line of the box denotes the median value, the box bounds denote 25th–75th percentile values and the whiskers extend to the most extreme values within 1.5 times the interquartile range (the difference between the 75th and 25th percentile values). Grey numbers indicate the range (minimum and maximum values) of model-simulated contributions relative to total trends from ERA5 reanalysis. Black numbers denote dynamic and thermodynamic contributions to total trends (all derived from ERA5 reanalysis). **b**,**c**, As in **a** but for precipitation trends over SEU (**b**) and ENA (**c**).

**Extended Data Fig. 3:**
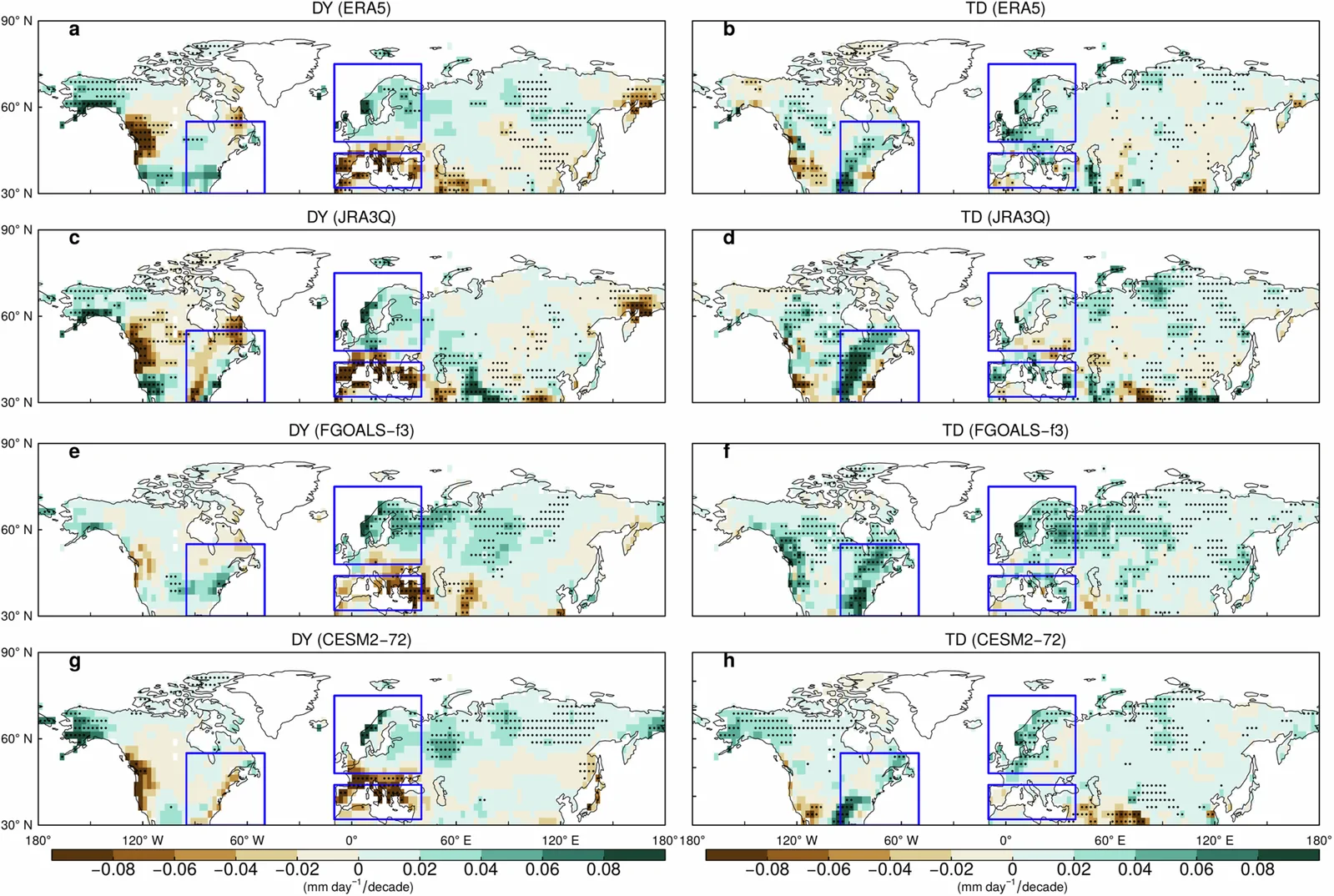
Dynamic and thermodynamic precipitation trends in reanalysis and model simulations. **a**–**d**, Dynamic (DY, **a**,**c**) and thermodynamic (TD, **b**,**d**) winter precipitation trends estimated using the elastic-net model applied to ERA5 and NCEP/CRU datasets. **e**,**f**, As in **a**,**b** but for the CMIP6 model that exhibits the highest correlation with ERA5-derived trends. **g**,**h**, As in **e**,**f** but for the corresponding member of CESM2-LE. Dot stippling represents significant trends at the 0.05 level (*P*-value < 0.05).

We disentangle the dynamic and thermodynamic drivers of precipitation trends using statistical learning models and nudged CESM2 experiments (Methods). Hereafter, we refer to the total precipitation trend (before decomposition) as ‘full precipitation trend’. The complete circulation-driven trend is referred to as the ‘full dynamic’ component—the sum of internal variability and forced circulation component. The statistical learning approach connects the circulation-induced precipitation component at each land pixel with the surrounding sea-level pressure (SLP) pattern using regularized linear regression models^22,23,36,37^. We apply this method to each land pixel in the ERA5, Japanese Reanalysis for Three Quarters of a Century (JRA-3Q) and National Centers for Environmental Prediction (NCEP) reanalyses, CMIP6 and CESM2 outputs. The resulting precipitation estimates are interpreted as driven mainly by atmospheric circulation (Fig. 2 and Extended Data Fig. 3; DY(ERA5), DY(JRA-3Q), DY(CRU), DY(CMIP6), DY(CESM2)). The residual trend, calculated as the original reanalysis-based or model-based precipitation trend minus the circulation-induced component, is interpreted as thermodynamic contributions (Fig. 2 and Extended Data Fig. 3; TD(ERA5), TD(JRA-3Q), TD(CRU), TD(CMIP6), TD(CESM2)).

In parallel, we run nudged CESM2 experiments that take dynamic conditions from free-running historical and future (SSP3.7-0) simulations with anthropogenic greenhouse gas and aerosol emissions as well as land-use changes and prescribe these dynamic conditions to control simulations without external forcing (Nudged-F). To assess the influence of observed circulation patterns, we also run CESM2 nudged experiments using horizontal winds from ERA5 (Nudged-E). Subtracting the nudged simulations with pre-industrial forcing from the nudged simulation with historical forcing yields thermodynamic precipitation trends (Fig. 2; TD(Nudged)). Further subtracting this forced thermodynamic component from the 100-member CESM2 ensemble produces full dynamic precipitation components (Fig. 2; DY(Nudged)) that are independent of the statistical learning approach (Methods).

Full dynamic precipitation trends from the statistical learning (based on both CMIP6 and CESM2 ensembles) are in line with trends from the nudging experiments (Fig. 2), supporting the robustness of the decomposition. Regionally, the contribution of circulation-induced precipitation trends varies, with observation-based dynamic components showing distinct spatial patterns across land areas (Extended Data Fig. 3a,c). To evaluate model performance, we compare these observation-based dynamic trends with the full dynamic trends from model simulations. In regions in which atmospheric circulation dominates the overall change in precipitation, the simulated trends barely encompass the observed ones. For example, in SEU, the reanalysis-based dynamic trend is −0.07 mm day^−1^ decade^−1^ and only one member of the CESM2-LE produces a dynamic trend of comparable magnitude (Fig. 2b). This highlights persistent limitations in the ability of climate models to represent large-scale circulation patterns, consistent with recent studies^6,8^.

Turning to thermodynamic precipitation trends, we find that ensemble spread across CMIP6 and CESM2 is substantially reduced compared with total trends. This reduction reflects the dominant uncertainty and variability introduced by dynamic processes. Notably, thermodynamic trends derived from climate models generally agree with those from reanalysis (Fig. 2), indicating that climate models are able to capture the thermodynamic component of the observed changes in precipitation, in line with previous findings^22^. This is in contrast with assessments of the mean climate state, which have reported large biases in simulated thermodynamic processes^38^. This suggests that climate models capture the forced thermodynamic response more faithfully than the underlying climatology.

## Physical drivers of decomposed trends

To understand the physical drivers behind decomposed precipitation trends, we examine changes in SLP, zonal wind at 500 hPa (U500), near-surface specific humidity (HUSS) and evapotranspiration using reanalysis and the best-matching simulation (Methods) from the CMIP6 and CESM2 ensembles (Supplementary Figs. 3–7). Between 1950 and 2022, zonal wind strength increases over regions with strong climatological flow, whereas SLP declines sharply over the northern North Atlantic. Reanalysis-based trends reach 0.67 m s^−1^ decade^−1^ in U500 and −0.85 hPa decade^−1^ in SLP (Supplementary Figs. 3 and 4), indicating a strengthened jet stream and enhanced dynamic precipitation over NEU. By contrast, the Mediterranean shows weakened zonal winds (less than −0.50 m s^−1^ decade^−1^) and increasing SLP (more than 0.67 hPa decade^−1^), suppressing circulation-induced precipitation over SEU. These opposing circulation trends explain the dipole pattern in precipitation between NEU and SEU. However, the best-matching simulation captures little of the observed shift in atmospheric dynamics over recent decades (Supplementary Fig. 3). The pattern correlations (above 30° N) of SLP (U500) between CMIP6 and CESM2-LE simulations and ERA5 are 0.05 ± 0.31 and 0.11 ± 0.34 (0.00 ± 0.20 and −0.01 ± 0.27), respectively (Supplementary Fig. 5). Accordingly, the simulation that best reproduces dynamic precipitation trends does not correspond to the highest correlations in either SLP or U500. This indicates limitations of climate models to reproduce observed dynamic precipitation trends in Europe.

Unlike the spatial variability and model mismatch seen with the dynamic indicators, the trends of thermodynamic indicators^39^ in reanalysis are captured well by model simulations (Supplementary Fig. 6). Specific humidity and evapotranspiration trends in ERA5 over the 1950–2022 period closely resemble those from the best-matching simulation. Thermodynamic precipitation trends are not spatially uniform (Extended Data Fig. 3b,d). Contrary to the expectation that thermodynamic drivers monotonically enhance extreme precipitation owing to increasing atmospheric moisture content under climate warming^40^, we find that thermodynamic contribution can slightly suppress seasonal mean precipitation in some areas, possibly because of reduced land–sea temperature contrast^41^. This dampening effect is also evident in evapotranspiration changes (Supplementary Fig. 6b). For example, negative evapotranspiration trends over the western Mediterranean (less than −0.03 mm day^−1^ decade^−1^) co-occur with locally suppressed thermodynamic precipitation (less than −0.05 mm day^−1^ decade^−1^ for some grid pixels). Despite modest spatial heterogeneity, the ability of climate models to reproduce trends in thermodynamic indicators (for example, *R* = 0.33 ± 0.13 between CMIP6 and ERA5 evapotranspiration patterns and *R* = 0.40 ± 0.06 between CESM2 and ERA5 patterns; Supplementary Fig. 7) supports their ability to capture changes in observed thermodynamic precipitation.

## Attribution to external forcing

**Fig. 3:**
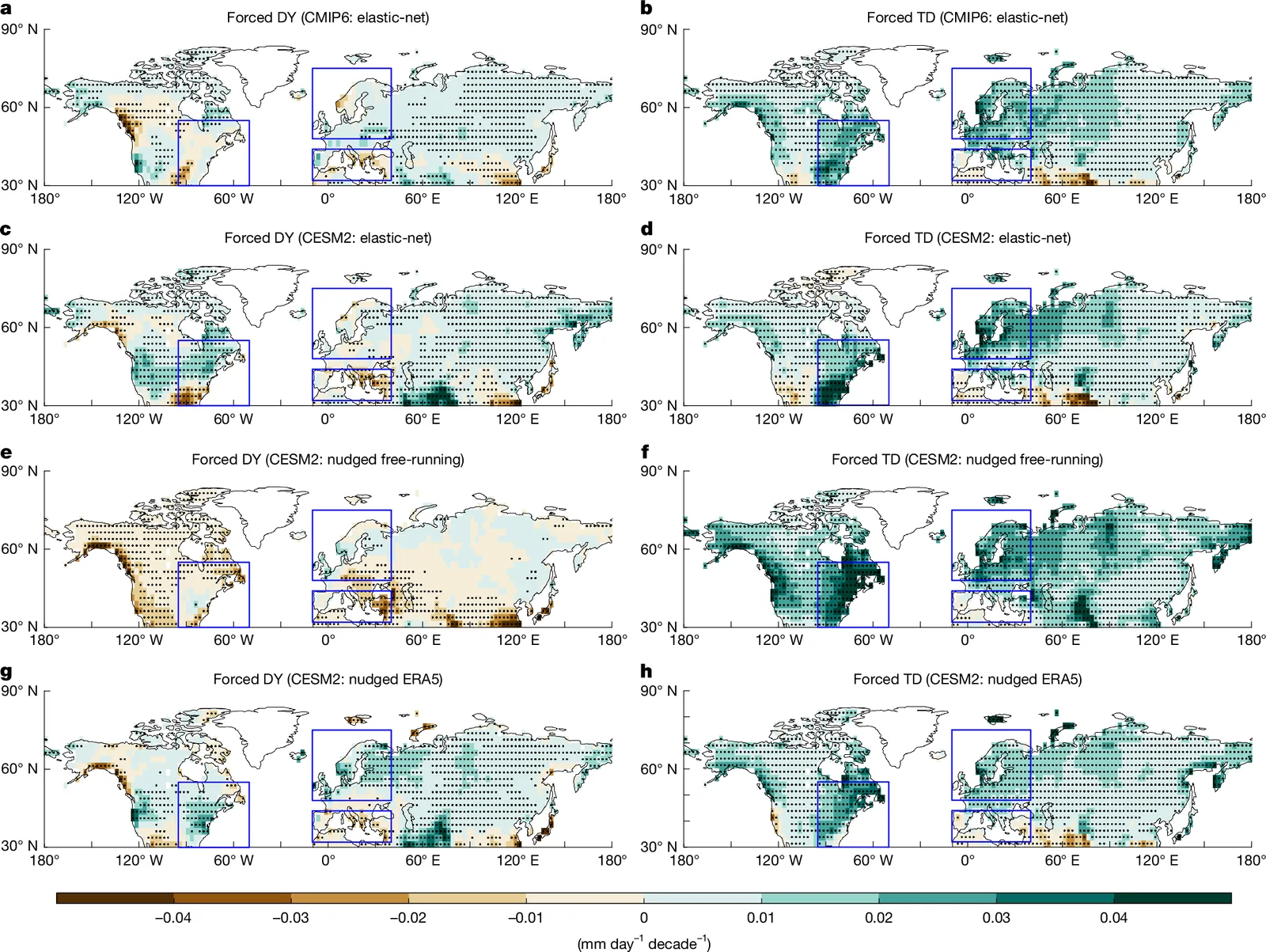
Anthropogenic forced dynamic and thermodynamic precipitation trends in winter. **a**,**b**, Anthropogenic forced dynamic (DY; **a**) and thermodynamic (TD; **b**) precipitation changes estimated using the elastic-net model trained on the CMIP6 ensemble. **c**,**d**, Same as in **a**,**b** but for the CESM2 large ensemble. **e**,**f**, As in **a**,**b** but derived from nudged climate model experiments driven with CESM2 horizontal winds (results from free-running nudged simulations and CESM2 large ensemble mean). **g**,**h**, As in **a**,**b** but derived from nudged experiments driven by ERA5 horizontal winds. Dot stippling indicates regions in which trends are significant at the 0.05 level (*P* value < 0.05).

Next we break down human-induced precipitation trends into forced dynamic and thermodynamic components. In the statistical learning framework, forced components are estimated by averaging circulation-driven and thermodynamic signals across the CMIP6 and CESM2 ensembles to remove unforced variability (Fig. 3a–d). Forced thermodynamic trends closely match the multimodel mean trends, with consistent results across CMIP6 and CESM2 statistical learning outputs (Fig. 3b,d). These trends are strongest over ENA (0.03 mm day^−1^ decade^−1^), followed by NEU (0.02 mm day^−1^ decade^−1^). Reanalysis-based trends in specific humidity and evapotranspiration are similar in magnitude to the forced trends from the CMIP6 and CESM2 ensembles (Supplementary Fig. 8), suggesting that changes in thermodynamic precipitation are mainly driven by anthropogenic forcing, with internal variability playing a secondary role.

Forced dynamic precipitation trends in climate models are weak across the domain, accounting for −23.7% to 46.0% (25th–75th percentile across grid cells) of the ERA5-based dynamic trend (Fig. 3). In SEU, in which changes in precipitation are governed mainly by atmospheric circulation, the externally forced dynamic trend accounts for only 9.7% of the full ERA5-based dynamic trend. This muted dynamic response aligns with the weak forced trends in SLP and zonal wind in climate models (Supplementary Figs. 8 and 9), which are generally less than one-quarter the magnitude of reanalysis-based trends. This mismatch suggests two possible interpretations: unforced internal variability in large-scale atmospheric circulation dominates regional precipitation trends or climate models underestimate the circulation response to anthropogenic forcing.

**Extended Data Fig. 4:**
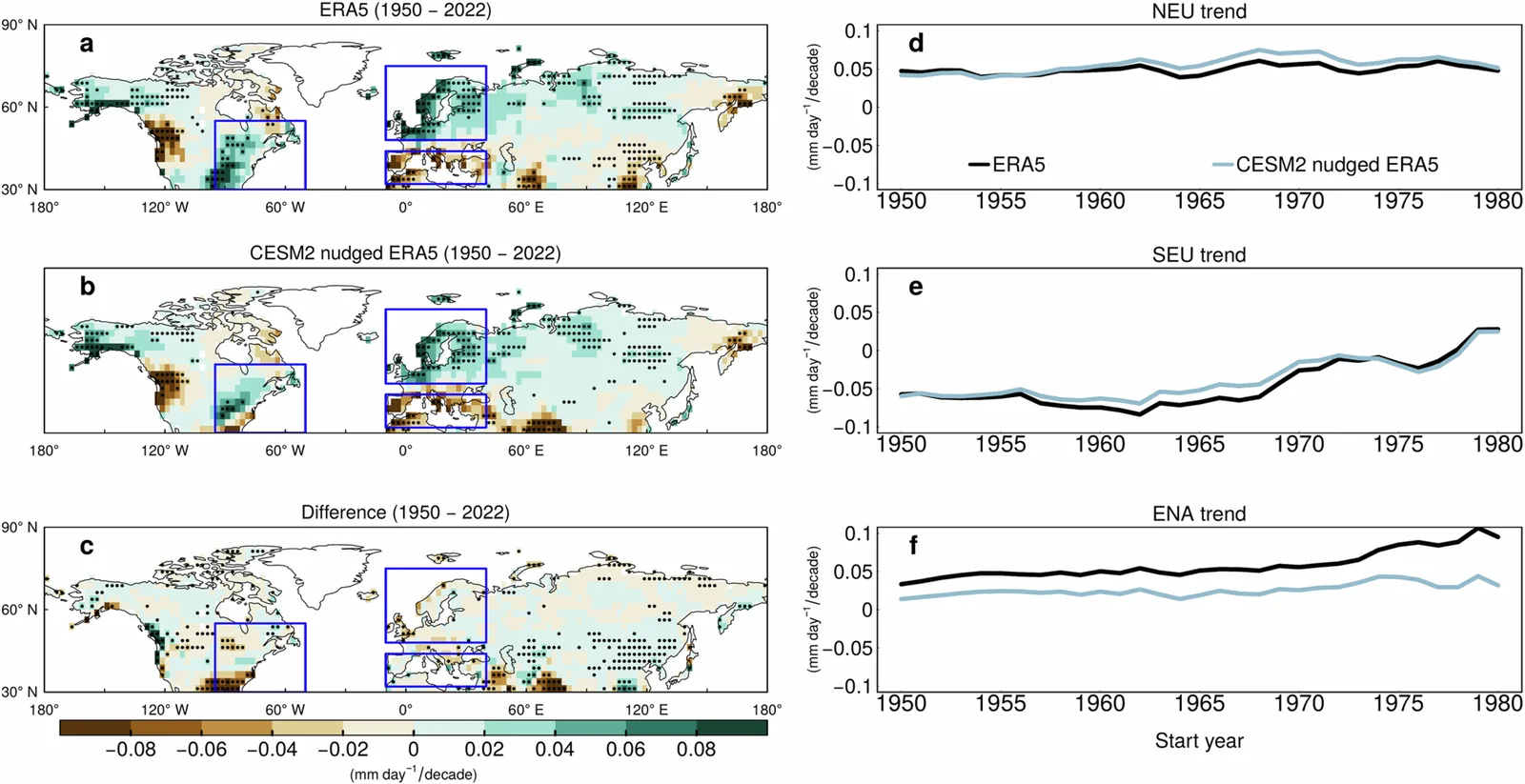
Comparison of winter precipitation trends between ERA5 and CESM2 nudged to ERA5 simulation. **a**, Winter precipitation trends from ERA5 reanalysis. **b**, Winter precipitation trends estimated by the historical CESM2 simulation driven with ERA5 wind background. **c**, Differences of winter precipitation trends between CESM2 nudged simulation and the ERA5 reanalysis. Dot stippling represents significance at the 0.05 level (*P*-value < 0.05). **d**–**f**, Magnitude of linear trends from the ERA5 reanalysis and the CESM2 nudged ERA5 simulation with anthropogenic forcing ending in 2022 as a function of start year over NEU (**d**), SEU (**e**) and ENA (**f**).

In the nudging experiments (Fig. 3e–h), the thermodynamic component is estimated directly from paired simulations that share identical circulations but differ in anthropogenic forcing. Three such pairs are available in the free-running configuration (Fig. 3e,f), whereas only one pair is available in the ERA5-nudged configuration (Fig. 3g,h). We effectively assume that internal thermodynamic variability is: (1) smaller than the forced component and (2) easily averaged out. The consistency of thermodynamic changes across the three free-running pairs supports this assumption (Supplementary Fig. 10), although it cannot be independently verified for the single ERA5-nudged pair. Nevertheless, the close agreement in total precipitation trends between the historical ERA5-nudged simulation and the ERA5 reanalysis suggests that the thermodynamic estimate derived from the ERA5-nudged pair is robust (Extended Data Fig. 4). The forced dynamic contribution is then obtained by subtracting the nudging-based thermodynamic estimate from the CESM2-LE ensemble mean. Differences between the two configurations partly reflect contrasting ocean boundary conditions: the free-running set-up uses a fully coupled ocean, whereas the ERA5-nudged configuration prescribes oceans. Despite these structural differences, both nudging approaches yield results broadly consistent with the statistical learning framework. Across all four methods, the forced thermodynamic contribution is positive over NEU and ENA (Fig. 3b,d,f,h), whereas the forced dynamic contribution is predominantly negative over SEU (Fig. 3a,c,e,g). Although the magnitude of the forced dynamic signal over SEU remains uncertain, its negative sign is robust across statistical learning approaches (Fig. 3a,c) and physical model experiments (Fig. 3e,g).

**Extended Data Fig. 5:**
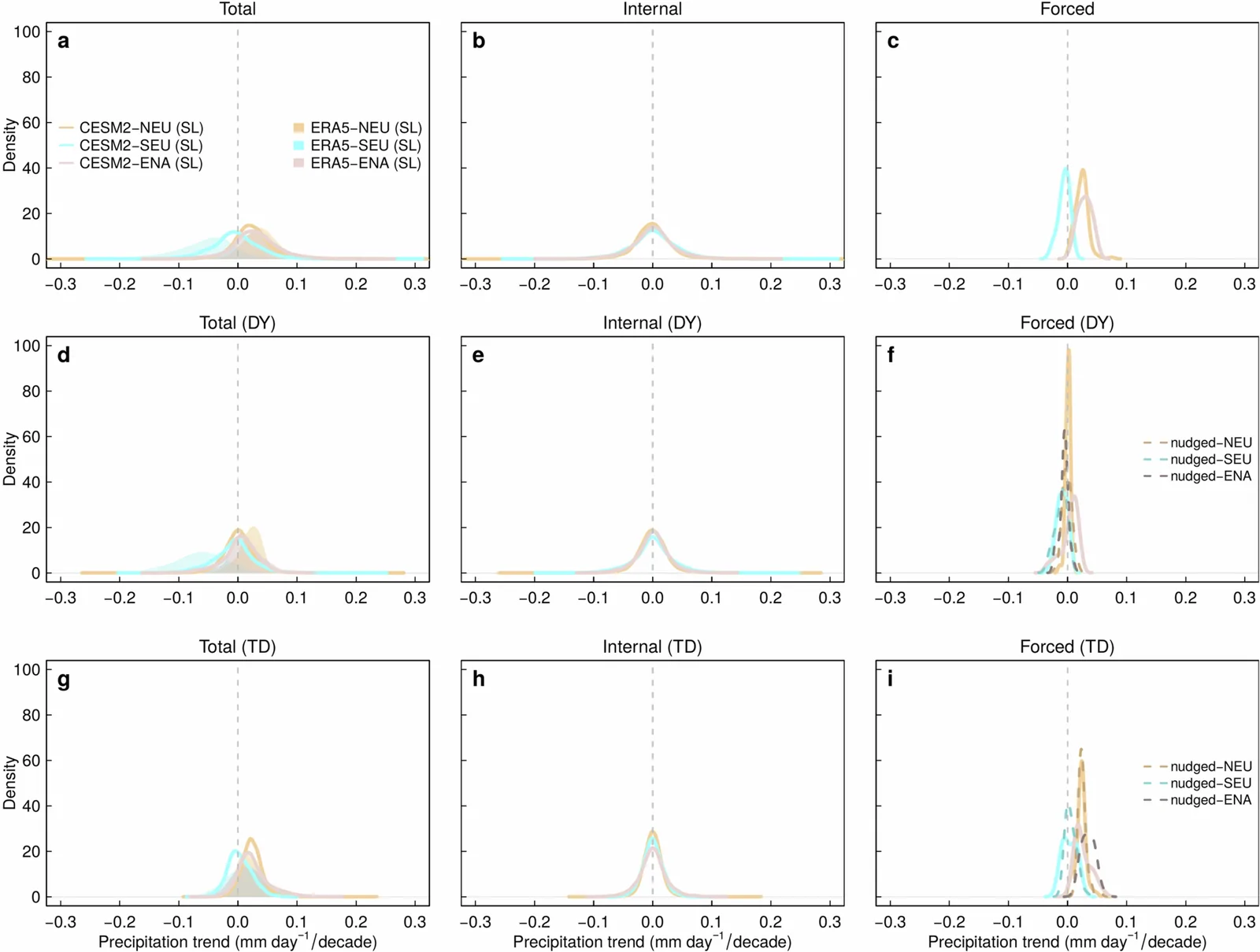
Decomposition of precipitation trends into internal, forced, dynamic and thermodynamic components. **a**–**c**, Density distributions of total, total internal and total anthropogenic forced precipitation trends. **d**–**f**, Density distributions of full dynamic (**d**), internal dynamic (**e**) and anthropogenic forced dynamic (**f**) precipitation trends. **g**–**i**, Density distributions of full thermodynamic (**g**), internal thermodynamic (**h**) and anthropogenic forced thermodynamic (**i**) precipitation trends. Density curve estimation and decomposition methods are detailed in Methods. The decomposition is based on CESM2-LE using statistical learning models and nudged climate model experiments. Because ERA5 reanalysis reflects a single realization, ideal separation into internal, forced, dynamic and thermodynamic components is not possible. For comparison, polygon-based density plots are used to represent ERA5-derived results in panels **a**, **d** and **g**.

**Extended Data Fig. 6:**
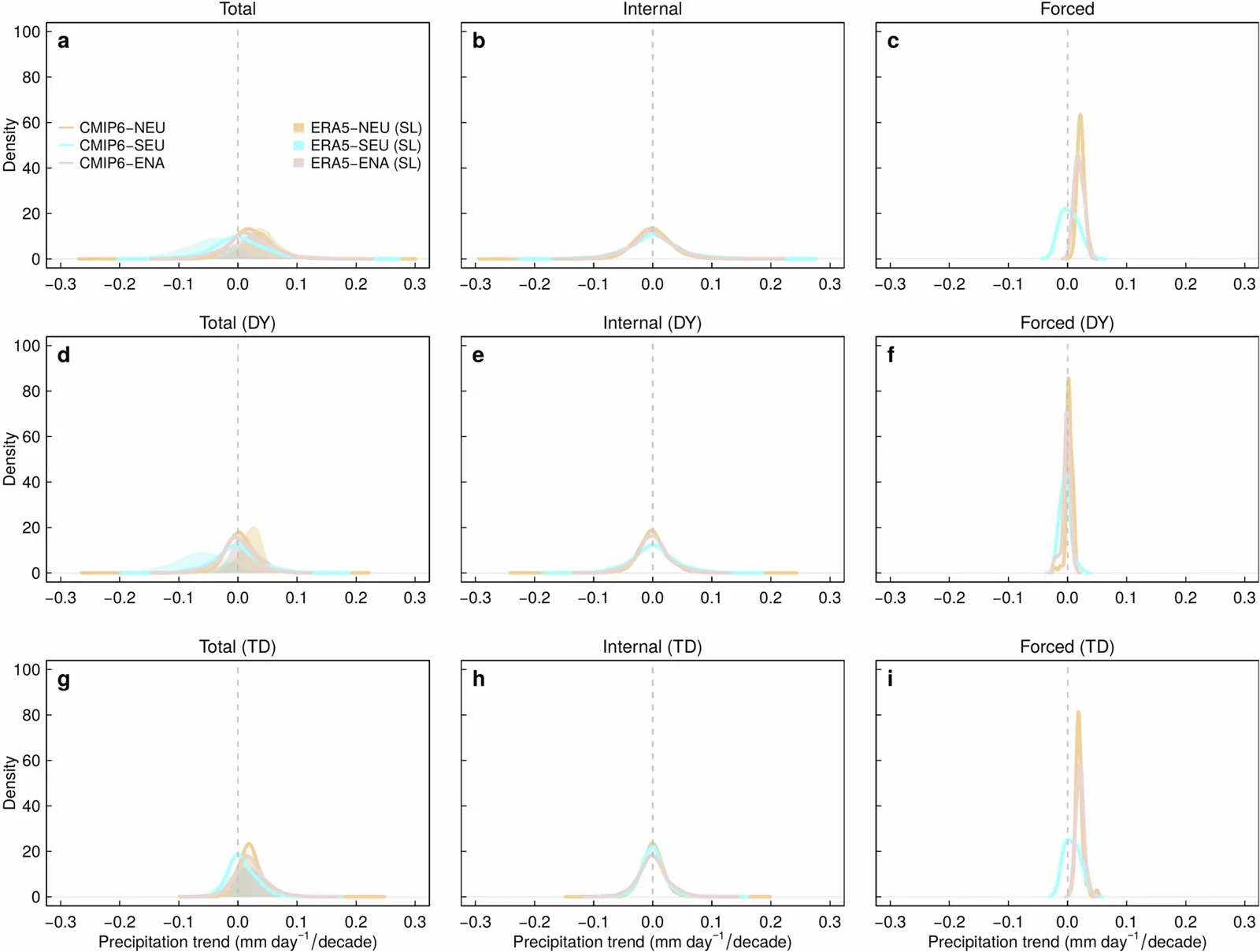
CMIP6-based decomposition of precipitation trends. **a**–**c**, Density distributions of total, total internal and total anthropogenic forced precipitation trends. **d**–**f**, Density distributions of total dynamic (**d**), internal dynamic (**e**) and anthropogenic forced dynamic (**f**) precipitation trends. **g**–**i**, Density distributions of total thermodynamic (**g**), internal thermodynamic (**h**) and anthropogenic forced thermodynamic (**i**) precipitation trends. The detailed density curves estimation and decomposition can be found in Methods. This decomposition is based on the CMIP6 ensemble using statistical learning models.

With total and forced dynamic and thermodynamic contributions quantified, next we assess the relative influence of internal variability and external forcing. We estimate the distribution (density curves) of grid-point-scale precipitation trends, both total and decomposed, across the three regions using climate model output and reanalysis (Extended Data Figs. 5 and 6). Dynamic trends are largely shaped by internal variability, with forced circulation trends contributing only a small fraction in climate model simulations. SEU stands out as a distinct case. Climate models show slightly more negative forced dynamic precipitation trends in SEU compared with other regions. At the same time, the density distributions reveal a clear mismatch between model simulations and reanalysis. In reanalysis, dynamic trends are strongly negative across most grid cells in SEU. By contrast, climate models show weakly negative circulation trends in just over half of the grid cells, driven by external forcings. Although the direction of change agrees between reanalysis (Extended Data Fig. 5d) and model-based forced response (Extended Data Fig. 5f), the much smaller modelled magnitudes leave open whether the remaining observed trend reflects internal variability or an underestimated forced circulation response. Total thermodynamic trends are partly attributable to external forcing (Extended Data Fig. 5g,i) and exceed the magnitude of the forced dynamic trends. The residual internal thermodynamic component (Extended Data Fig. 5h) shows no systematic regional signal, indicating that internal variability modulates the thermodynamic contribution at the grid-cell level without altering regional-scale trends. Note that the thermodynamic component in our framework is defined broadly to include contributions from evapotranspiration, sub-monthly transient eddies and nonlinear interactions between dynamic and thermodynamic processes.

## Outlook

Our nudged climate model experiments offer a robust framework for decomposing winter precipitation trends into dynamic and thermodynamic components across the Northern Hemisphere mid-to-high latitudes. The results align well with results from statistical learning approaches. Moreover, the statistical decomposition results are largely insensitive to the choice of climate model ensemble. As well as the multimember large ensemble (CESM2-LE), we use a multimodel ensemble based on the first realization from each model for comparison. This strategy captures a broader range of process representations across climate models and facilitates the derivation of a more generalized statistical framework, following previous studies^42,43^. The resulting spread further suggests that inter-model differences are comparable with internal variability. Future work incorporating several large ensembles from different climate models is needed to disentangle individual model responses and underlying processes^44^.

**Extended Data Fig. 7:**
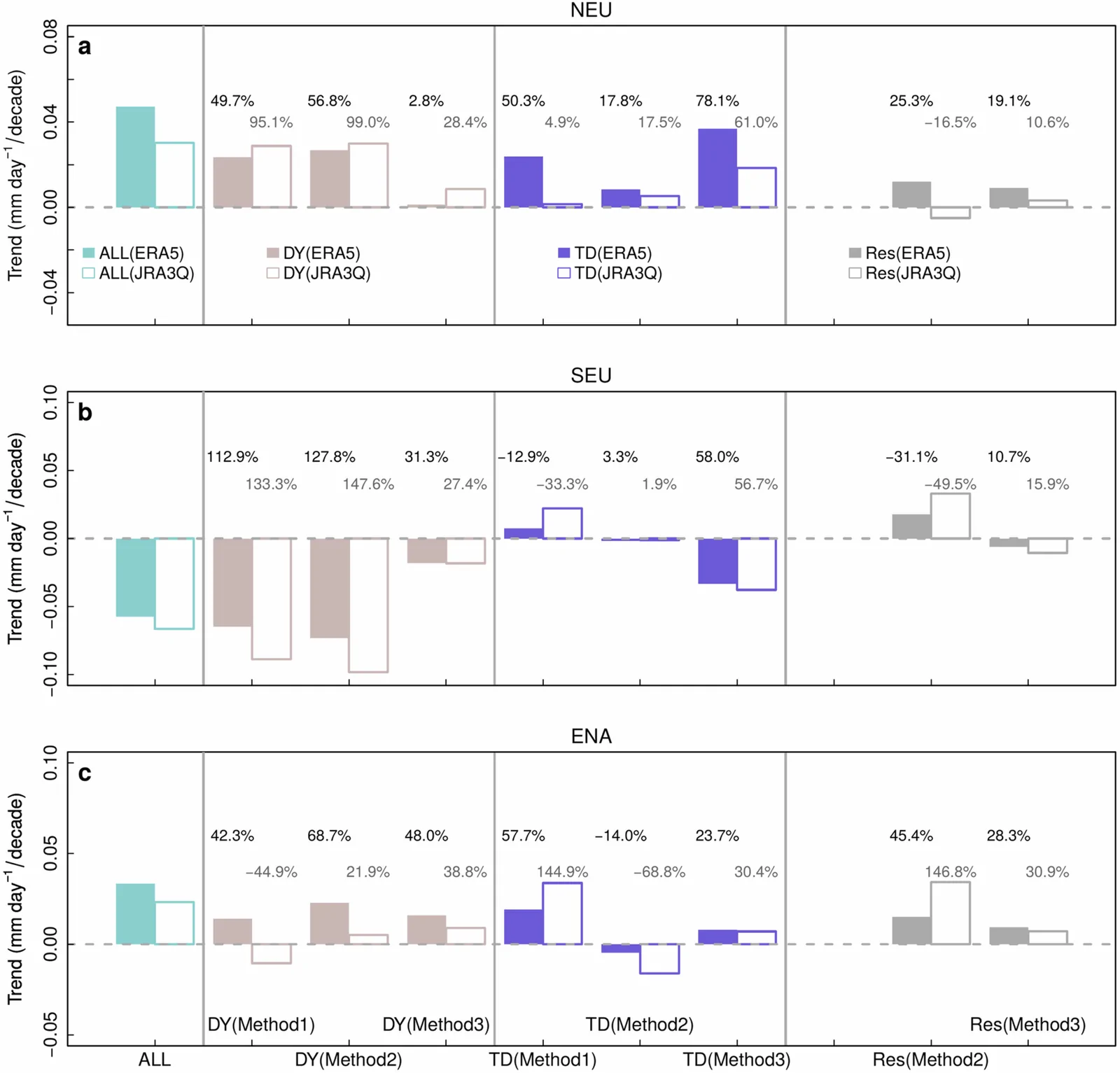
Dynamic and thermodynamic contribution to winter precipitation trends. **a**, Decomposition of winter precipitation trends over NEU during 1950–2022 (ALL) into dynamic (DY), thermodynamic (TD) and residual (Res) components using ERA5 and JRA-3Q reanalysis datasets. Method 1 represents the elastic-net model; Method 2 represents the moisture budget analysis; Method 3 represents results using daily mean 500 hPa vertical velocity as the proxy of dynamic disturbance (Methods). **b**,**c**, As in **a** but for SEU and ENA. Black (grey) numbers represent contributions derived from ERA5 (JRA-3Q).

Observed trends reflect the combined effects of internal variability, thermodynamic and dynamic precipitation response to external forcing. Simulated winter precipitation response to anthropogenic emissions does not always capture the full magnitude of signals observed in the real world, although internal variability may account for much of this discrepancy. The thermodynamic response of precipitation to external forcings is relatively robust across models, with particularly strong signals over ENA. This helps to reconcile observations and climate models: where thermodynamic contributions dominate local precipitation trends, multimodel or multimember mean projections provide high-fidelity estimates of future climate. In this study, thermodynamic influence is defined broadly to encompass all non-circulation factors, such as changes in lapse rate, land–sea thermal contrast, evapotranspiration, humidity, transient eddies and nonlinear effects. This definition is reflected in the thermodynamic contributions identified by the moisture budget analysis^45,46^. This moisture budget method typically restricts thermodynamic effects to temperature-related and humidity-related changes in vertical and horizontal moisture advection, thus yielding different thermodynamic partitioning relative to our statistical learning approach (Extended Data Fig. 7 and Supplementary Fig. 11). By contrast, dynamic contributions to total precipitation trends are generally consistent across the two methods, supporting the robustness of the decomposition. To provide further comparison, we also apply an empirical binning approach using 500-hPa vertical velocity as the proxy for the strength of dynamic disturbance^47^. Although the magnitude of the dynamic contribution obtained from this method is systematically smaller, the sign is consistent with results from the statistical learning and moisture budget. This further confirms that the circulation-wetting in NEU/ENA and circulation-drying in SEU is not an artefact.

Circulation-induced precipitation trends in reanalysis show substantial spatial heterogeneity and are particularly dominant over SEU, in which they account for most of the total trend, as indicated by both the ERA5 and JRA-3Q datasets (Extended Data Fig. 3a,c). However, climate model ensembles capture little of this variability. The spread of simulated dynamic trends does not encompass the observed range and the circulation response to external forcing remains consistently weak. As a result, the fraction of forced dynamic precipitation trends relative to ERA5-based estimates (full dynamic precipitation trends) is small across circulation-dominated areas such as SEU (Supplementary Fig. 12).

**Extended Data Fig. 8:**
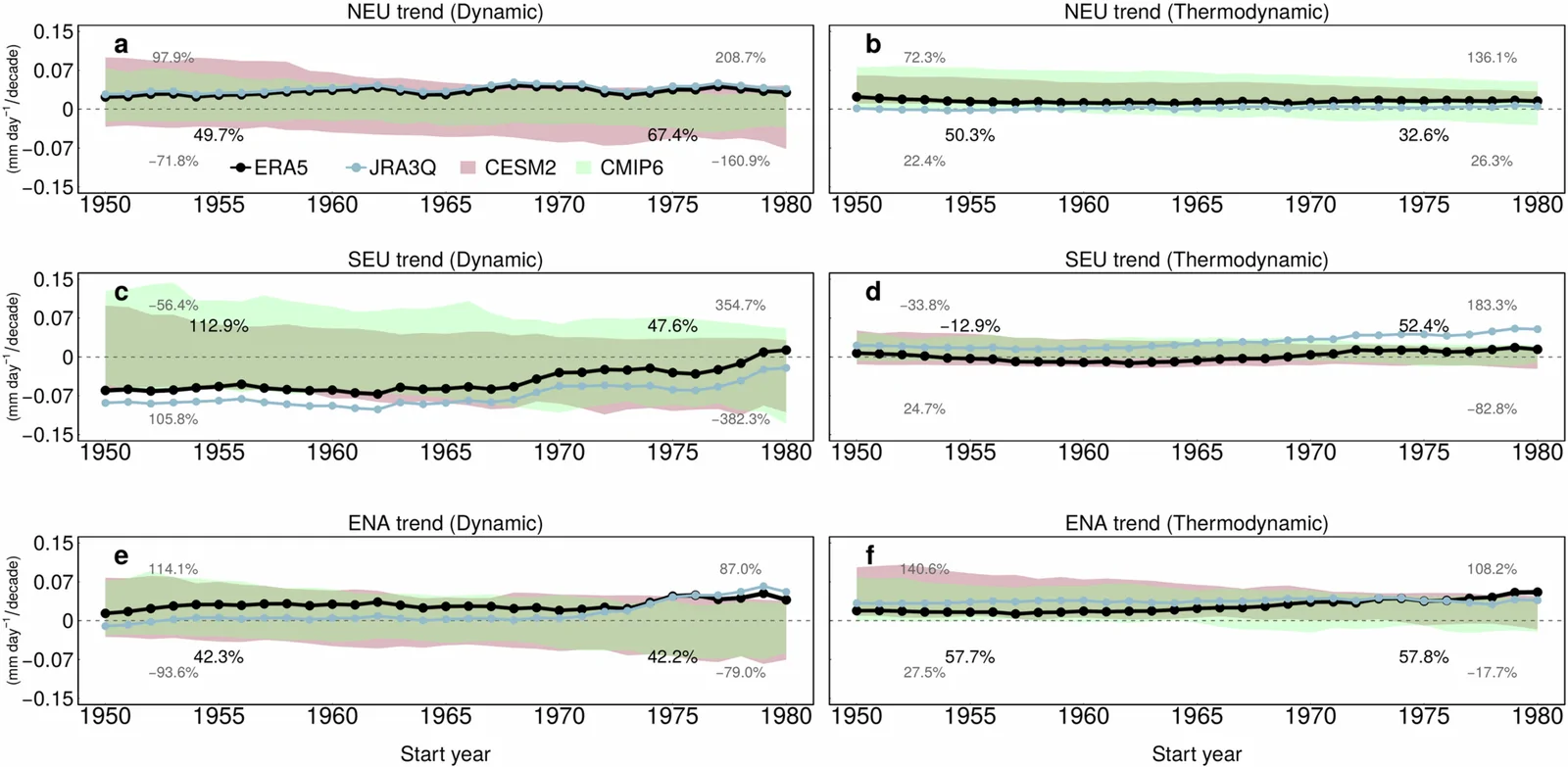
Evolution of dynamic and thermodynamic precipitation trends with varying start year. **a**,**b**, Magnitudes of linear dynamic (**a**) and thermodynamic (**b**) precipitation trends ending in 2022 as a function of start year, based on ERA5, JRA-3Q, CMIP6 and CESM2 ensembles over NEU. Pink (blue) shading denotes the range (minimum to maximum) of trends from the CESM2-LE (CMIP6) ensemble. **c**,**d**, As in **a**,**b** but for dynamic (**c**) and thermodynamic (**d**) precipitation trends over SEU. **e**,**f**, As in **a**,**b** but for dynamic (**e**) and thermodynamic (**f**) precipitation trends over ENA. The grey numbers show dynamic (**a**,**c**,**e**) versus thermodynamic (**b**,**d**,**f**) contributions (for the start years of 1950 and 1980) from CESM2-LE relative to total precipitation trends using ERA5 as the benchmark; the black numbers show respective contributions of decomposed dynamic versus thermodynamic trends estimated from the ERA5 reanalysis.

**Extended Data Fig. 9:**
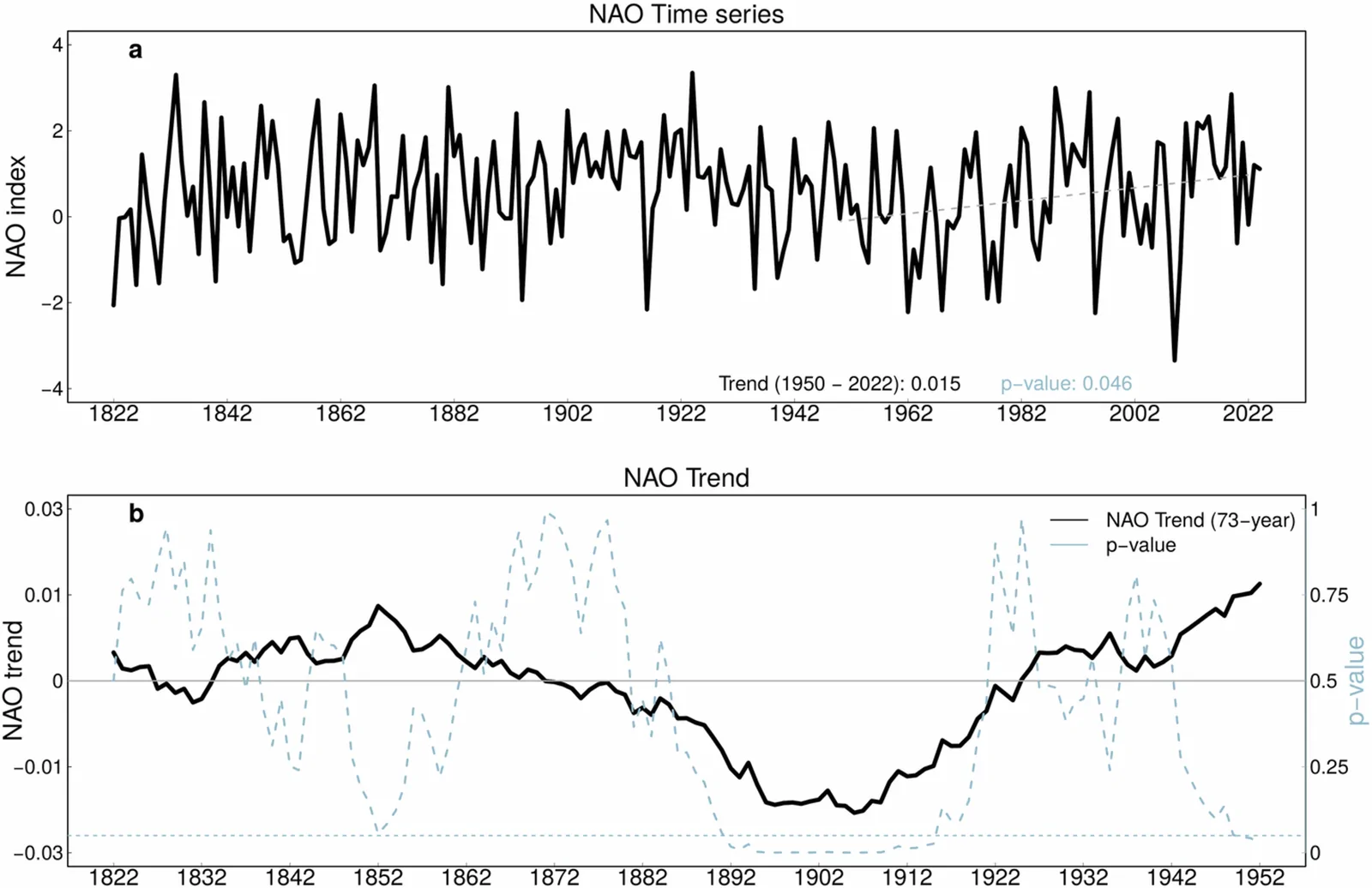
NAO index and trends. The NAO index is from Jones et al./CRU^58^ and is averaged over boreal winter. **a**, Time series of the NAO index in boreal winter. The regression line corresponds to linear trend over 1950–2022. **b**, The NAO trend estimated using a 73-year moving window. *x*-axis represents the first year of each window.

Our results are consistent with Vicente-Serrano et al.^5^, who analysed more than 23,000 stations spanning 1871–2020 and found largely stationary SEU winter precipitation dominated by NAO variability. Over SEU, the thermodynamic contribution is small and spatially incoherent, leaving the total precipitation trend largely determined by circulation changes. The forced dynamic signal in climate models is weak relative to internal variability, making detection inherently difficult. Whether this signal is genuinely small or systematically underestimated by models cannot be resolved from the observational record alone. However, as warming continues, the dynamic signal may eventually break the long-term stationarity documented in ref. ^5^. Recent evidence that multidecadal NAO increases in response to greenhouse gases^7^ supports this interpretation, indicating the potential emergence of a forced circulation signal. The detectability of such a signal depends critically on the period examined. We find substantial drying over SEU for 1950–2022, whereas shorter periods show weaker or no trends (Supplementary Fig. 2). Similar circulation-associated drying has been reported for 1921–2015 (ref. ^22^) and 1959–2021 (ref. ^48^), yet is not reproduced by CMIP6 models. By contrast, ref. ^49^ shows that SEU precipitation trends during 1980–2022 are unimportant, with a weak dynamic component that is well captured by model ensembles. This dependence on period reflects strong internal variability rather than directly constraining the magnitude of the forced component (Extended Data Fig. 8). Strong multidecadal NAO oscillations may mask or amplify a moderate forced signal (Extended Data Fig. 9), which is consistent with an anthropogenic contribution being detectable only in certain sufficiently long and recent periods.

**Extended Data Fig. 10:**
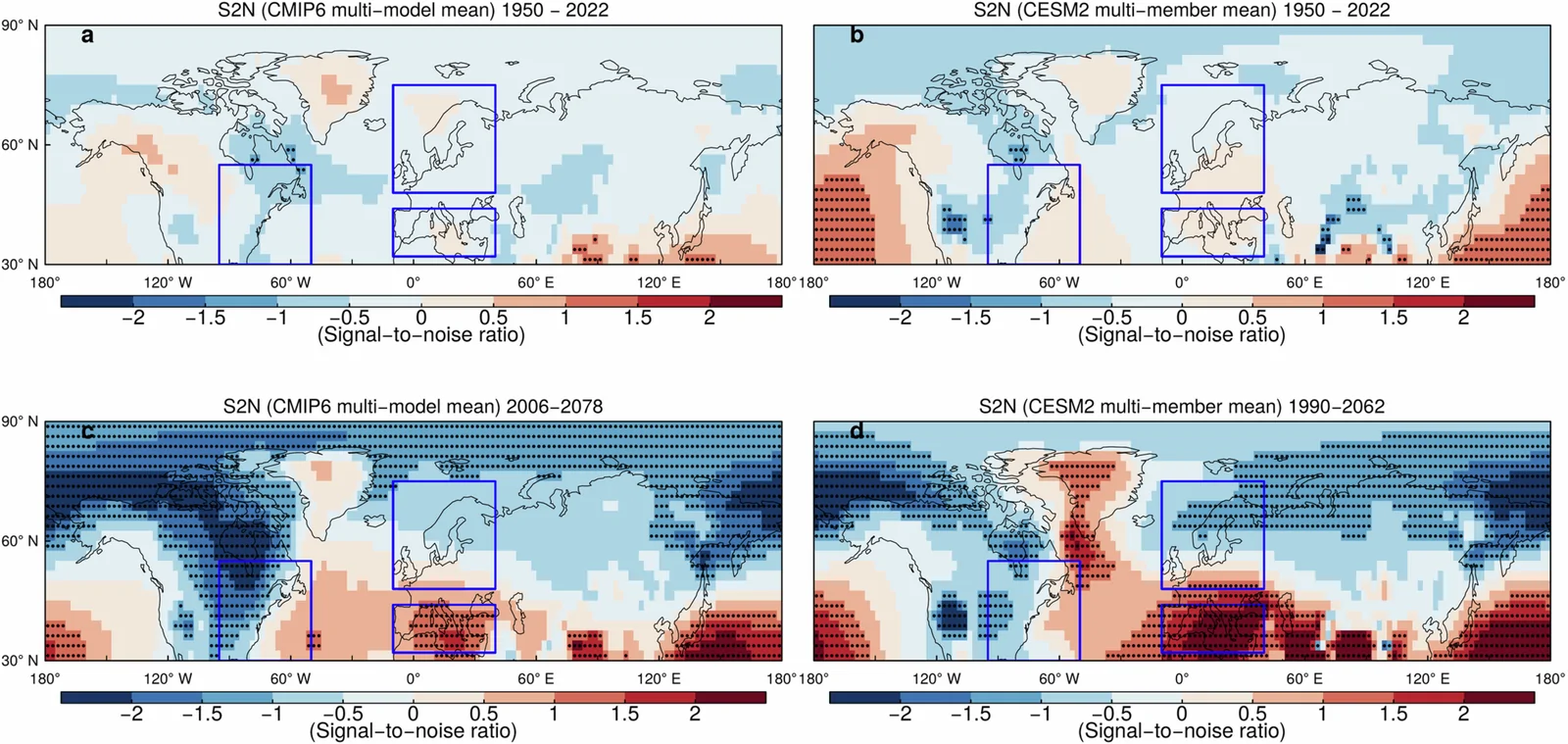
Signal-to-noise ratios of SLP response to human emissions. **a**,**b**, Signal-to-noise ratios of SLP trends from CMIP6 multimodel mean (**a**) and from CESM2 multimember mean (**b**) during 1950–2022. **c**,**d**, As in **a**,**b** but for future periods in which modelled trends show the highest correlation (Pearson correlation coefficient) with ERA5 trends over 1950–2022. Dot stippling indicates regions in which the signal-to-noise ratio is higher (lower) than 1 (−1).

Looking ahead, projections under increasing anthropogenic emissions imply a continued weakening of circulation over the Mediterranean, manifested as a strengthened signal-to-noise ratio^50^ of SLP trends (Extended Data Fig. 10). The projected decline in dynamic precipitation trends over the SEU, also reported in ref. ^32^, closely resembles observed trend patterns. This correspondence can arise because internally driven variability in observations happens to resemble the projected forced response and/or because climate models underestimate the magnitude of the forced dynamic contribution. In either case, confidence in regional precipitation projections depends critically on the reliable representation of large-scale circulation.

## Methods

### Reference data and climate model simulations

We use several precipitation datasets to examine changes under climate warming, including ERA5 (ref. ^51^), JRA-3Q (ref. ^52^), the high-resolution gridded multivariate climate dataset CRU TS v4 (ref. ^53^) (1950–2022), GPCC^54^ full_data_monthly_v2022 (1950–2020) and monitoring_v2022 (2021–2022), the Multi-Source Weighted-Ensemble Precipitation (MSWEP)^55^ and the Modern-Era Retrospective Analysis for Research and Applications, version 2 (MERRA-2; 1980–2022)^56^. To assess the changes in dynamic contributions to observed terrestrial precipitation, we collect monthly SLP and zonal wind data from ERA5 and JRA-3Q and include SLP from NCEP–NCAR reanalysis^57^ for comparison. To perform moisture budget analysis, we also retrieved surface pressure and horizontal winds (*u*, *v*), vertical velocity (*ω*) and specific humidity for 37 pressure levels from ERA5 and 45 pressure levels from JRA-3Q. Finally, daily 500-hPa vertical velocity and daily precipitation are also retrieved from ERA5 and JRA-3Q to serve as the input of an empirical binning method, offering a simple check of thermodynamic and dynamic decomposition of precipitation. Also, thermodynamic processes are examined using monthly 2 m dew point temperature and evapotranspiration from ERA5. The NAO index is from Jones et al./CRU^58^.

We use a large ensemble from the CMIP6, using the first realization (r1i1p1f1) of 29 models: ACCESS-CM2, ACCESS-ESM1-5, AWI-CM-1-1-MR, BCC-CSM2-MR, CAMS-CSM1-0, CanESM5, CAS-ESM2-0, CESM2, CESM2-WACCM, CMCC-CM2-SR5, CMCC-ESM2, EC-Earth3, EC-Earth3-AerChem, EC-Earth3-Veg, EC-Earth3-Veg-LR, FGOALS-f3-L, FGOALS-g3, GFDL-ESM4, INM-CM4-8, INM-CM5-0, IPSL-CM6A-LR, KACE-1-0-G, MIROC6, MPI-ESM1-2-HR, MPI-ESM1-2-LR, MRI-ESM2-0, NorESM2-LM, NorESM2-MM and TaiESM1. To account for internal variability, we also use the CESM2-LE^34^, which includes 100 members. This ensemble supports decomposition of precipitation changes into circulation-induced and residual thermodynamic components using our statistical learning model.

Simulations are extracted under the historical (1950–2014) and SSP3-7.0 (SSP370) scenarios (2015–2022), both of which include anthropogenic and natural forcings such as greenhouse gases, aerosols, solar variability and land-use changes. SSP370 is selected to maintain consistency with CESM2-LE, which is forced under the same scenario. Monthly outputs of precipitation (PRE), SLP, U500, HUSS, surface latent heat (LE) and surface temperature (*T*) are interpolated to a 2.5° × 2.5° longitude–latitude grid.

To assess the robustness of climate simulations, we investigate precipitation types and their climatological characteristics. Winter precipitation in these northern mid-latitudes is dominated by large-scale processes^44,45^. The fraction of large-scale precipitation relative to the total (that is, large-scale plus convective) exceeds 70.0% in 81.4% of land areas across the Northern Hemisphere (Supplementary Fig. 13). Similar results are found using the MERRA-2 reanalysis dataset (1980–2022). We then estimate the climatological averages of large-scale and total precipitation, as well as their ratio (large-scale fraction) from climate model simulations (Supplementary Fig. 14). We find them to be consistent with the climatology of reanalysis datasets. This agreement supports the robustness of climate models in characterizing precipitation physics.

### Constrained climate model (experiment 1)

To investigate the anthropogenic forced thermodynamic effect on changes in precipitation, we conduct a series of nudged physical climate experiments. Specifically, we constrain the large-scale circulation patterns of an ‘unforced’ (pre-industrial) simulation towards a coupled CESM2 simulation under the historical (1850–2014) and the SSP370 (2015–2100) scenarios. The first step involves fully coupled simulations with free-running atmosphere, prescribed CO_2_ emissions and evolving land-use and land-cover distributions, following the CESM2-LE protocol using the CAM6.3 atmospheric component. Then we run paired ‘unforced’ simulations under constant 1850-level forcings, nudging the horizontal winds ([*U*, *V*]) towards the coupled simulations. Both CO_2_ and land-use parameters remain fixed at 1850 levels throughout the 1850–2100 period. Nudging follows the standard ‘linear-weak’ configuration (see CAM6.3 User’s Guide). To test sensitivity to initial climate conditions, we run three paired simulations: coupled Run1300 (Run 1300), coupled Run1400 (Run 1400), coupled Run1500 (Run 1500), along with corresponding nudged simulations (Nudging 1300, Nudging 1400, Nudging 1500) using full-atmosphere wind nudging. To further assess robustness, we include two more nudged simulations based on Run1300: Nudging 1300L (nudging winds down to 691 hPa) and Nudging 1300H (nudging only down to 322 hPa). Differences between the coupled and nudged simulations isolate the thermodynamic influence of anthropogenic forcing (specifically, changes in atmospheric CO_2_ and land-use patterns) as follows:

1 $${F}_{\mathrm{TD-PRE}}^{i,j}={{\rm{Run}}}_{{\rm{PRE}}}^{i,j}-{{\rm{Nudging}}}_{{\rm{PRE}}}^{i,j}$$

2 $${T}_{\mathrm{DY,nug-PRE}}^{i,j}={T}_{{\rm{PRE}}}^{i,j}-{F}_{\mathrm{TD-PRE}}^{\mathrm{ng-avg},j}$$

3 $${F}_{\mathrm{DY,nug-PRE}}^{j}=\frac{1}{n}\mathop{\sum }\limits_{i=1}^{n}{T}_{\mathrm{DY,nug-PRE}}^{i,j}$$

in which ${F}_{\mathrm{TD-PRE}}^{i,j}$ denotes the idealized anthropogenic forced thermodynamic precipitation and SLP for the *j*th grid cell in the *i*th run, which includes simulations 1300, 1400, 1500, 1300L and 1300H. Across nudged experiments with different initial conditions, the results remain consistent as long as the full atmospheric horizontal winds are nudged (Supplementary Figs. 15 and 16). When nudging winds from the upper atmosphere down to the lower troposphere, the results still resemble those from full-atmosphere nudging. However, nudging only the upper troposphere produces discernible differences in the thermodynamic effect. ${T}_{{\rm{PRE}}}^{i,j}$ represents the precipitation for the *j*th grid cell in the *i*th simulation of the 100-member CESM2 ensemble. The term ${F}_{\mathrm{TD-PRE}}^{\mathrm{ng-avg},j}$ refers to the forced thermodynamic precipitation averaged across runs 1300, 1400 and 1500. ${T}_{\mathrm{DY,nug-PRE}}^{i,j}$ denotes the full dynamic precipitation for the *i*th run (*i* ranges between 1 and 100) and ${F}_{\mathrm{DY,nug-PRE}}^{j}$ represents the anthropogenic forced dynamic precipitation component derived from climate model experiments. In this framework, we assume additivity of trend components, disregard long-term impacts from natural forcings such as volcanic activity and acknowledge that the estimated dynamic changes, independent of the statistical learning approach, may still reflect feedback and interactions between dynamics and thermodynamics.

### Constrained climate model with ERA5 winds (experiment 2)

To test further the robustness of the nudged climate model experiments, we run an extra experiment in which CESM2 atmospheric horizontal winds are nudged to ERA5 reanalysis fields. Both the pre-industrial and forced (historical + SSP370) simulations use a prescribed ocean from the Met Office Hadley Centre Sea Ice and Sea Surface Temperature dataset (HadISST)^59^, with horizontal winds nudged to ERA5 above 700 hPa. Specifically, this experiment is conducted in an atmosphere-only configuration (AMIP style) with prescribed ocean boundary conditions. The sea-surface temperature for the pre-industrial run is estimated by removing the forced component from HadISST using a low-frequency filtering method^60,61^. This approach preserves the year-to-year variability while altering the low-frequency background state. The relationship between sea-surface temperature and sea ice is derived from the HadISST dataset and the pre-industrial sea-ice concentration is subsequently generated on the basis of the reconstructed pre-industrial sea-surface temperature. We find that precipitation trends from the nudged CESM2 atmospheric general circulation model simulation closely match those from ERA5, confirming the robustness of externally applied forcing in climate models (Extended Data Fig. 4). As in experiment 1, the difference between forced and pre-industrial simulations is interpreted as the anthropogenic forced thermodynamic component. The forced dynamic precipitation component is then estimated by subtracting this forced thermodynamic component from the CESM2-LE ensemble mean. Note that the ERA5-nudging experiment uses prescribed sea-surface conditions, whereas CESM2-LE is fully coupled. Consequently, the imposed constraint from ERA5 winds does not necessarily yield a cleaner estimate of the forced dynamic component, as atmosphere–ocean coupling processes that interact with circulation and precipitation are not consistently represented across the two frameworks. Details of the estimation of thermodynamic and dynamic components in the nudging experiments are provided in Extended Data Table 1.

### Statistical learning to simulate the dynamic induced precipitation

We estimate the influence of atmospheric circulation variability on monthly precipitation using an elastic-net regression model^23,62,63^. This approach is based on traditional regularized linear models by balancing penalties on the sum of absolute regression coefficients and the sum of squared regression coefficients, as follows:

4 $${\gamma }^{\mathrm{elastic-net}}={\rm{argmin}}\left\{{\rm{RSS}}+\lambda \mathop{\sum }\limits_{j=1}^{p}[(1-\alpha ){\gamma }_{j}^{2}+\alpha |{\gamma }_{j}]\right\}$$

in which *γ* denotes the regression coefficients, *λ* is the shrinkage parameter and *α* controls the balance between the lasso and ridge penalties. If *α* = 1, it is the ‘lasso’ penalty; if *α* = 0, it is the ridge regression; if 0 < *α* < 1, it is the elastic-net model, which combines the lasso and ridge penalties. We apply this elastic-net model to each of the global land grids.

For each grid, we define a 20° × 20° spatial domain centred on the grid cell for the SLP field used in prediction. We derive anomalies for SLP and precipitation relative to monthly means, train the elastic-net model and cross-validate it using 300 years of data from each of the 29 CMIP6 models under the pre-industrial scenario. Then the trained parameters are applied to CMIP6 and CESM2 simulations under historical and SSP370 scenarios, using the domain SLP as input, to estimate circulation-induced monthly precipitation anomalies (${T}_{\mathrm{DY-PRE}}^{i}$). Subtracting these from total precipitation anomalies yields the thermodynamic component (${T}_{\mathrm{TD-PRE}}^{i}$). Training on the pre-industrial scenario avoids contamination from anthropogenic forcing. We also train the model using three nudged CESM2 runs (Nudging 1300, Nudging 1400 and Nudging 1500) and 30 members of CESM2-LE, finding consistent parameter estimates across land grids. Also, whether SLP is detrended or not does not affect the results. Model performance is evaluated by the fraction of variance explained (*R*^2^) over the 1950–2022 period (Supplementary Fig. 17).

The statistical learning framework enables full decomposition of changes in precipitation into anthropogenic forced thermodynamic and dynamic components, as well as internal thermodynamic and dynamic contributions, as follows:

5 $${T}_{\mathrm{DY-PRE}}^{i,j}={f}_{\mathrm{elastic-net}}^{j}({{\rm{SLP}}}^{i,j})$$

6 $${T}_{\mathrm{TD-PRE}}^{i,j}={T}_{{\rm{PRE}}}^{i,j}-{T}_{{\rm{DY}}-{\rm{PRE}}}^{i,j}$$

7 $${F}_{\mathrm{DY-PRE}}^{j}=\frac{1}{n}\mathop{\sum }\limits_{i=1}^{n}{T}_{\mathrm{DY-PRE}}^{i,j}$$

8 $${F}_{\mathrm{TD-PRE}}^{j}=\frac{1}{n}\mathop{\sum }\limits_{i=1}^{n}{T}_{\mathrm{TD-PRE}}^{i,j}$$

in which ${T}_{{\rm{PRE}}}^{i,j}$, ${T}_{\mathrm{DY-PRE}}^{i,j}$ and ${T}_{\mathrm{TD-PRE}}^{i,j}$ represent the full, dynamic and thermodynamic components, respectively, of precipitation for the *j*th grid in the *i*th simulation (29 CMIP6 model ensemble or 100-member CESM2 ensemble). ${f}_{\mathrm{elastic-net}}^{j}$ represents the trained elastic-net model for the *j*th grid and SLP^*i*,*j*^ denotes the SLP domain centred on the *j*th grid in the *i*th simulation. ${F}_{\mathrm{DY-PRE}}^{j}$ represents the anthropogenic forced dynamic component and ${F}_{\mathrm{TD-PRE}}^{j}$ is the anthropogenic forced thermodynamic component.

More specifically, the total forced precipitation component is estimated by averaging outputs from the CMIP6 or CESM2 ensemble. Precipitation components driven by internal climate variability are obtained by subtracting the total forced component from each individual simulation with the ensemble. Full dynamic precipitation components are derived from surrounding SLP patterns. The forced dynamic precipitation component is calculated by averaging these dynamic precipitation estimates across the ensemble, whereas internal dynamic components are obtained by subtracting the forced dynamic signal from the full dynamic component. Thermodynamic precipitation components are determined by subtracting dynamic contributions from total precipitation. The forced thermodynamic precipitation component is estimated by averaging these thermodynamic values across the ensemble, and the internal thermodynamic component is calculated by subtracting the forced thermodynamic signal from the total thermodynamic component.

### Moisture budget analysis and an empirical binning method

To evaluate the robustness of precipitation decomposition, we use two complementary approaches. First, the moisture budget diagnostics were computed at monthly resolution. Following refs. ^45,46^, the changes in water availability (precipitation minus evapotranspiration, *P*′ − *E*′) are balanced by changes in horizontal (−<*V*_h_∇_h_*q*>′) and vertical (−<*ω∂*_p_*q*>′) moisture advection, as well as a residual (*ε*′, including sub-monthly transient eddies, nonlinear effects and so on):

9 $${P}^{{\prime} }-{E}^{{\prime} }=-{ < {V}_{{\rm{h}}}{\nabla }_{{\rm{h}}}q > }^{{\prime} }-{ < \omega {\partial }_{{\rm{p}}}q > }^{{\prime} }+{\varepsilon }^{{\prime} }$$

10 $$-{ < \omega {\partial }_{{\rm{p}}}q > }^{{\prime} }=- < \bar{\omega }{\partial }_{{\rm{p}}}{q}^{{\prime} } > - < \bar{q}{\omega }^{{\prime} }{\partial }_{{\rm{p}}} > +{\varepsilon }_{\omega }^{{\prime} }$$

11 $$-{ < {V}_{{\rm{h}}}{\nabla }_{h}q > }^{{\prime} }=- < \bar{{V}_{{\rm{h}}}}{\nabla }_{{\rm{h}}}{q}^{{\prime} } > -\bar{q}{{V}_{{\rm{h}}}}^{{\prime} }{\nabla }_{{\rm{h}}} > +{\varepsilon }_{V}^{{\prime} }$$

in which $- < \bar{\omega }{\partial }_{{\rm{p}}}{q}^{{\prime} } >$ ($- < \bar{{V}_{{\rm{h}}}}{\nabla }_{{\rm{h}}}{q}^{{\prime} } >$) and $- < \bar{q}{\omega }^{{\prime} }{\partial }_{{\rm{p}}} >$ ($- < \bar{q}{{V}_{{\rm{h}}}}^{{\prime} }{\nabla }_{{\rm{h}}} >$) denote thermodynamic and dynamic components from the vertical (horizontal) moisture advection, respectively. ${\varepsilon }_{\omega }^{{\prime} }$ and ${\varepsilon }_{V}^{{\prime} }$ represent corresponding nonlinear effects and are included in the total residual term.

Second, an empirical binning method using daily 500-hPa vertical velocity (*ω*_500_) as the dynamic proxy. Following refs. ^47,64^, for each grid point, we divide daily *ω*_500_ across boreal winter into bins with a uniform width (we estimated bins using widths of 10 hPa day^−1^, 20 hPa day^−1^ and 50 hPa day^−1^, results are similar). By estimating the relative frequency of occurrence for each bin, we obtain a probability density function of *ω*_500_ (PDF_*ω*_). For each bin, we then get an expected precipitation associated with same-day *ω*_500_ (averaged precipitation for each bin). In this way, we obtain a function (or relationship, PRE_*ω*_) between daily precipitation and binning *ω*_500_. The change in precipitation can then be expressed as:

12 $${P}^{{\prime} }={\int }_{-\infty }^{\infty }\bar{{\mathrm{PRE}}_{\omega }}\delta {\mathrm{PDF}}_{\omega }{d}_{\omega }+{\int }_{-\infty }^{\infty }\bar{{\mathrm{PDF}}_{\omega }}\delta {\mathrm{PRE}}_{\omega }{d}_{\omega }+{\int }_{-\infty }^{\infty }\delta {\mathrm{PRE}}_{\omega }\delta {\mathrm{PDF}}_{\omega }{d}_{\omega }$$

in which the first term of equation (12) denotes the dynamic component, showing the change in precipitation owing to a change in the PDF_*ω*_. This is estimated by the difference between climatology (1950–2022) $\bar{{{\rm{PDF}}}_{\omega }}$ and single-year PDF_*ω*_ (each year during 1950–2022). To test the sensitivity, we also estimate the difference using two and three moving years relative to the climatology and find similar results. The second term denotes the thermodynamic component, showing the change in precipitation owing to a change in the PRE_*ω*_. The third term denotes the nonlinear term. Trends for each term are subsequently estimated in comparison with our SLP-based statistical learning approach.

### Data analysis

Precipitation, SLP, zonal winds, specific humidity and evapotranspiration anomalies are estimated by subtracting the long-term monthly means (1950–2022) from each corresponding calendar month and then aggregated into boreal winter (DJF) anomalies. For MERRA-2, anomalies are based on the 1980–2022 climatology. Linear trends are estimated using least-squares regression and evaluated for significance at the 0.05 level. Density curves^65^ are constructed using precipitation trends at each grid pixel within the three selected regions. All area-based analysis is calculated by considering weights in different latitudes.

The ‘best-matching simulation’ is identified by the highest Pearson correlation between the total and dynamic precipitation trends from the CMIP6 and CESM2 ensembles and the corresponding components derived from ERA5 reanalysis across mid-to-high latitudes of the Northern Hemisphere. We find that the FGOALS-f3 model output and the 72nd member of the CESM2-LE yield the strongest correlation with ERA5 (for both total and dynamic precipitation trends but not the thermodynamic precipitation trends). Note that the simulation that best reproduces dynamic precipitation trends does not exhibit the highest correlations in SLP or U500. This probably reflects regional heterogeneity in the relationship between circulation and precipitation. Large-scale circulation patterns (SLP and U500) exert spatially varying controls on precipitation, being more influential in some regions than others. Meanwhile, the fidelity of simulated circulation trends relative to reanalysis also varies regionally. Consequently, regions in which circulation both strongly influences precipitation and is well reproduced by models do not necessarily align with those that dominate hemispheric-average metrics.

To assess the projected dynamic signals, we perform a signal-to-noise ratio analysis^17,50,66^. The signal is defined as the trend from the CMIP6 multimodel mean or CESM2 multimember mean. The noise is defined as the standard deviation of trends calculated from 150-year segments of pre-industrial simulations from 29 CMIP6 models, matching the length of the historical record and SSP370 (1950–2099). This yields a noise estimate based on 73-year trends across a total of 4,350 simulated years.

## Online content

Any methods, additional references, Nature Portfolio reporting summaries, source data, extended data, supplementary information, acknowledgements, peer review information; details of author contributions and competing interests; and statements of data and code availability are available at https://doi.org/10.1038/s41586-026-10474-y.

## Supplementary information

Supplementary Information

Peer Review file

## Figures and Tables

**Extended Data Table 1:**
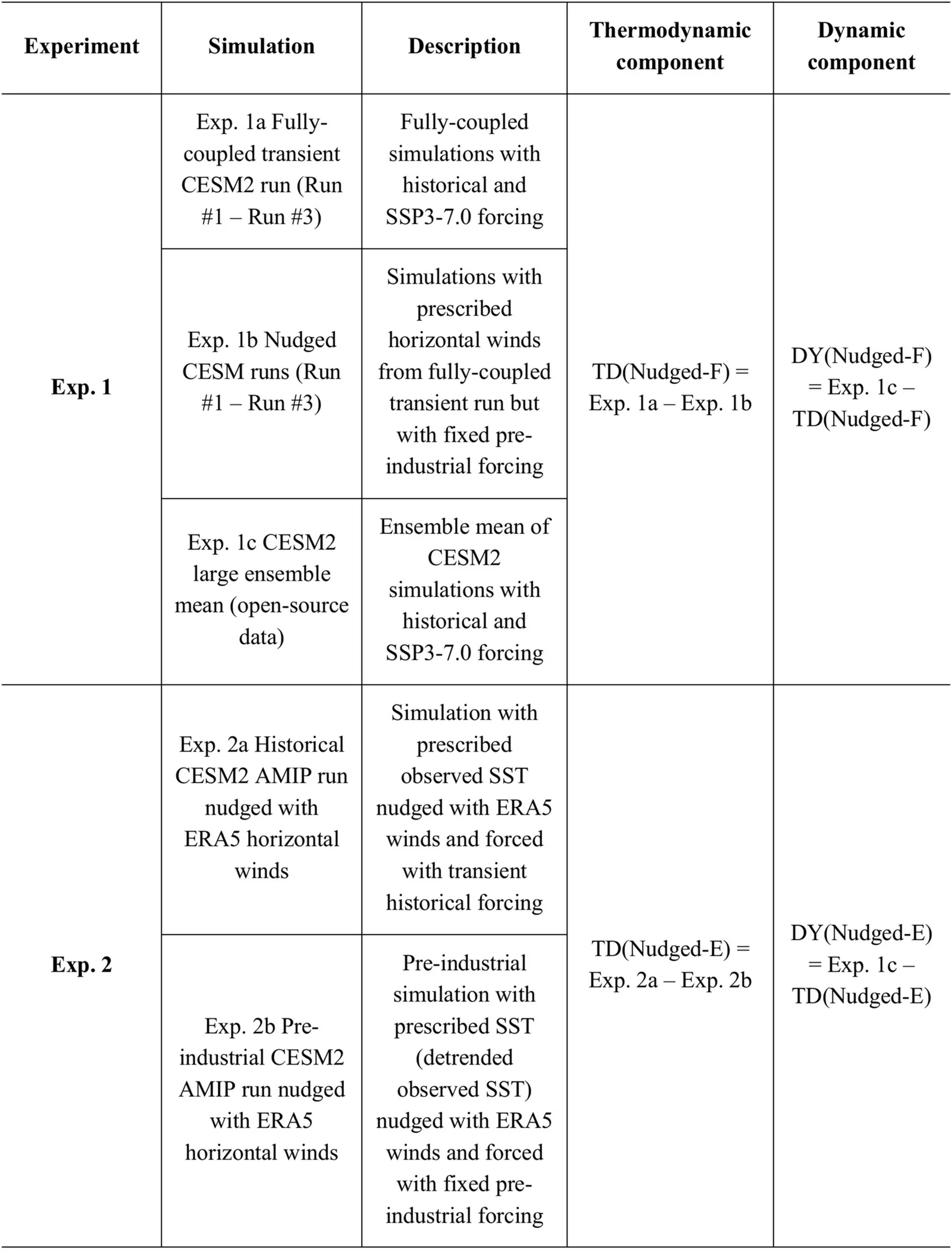
Experimental design and component decomposition of precipitation

## Data Availability

The ERA5 reanalysis data are from https://www.ecmwf.int/en/forecasts/datasets/reanalysis-datasets/era5. The MSWEP precipitation data are from https://www.gloh2o.org/mswep/. The JRA-3Q reanalysis data are from https://gdex.ucar.edu/datasets/d640000/dataaccess/#. The CRU precipitation data are from https://crudata.uea.ac.uk/cru/data/hrg/index.htm#current. The GPCC precipitation data are from https://www.dwd.de/EN/ourservices/gpcc/gpcc.html. The NCEP–NCAR reanalysis sea-level pressure data are from https://psl.noaa.gov/data/gridded/data.ncep.reanalysis.html. The NAO index data are from https://crudata.uea.ac.uk/cru/data/nao/nao.dat. The MERRA-2 dataset is from https://doi.org/10.5067/RKPHT8KC1Y1T. The CESM2-LE data are from https://www.cesm.ucar.edu/community-projects/lens2/data-sets. The CMIP6 data are from https://esgf-node.ipsl.upmc.fr/search/cmip6-ipsl/. The nudged CESM2 simulations used for the analysis are available from https://doi.org/10.5281/zenodo.19150219 (ref. ^67^).

## Code availability

The R (version 4.2.2) code used for data analysis is available at the repository in an Open Science Framework repository (https://osf.io/7xjma/?view_only=cff2af1a670b48749f09160f0b3492c4). All of the figures are generated using R (version 4.2.2). All base maps are produced using open-source coastline data (coastsCoarse) from the R package ‘fields’.
