# Supplementary material for: Uncertain dynamic response of mid-latitude winter precipitation
Source: Nature. 2026 Apr 29;653(8113):110–6. doi: 10.1038/s41586-026-10474-y (PMC13675132; doi:10.1038/s41586-026-10474-y)
Supplement: Supplementary file 1 [file 41586_2026_10474_MOESM1_ESM.pdf]

nature portfolio

https://doi.org/10.1038/s41586-026-10474-y

Supplementary information

Uncertain dynamic response of mid-latitude winter precipitation

In the format provided by the authors and unedited

Supplementary Material for

Uncertain dynamic response of mid-latitude winter precipitation

Lei Gu[1*], Dominik L. Schumacher[1], Sebastian Sippel[2], Erich M. Fischer[1], Istvan Dunkl[2], Robin Noyelle[1], Jitendra Singh[1], Lorenzo Pierini[1], Reto Knutti[1]

[1]Institute for Atmospheric and Climate Science, ETH Zurich, Zurich, Switzerland

[2]Leipzig Institute for Meteorology, Leipzig University, Leipzig, Germany

*Correspondence: L. Gu (lei.gu@physics.ox.ac.uk)

Contents of this Supplementary Information:

Overview of supplementary contents

Supplementary Figures 1 to 17

Summary about supplementary information

This supplementary file includes 17 figures.

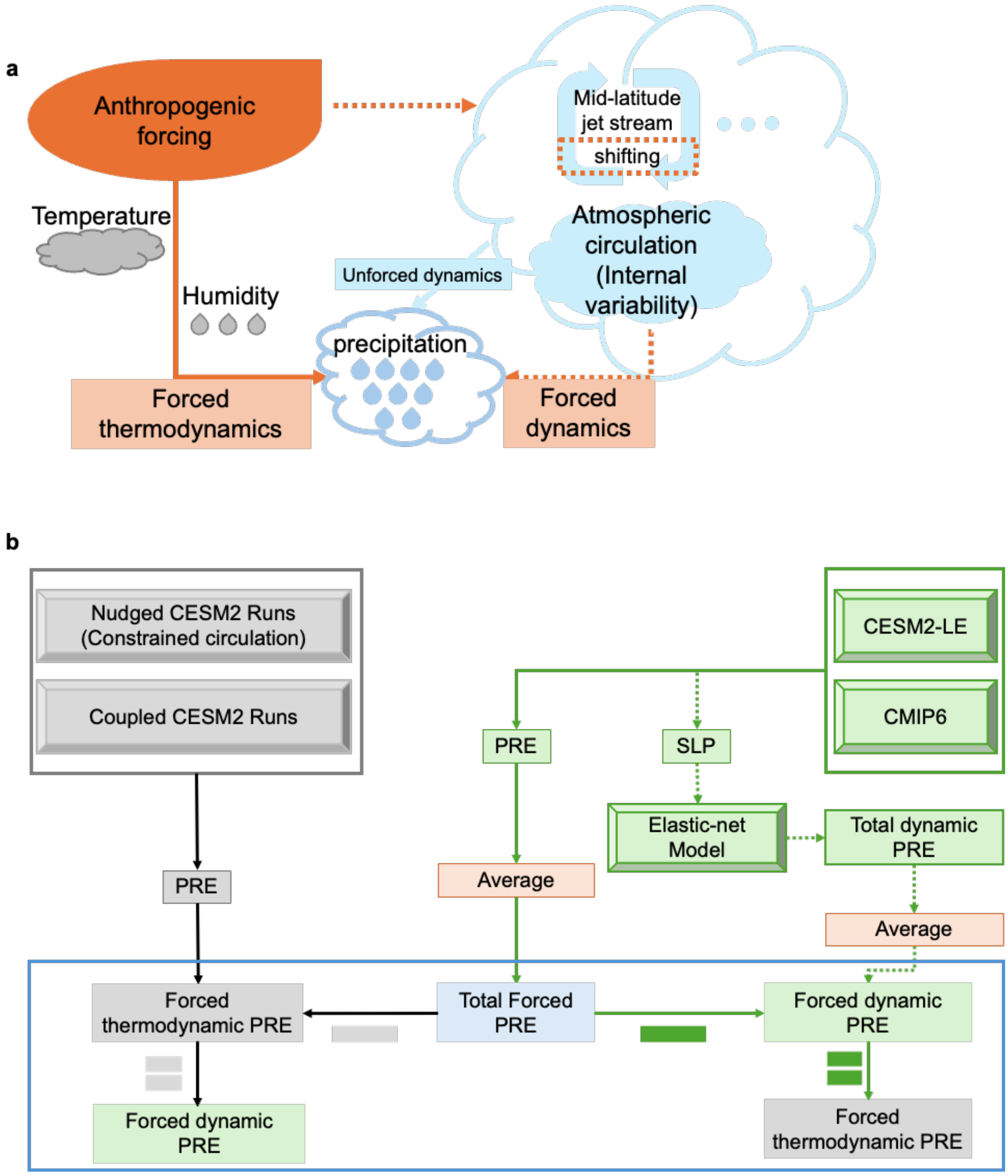

Supplementary Figure 1. The schematic figure of precipitation components and the framework to quantify their respective contributions. a, The schematic conception of forced thermodynamic, forced dynamic, and unforced dynamic contribution to precipitation changes. **b**, The framework we proposed to systematically decompose precipitation changes into various components. '*PRE*' denotes precipitation and '*SLP*' denotes sea level pressure. The statistical learning approach – elastic-net model enables estimation of dynamic components and then isolation of forced dynamic precipitation change from total forced change. Conversely, the nudged CESM2 model experiments enable ideal estimation of forced thermodynamic precipitation change, and then leave the residual as circulation-induced precipitation trends.

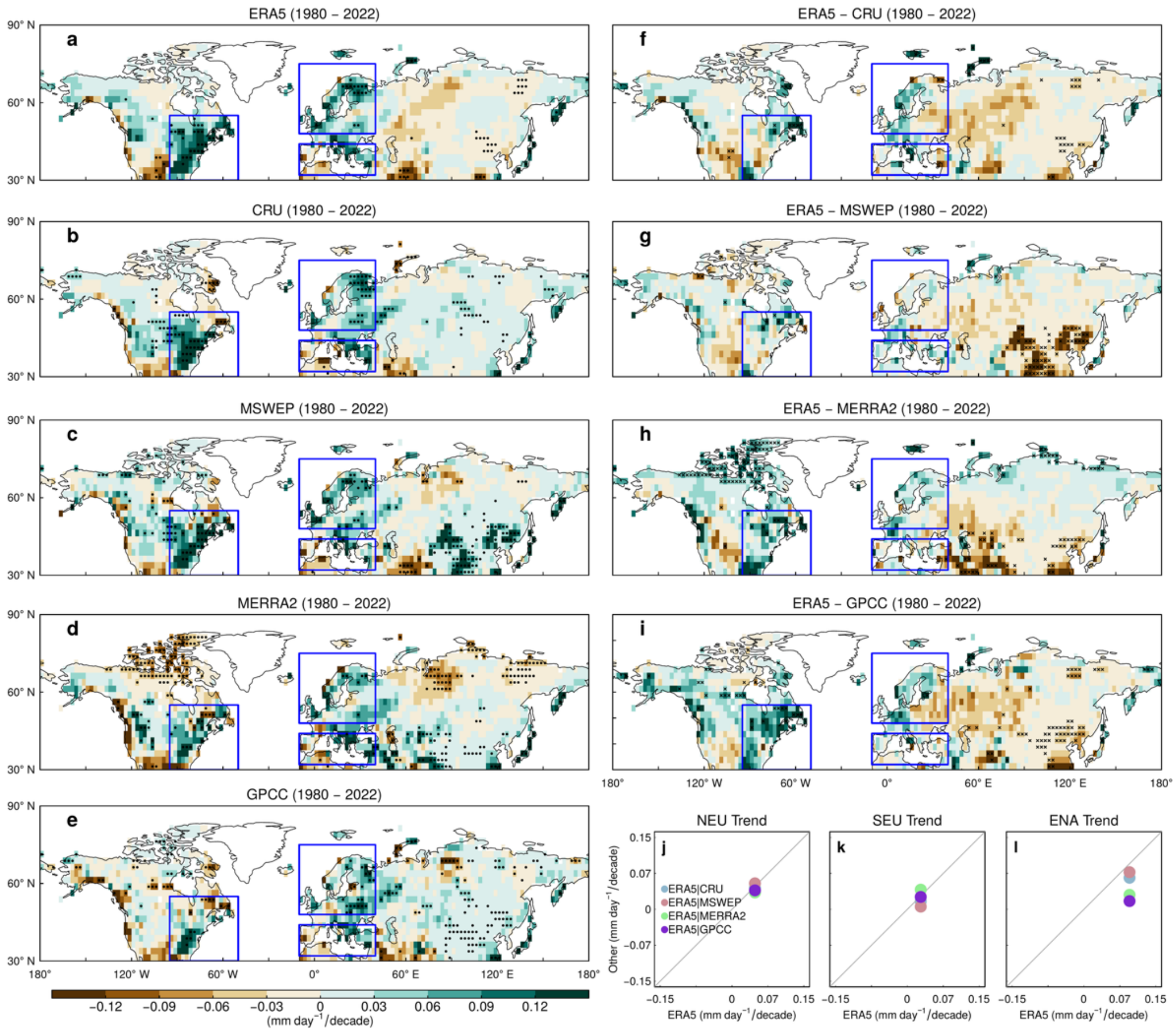

Supplementary Figure 2. The observation-based winter precipitation trends during 1980–2022. a-e, The observation-based winter precipitation trends estimated by the ERA5 (**a**), CRU (**b**), MSWEP (**c**), MERRA2 (**d**), GPCC (**e**) datasets. **f-i**, The difference maps between ERA5 and CRU (**f**), ERA5 and MSWEP (**g**), ERA5 and MERRA2 (**h**), ERA5 and GPCC (**i**). The dot stippling in **a-e** represents significant trends at the 0.05 level (p-value < 0.05). The crosses in **f-i** mark the trends estimated by the ERA5 significantly differ from trends estimated by other datasets (t-test, p-value < 0.05). **j-l**, Scatter plots showing winter precipitation trends in ERA5 versus other observational datasets (CRU, MSWEP, MERRA-2, and GPCC) over northern Europe (**j**, NEU), southern Europe (**k**, SEU), and eastern North America (**l**, ENA).

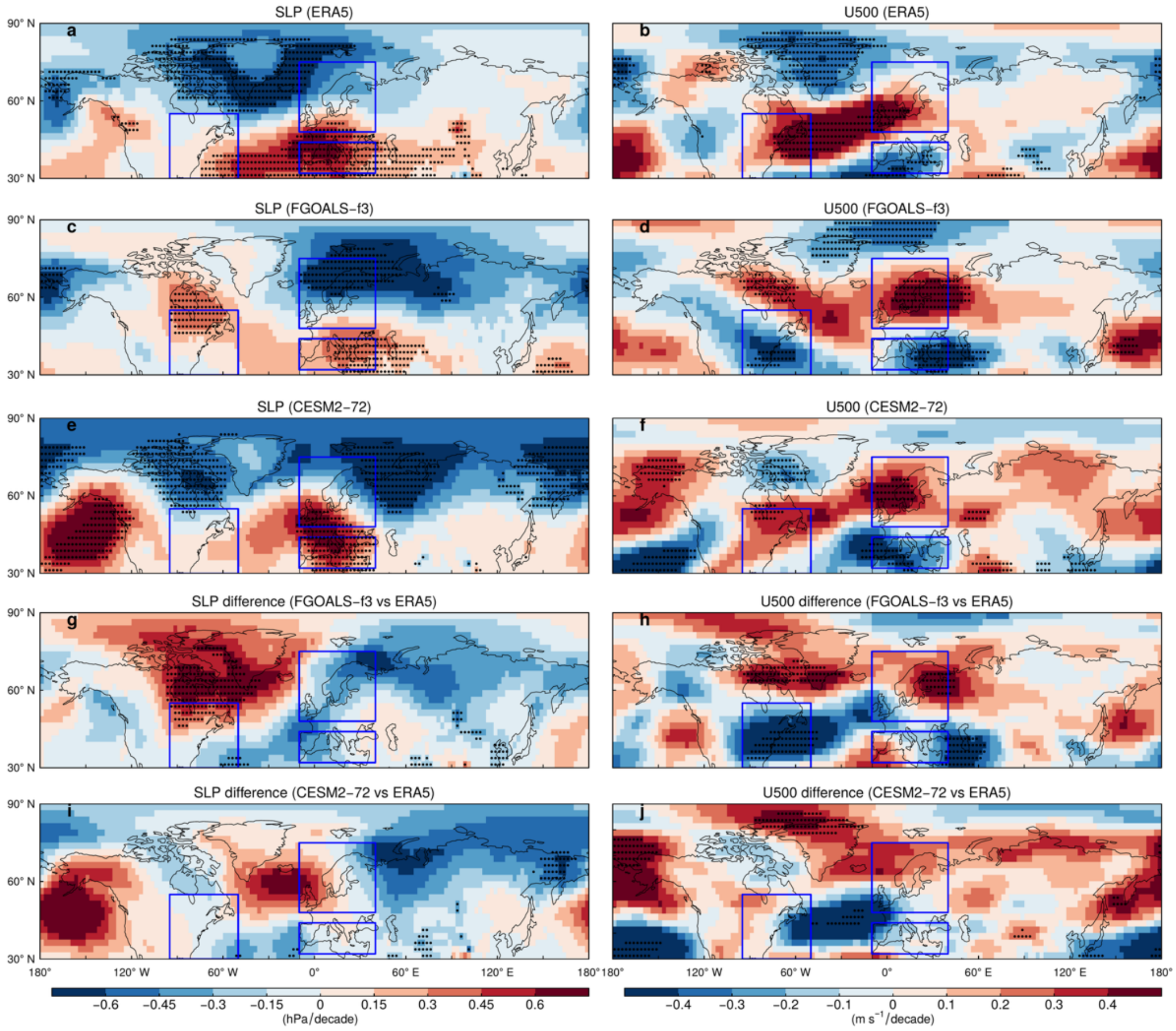

Supplementary Figure 3. Trends in sea level pressure and zonal wind by reanalysis and climate models. a,**b**, Spatial maps of trends in winter sea level pressure (*SLP*, **a**) and zonal wind at 500hPa (*U500*, **b**) over 1950–2022 from the ERA5 reanalysis. **c**,**d**, Spatial maps of trends in winter *SLP* (**c**) and *U500* (**d**) over 1950–2022 estimated by "the best-matching simulation" in CMIP6 ensemble. **e**,**f**, The same as **c**,**d**, but by "the best-matching simulation" in CESM2 large ensemble. **g**,**h**, The differences of trends in winter SLP trends (**g**) and U500 trends (**h**) between best-matching simulation in CMIP6 and ERA5. **i**,**j**, The same as **g**,**h**, but differences between best-matching simulation in CESM2-LE and ERA5. The stippling indicates the trend is significant at the 0.05 level (p-value < 0.05).

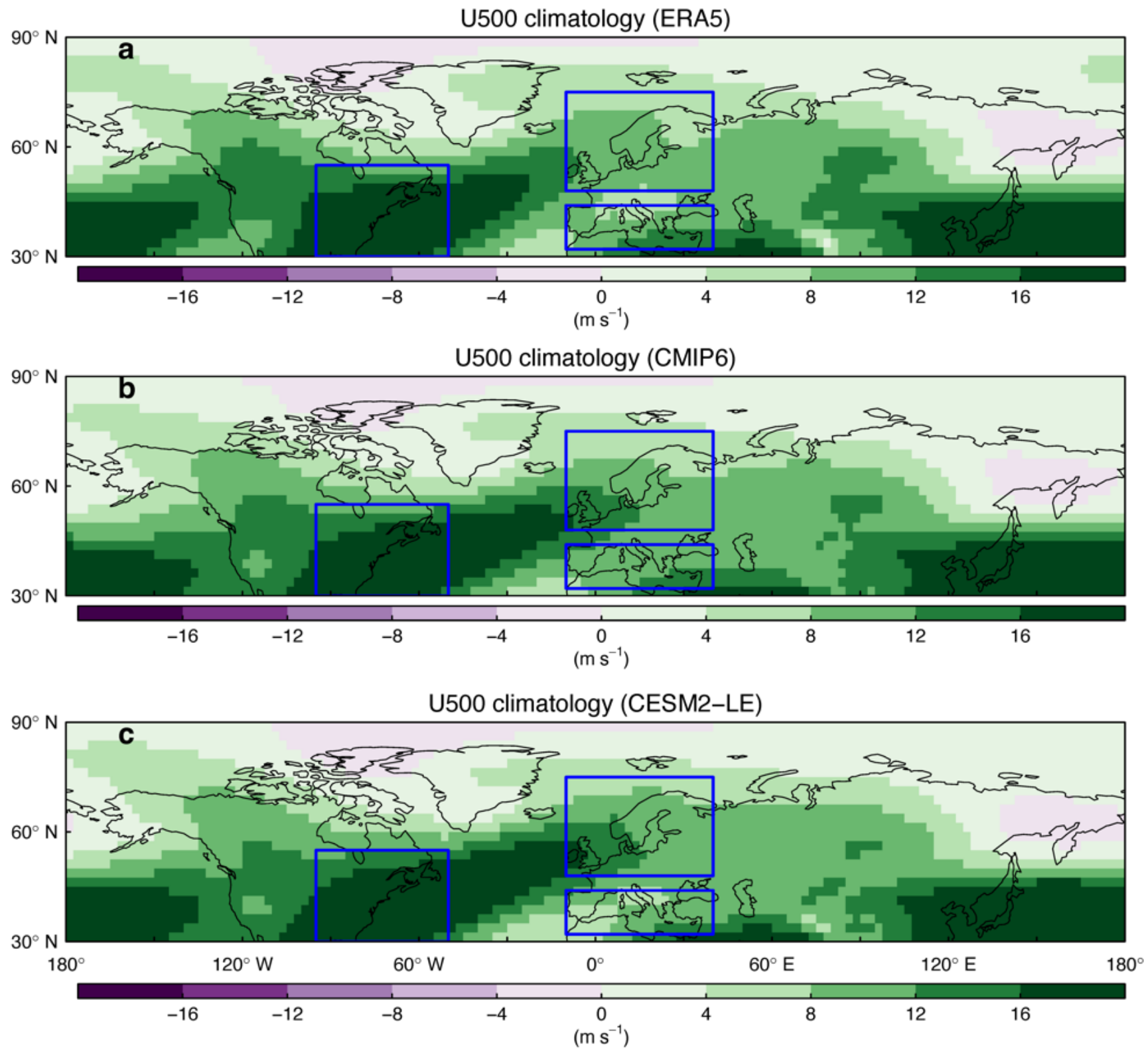

Supplementary Figure 4. The climatology of zonal wind across mid-high latitudes of the Northern Hemisphere from the ERA5 and climate model ensembles. a, The climatology of zonal wind at 500hPa between 1950 and 2022 from the ERA5 reanalysis dataset. **b**, The climatology of zonal wind at 500hPa between 1950 and 2022 based on multi-model mean of CMIP6 ensemble. **c**, The climatology of zonal wind at 500hPa between 1950 and 2022 based on multi-member mean of CESM2 large ensemble.

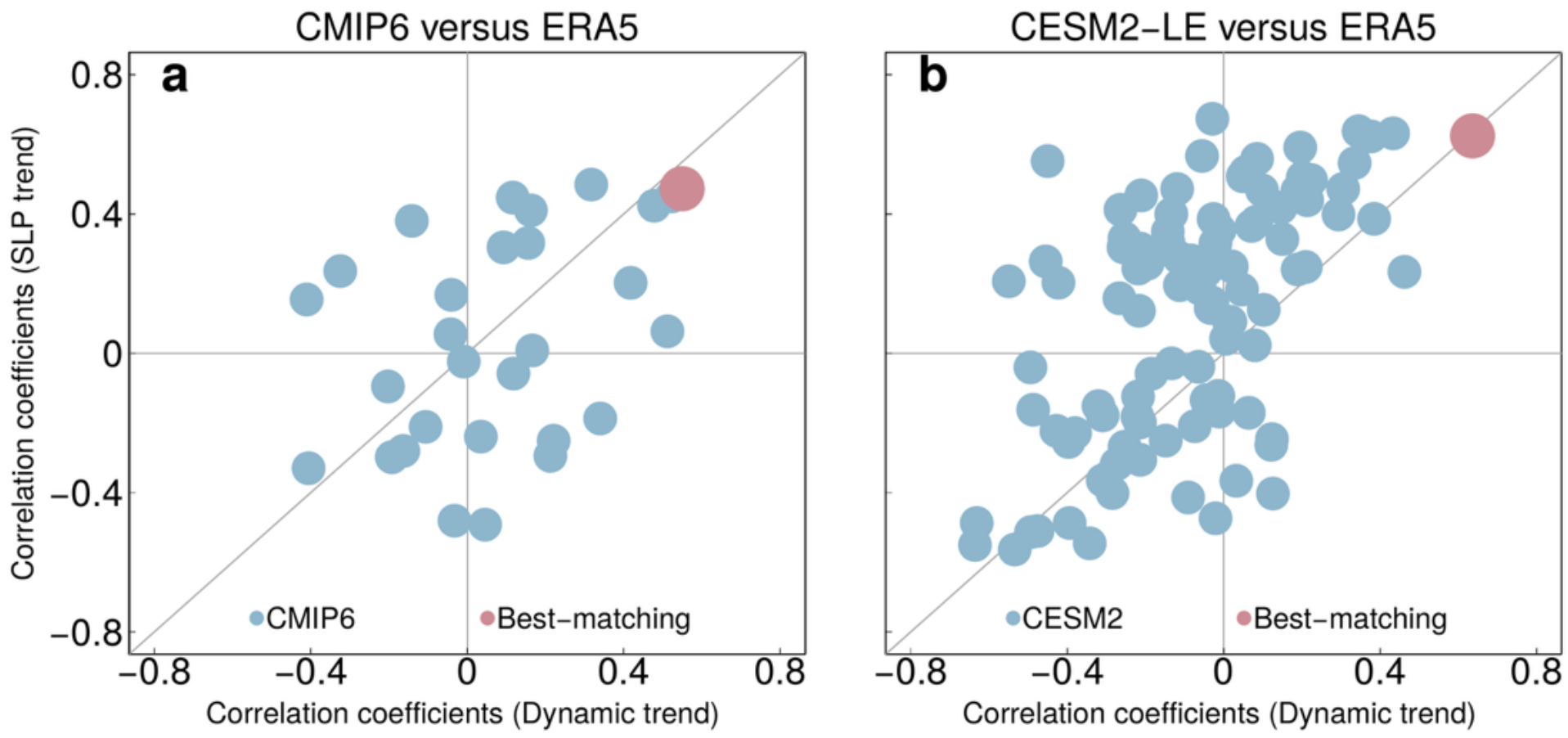

Supplementary Figure 5. The Pearson correlation coefficients of dynamic precipitation trend and dynamic indicator (sea level pressure) trend between climate model simulations and the ERA5 reanalysis. **a**, The correlation coefficients between the CMIP6 ensemble simulations and the ERA5 reanalysis. *x*-axis denotes the correlation of dynamic precipitation trend; *y*-axis denotes the correlation of sea level pressure (*SLP*) trend. **b**, The same as **a**, but correlation coefficients between the CESM2-LE and the ERA5.

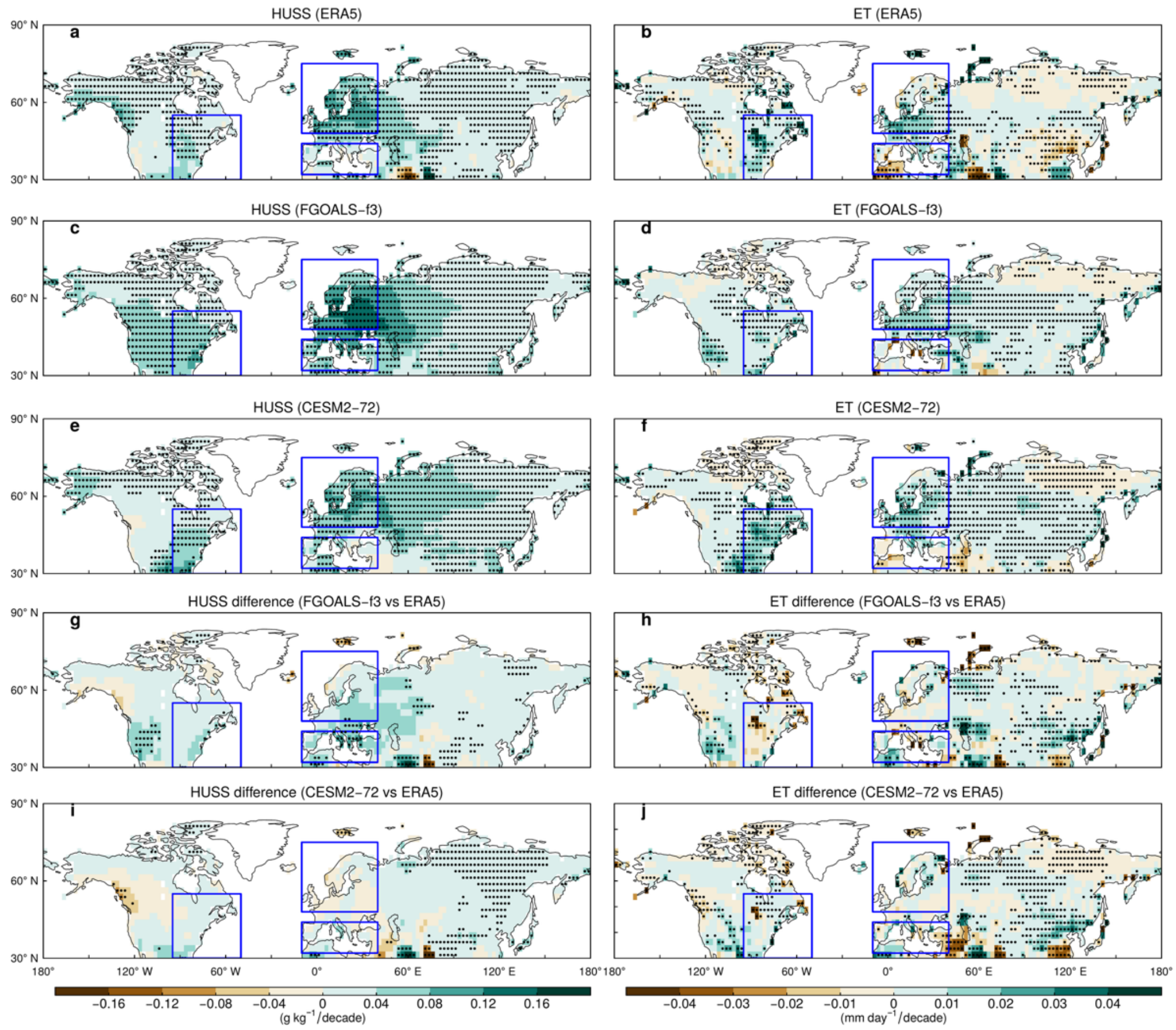

Supplementary Figure 6. Trends in specific humidity and evapotranspiration by reanalysis and climate models. a,**b**, Spatial maps of trends in winter specific humidity (*HUSS*, **a**) and evapotranspiration (*ET*, **b**) over 1950–2022 from the ERA5 reanalysis. **c**,**d**, Spatial patterns of trends in winter *HUSS* (**c**) and *ET* (**d**) over 1950–2022 estimated by "the best matching simulation" in CMIP6 ensemble. **e**,**f**, The same as **c**,**d**, but by "the best matching simulation" in CESM2 large ensemble. **g**,**h**, The differences of trends in specific humidity trends (**g**) and evapotranspiration trends (**h**) between best-matching simulation in CMIP6 and ERA5. **i**,**j**, The same as **g**,**h**, but differences between best-matching simulation in CESM2-LE and ERA5.The stippling indicates the trend is significant at the 0.05 level (p-value < 0.05).

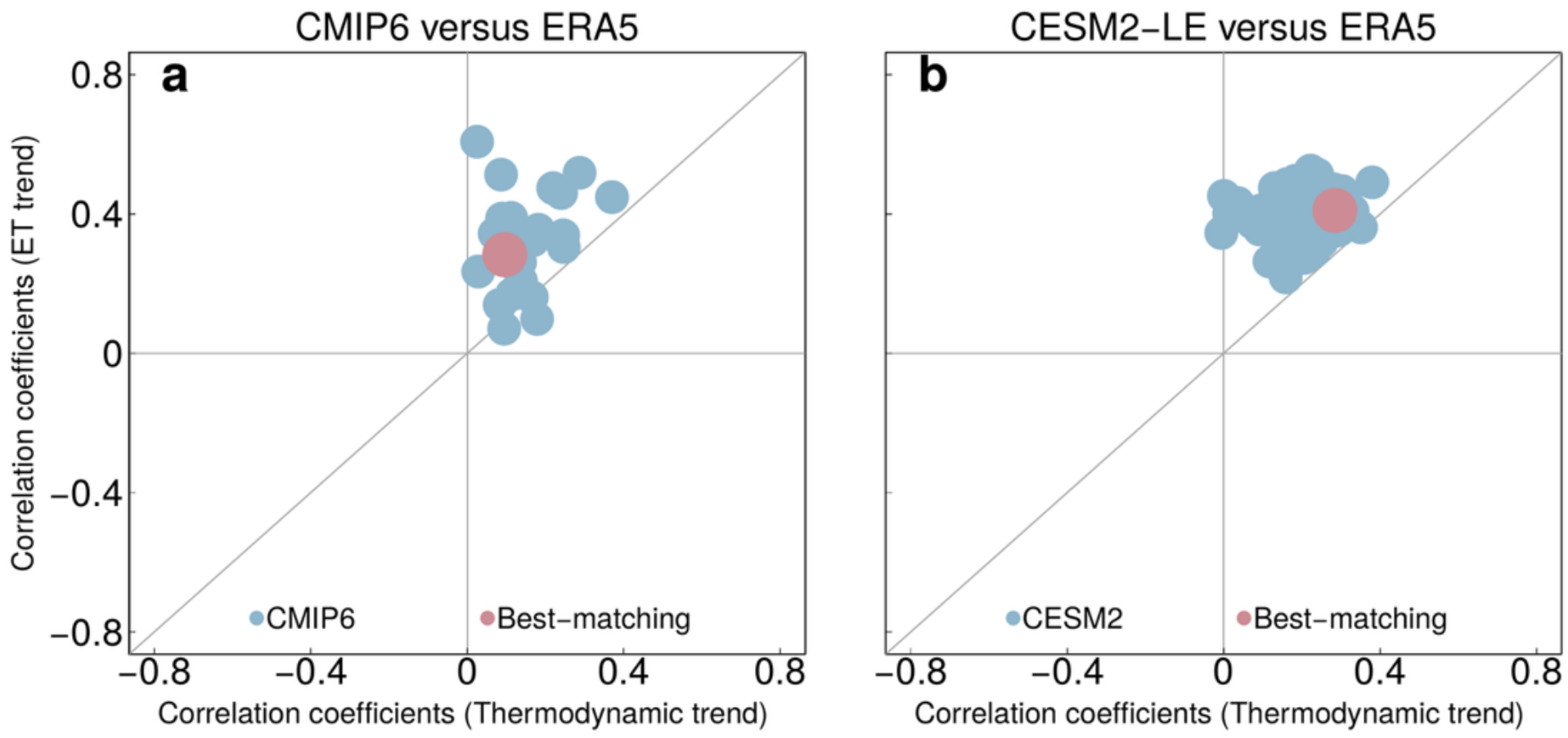

Supplementary Figure 7. The Pearson correlation coefficients of thermodynamic precipitation trend and thermodynamic indicator (evapotranspiration) trend between climate model simulations and the ERA5 reanalysis. a, The correlation coefficients between the CMIP6 ensemble simulations and the ERA5 reanalysis. *x*-axis denotes the correlation of dynamic precipitation trend; *y*-axis denotes the correlation of evaporation (ET) trend. **b**, The same as **a**, but correlation coefficients between the CESM2-LE and the ERA5.

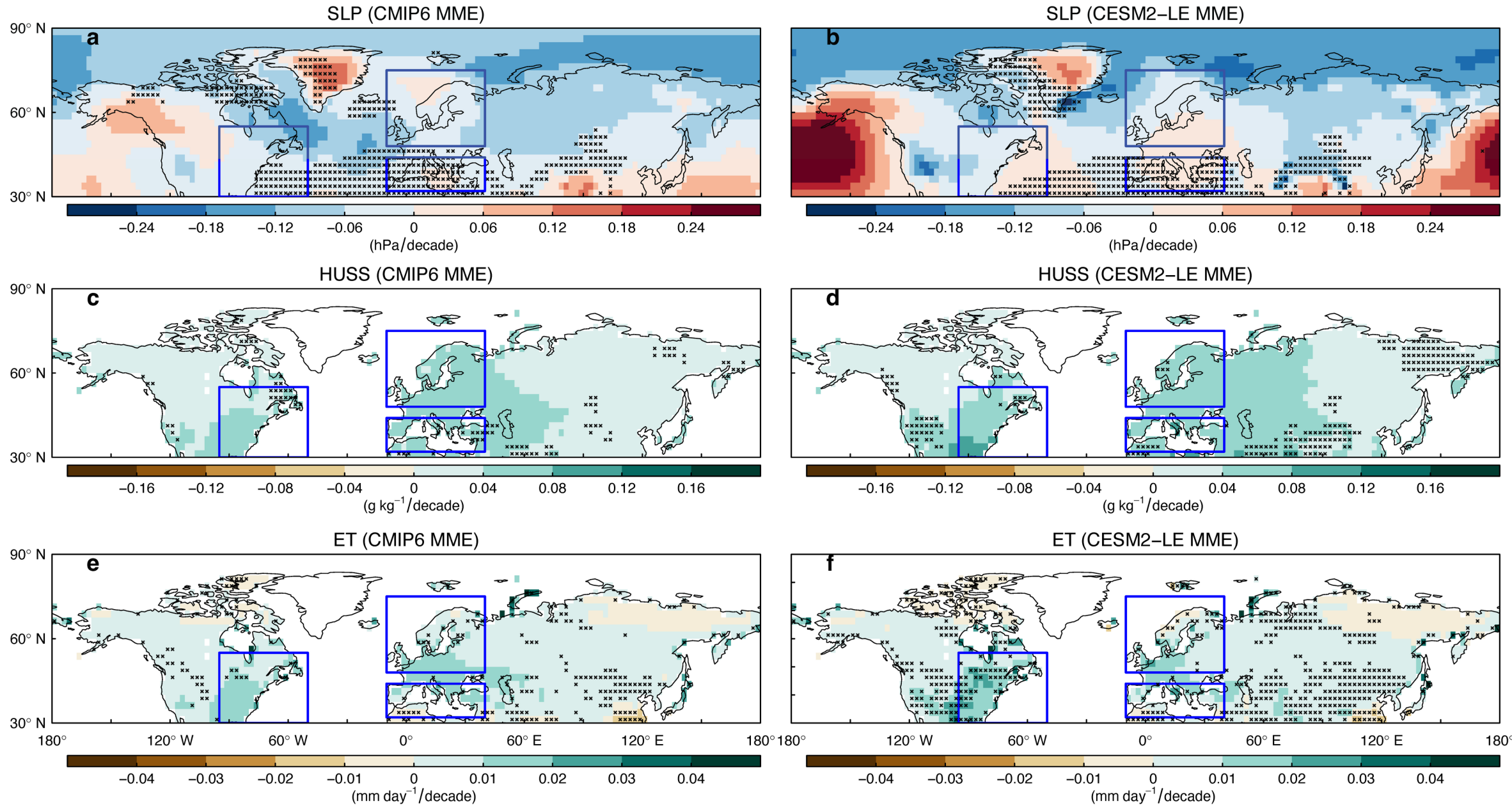

Supplementary Figure 8. Anthropogenic forced trends in sea level pressure, specific humidity, and evapotranspiration simulated by climate models. a,**b**, Spatial maps of anthropogenic forced trends in winter sea level pressure (*SLP*) over 1950–2022 estimated by averaging multi-model ensemble of CMIP6 (**a**) and multi-member ensemble of CESM2 (**b**). **c**,**d**, Spatial maps of anthropogenic forced trends in winter specific humidity (*HUSS*) over 1950–2022 estimated by averaging multi-model ensemble of CMIP6 (**c**) and multi-member ensemble of CESM2 (**d**). **e**,**f**, Spatial maps of anthropogenic forced trends in winter evapotranspiration (*ET*) over 1950–2022 estimated by averaging multi-model ensemble of CMIP6 (**e**) and multi-member ensemble of CESM2 (**f**). The cross-stippling indicates areas where the *SLP* (**a**,**b**), *HUSS* (**c**,**d**,), and *ET* (**e**,**f**) trends from the ERA5 locates outside the range of CMIP6 or CESM2-LE ensemble.

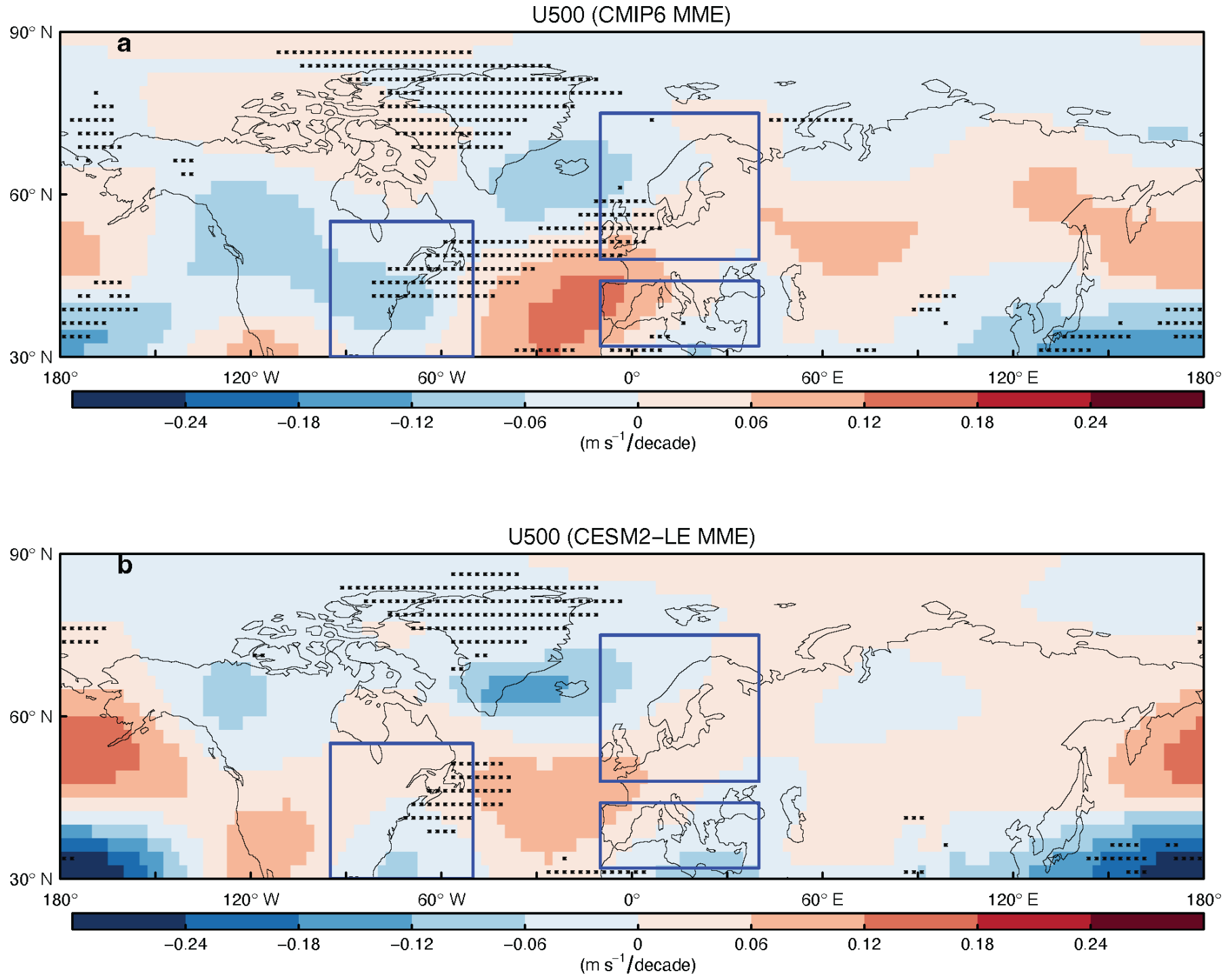

Supplementary Figure 9. Anthropogenic forced trends in zonal winds (U500) simulated by climate models. a,b, Spatial maps of anthropogenic forced trends in winter zonal winds (*U500*) over 1950–2022 estimated by averaging multi-model ensemble of CMIP6 (**a**) and multi-member ensemble of CESM2 (**b**). The cross-stippling indicates areas where the *U500* trend from the ERA5 locates outside the range of CMIP6 or CESM2-LE ensemble.

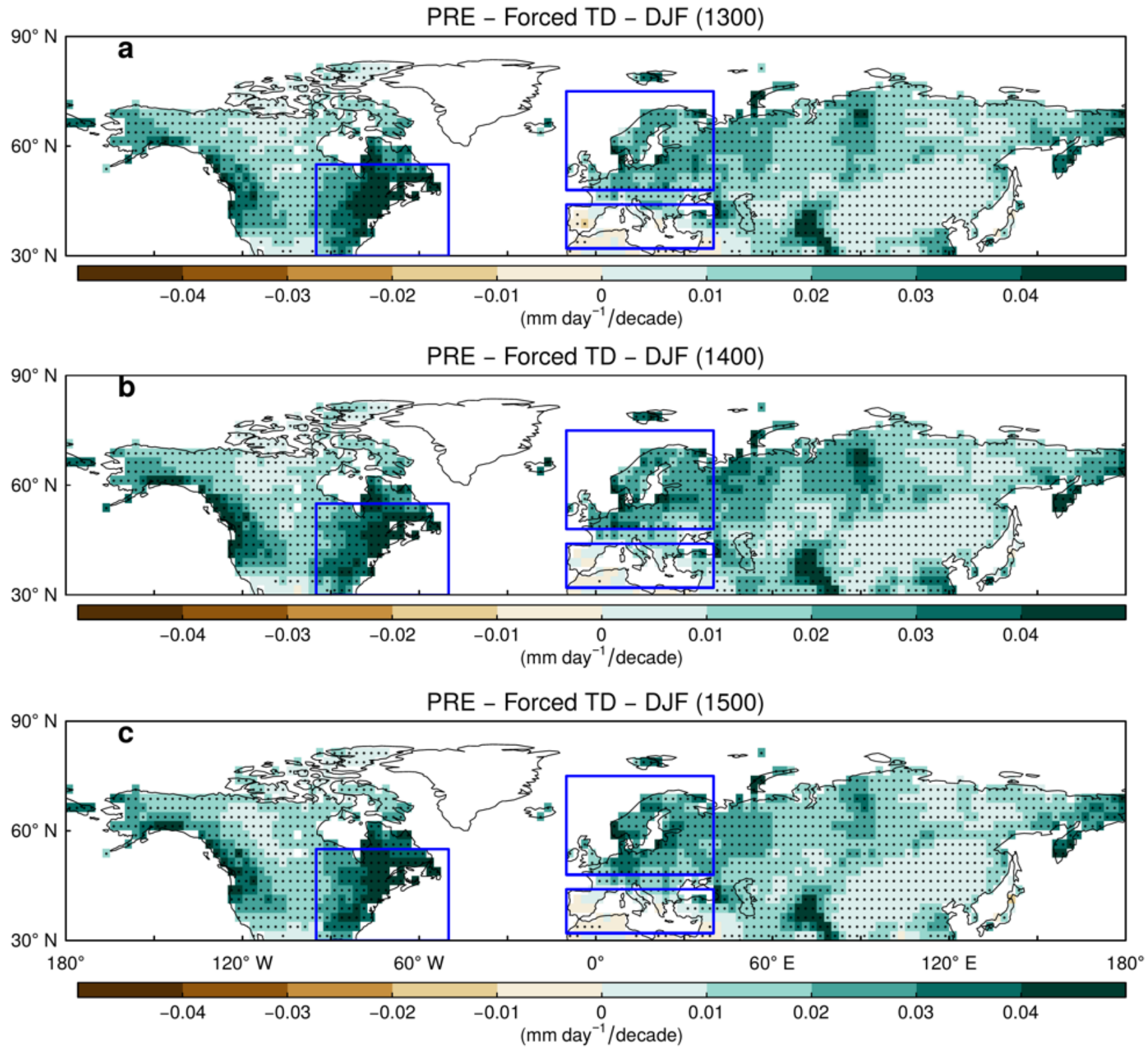

Supplementary Figure 10. The anthropogenic forced thermodynamic precipitation trends in boreal winter over the period 1950–2022 in nudged climate model experiments with run-1300, run-1400, run-1500. **a**, The forced thermodynamic precipitation trends based on nudging whole atmospheric winds with run-1300. **b**,**c**, The same as **a**, but with run-1400 and run-1500. The dot stippling represents significant trends at the 0.05 level (p-value < 0.05).

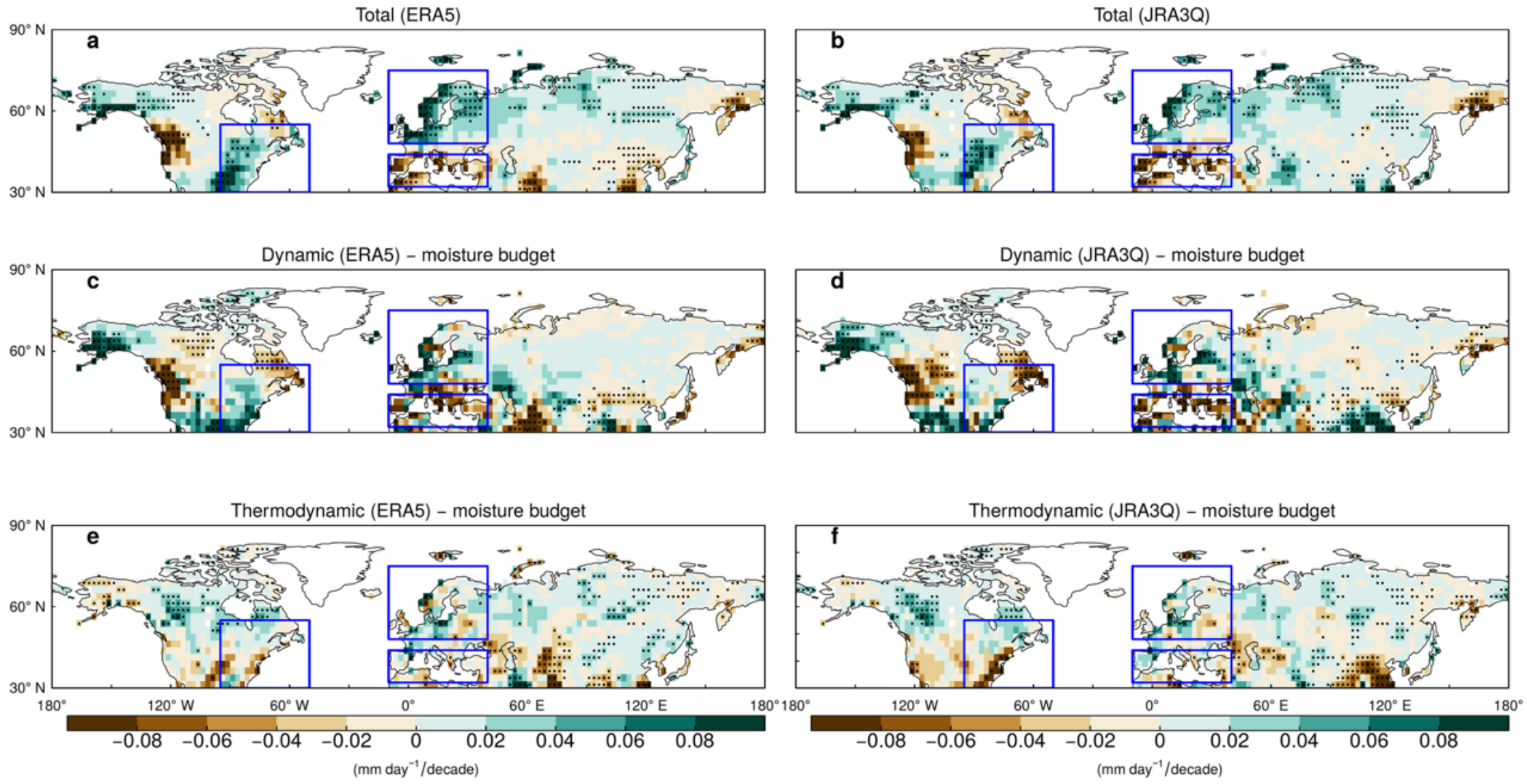

Supplementary Figure 11. Dynamic and thermodynamic winter precipitation trends based on moisture budget diagnostics. a,**c**,**e**, The total (**a**), dynamic (**c**), and thermodynamic (**e**) winter precipitation trends based on moisture budget diagnostics from the ERA5 reanalysis dataset. Note that the dynamic and thermodynamic trends include respective contributions from both horizontal and vertical moisture advection. **b**,**d**,**f**, The same as **a,c,e,** but from the JRA-3Q reanalysis dataset. The dot stippling represents significant trends at the 0.05 level (p-value < 0.5).

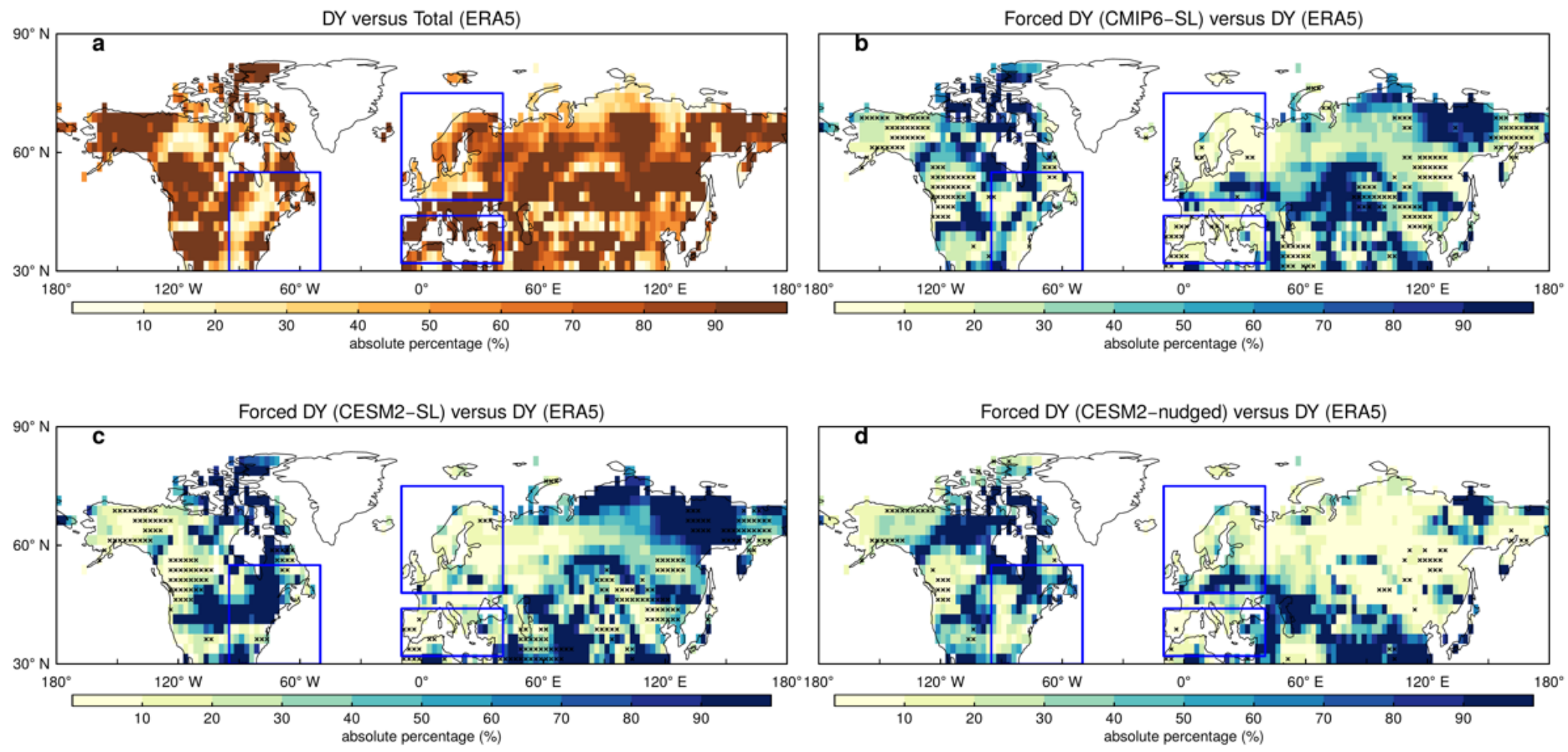

Supplementary Figure 12. Spatial maps of the dynamic contributions to precipitation trends during 1980–2022 period in reanalysis and model simulations. a, The absolute percentage of dynamic precipitation trends versus total precipitation trends in the ERA5. **b**, The absolute percentage of human forced dynamic precipitation trends (based on applying elastic-net regression models to the CMIP6 ensemble) versus ERA5-based dynamic trends. **c**, The same as **b**, but based on applying elastic-net regression models to the CESM2 large ensemble. **d**, The same as **b**, but based on nudged climate model experiments. The cross-stippling in **b-d** indicates areas where the dynamic precipitation trend from the ERA5 locates outside the range of total dynamic spreads in climate models.

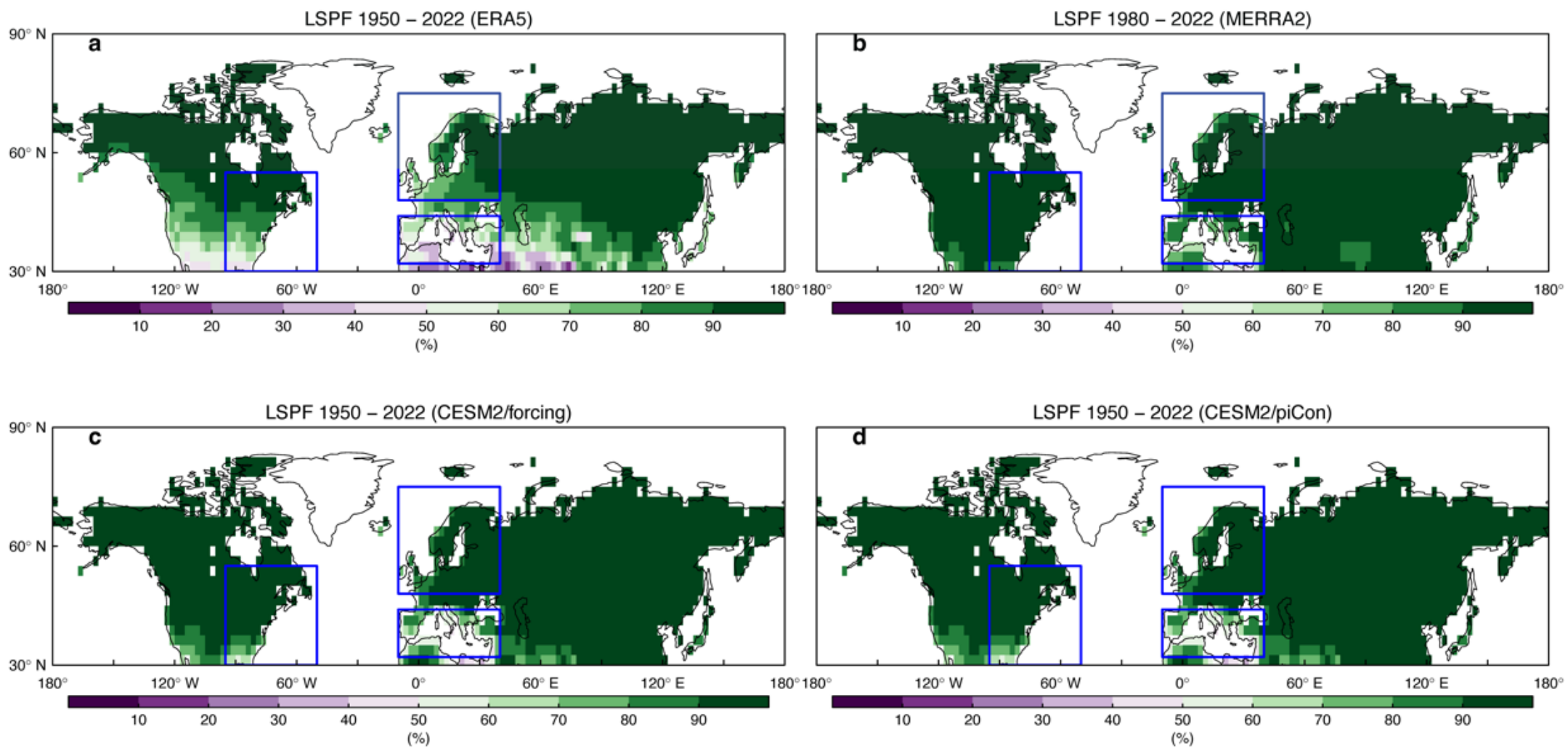

Supplementary Figure 13. Spatial maps of the fraction of large-scale precipitation relative to total precipitation in observations and model simulations. a,**b**, The fraction of large-scale precipitation relative to total precipitation over the 1950–2022 period estimated by the ERA5 dataset (**a**) and over the 1980–2022 period estimated by the MERRA-2 dataset (**b**). **c**,**d**, The fraction of large-scale precipitation relative to total precipitation over the 1950–2022 period estimated by coupled (**c**) CESM2 simulations (averaged over run-1300, run-1400, and run-1500) and nudged (**d**) CESM2 simulations (averaged over paired run-1300, run-1400, and run-1500 under the preindustrial scenario).

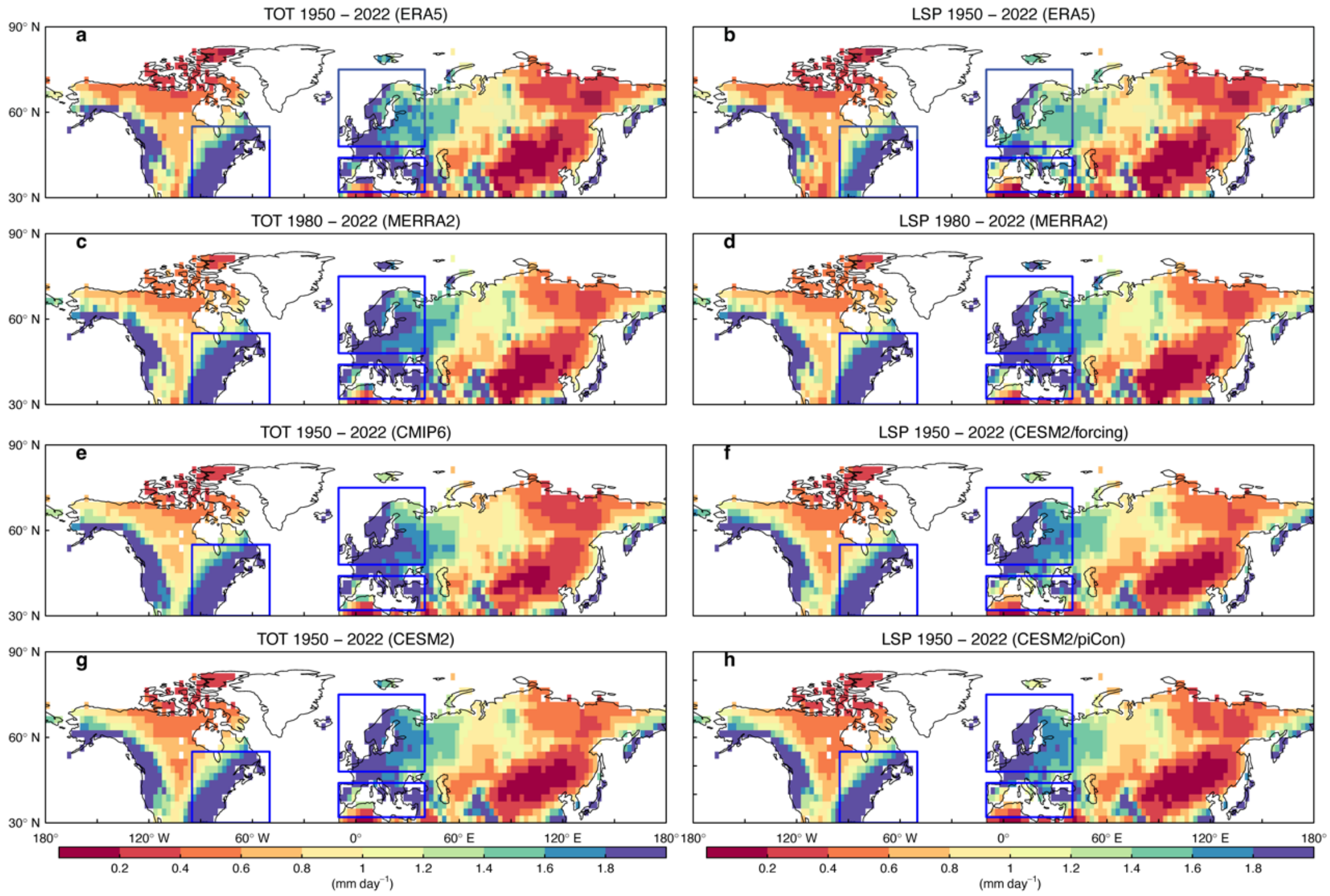

Supplementary Figure 14. The climatology of total winter precipitation and large-scale winter precipitation in reanalysis and model simulations. a,**c**,**e**,**g**, The climatology of total winter precipitation across mid-high latitudes of the Northern Hemisphere from the ERA5 (**a**), MERRA-2 (**c**), CMIP6 ensemble (**e**), CESM2 large ensemble (**g**). **b**,**d**,**f**,**h**, The climatology of large-scale winter precipitation across mid-high latitudes of the Northern Hemisphere from the ERA5 (**b**), MERRA-2 (**d**), CESM2 forcing simulations (**f**), and CESM2 pre-industrial (piCon) simulations (**h**).

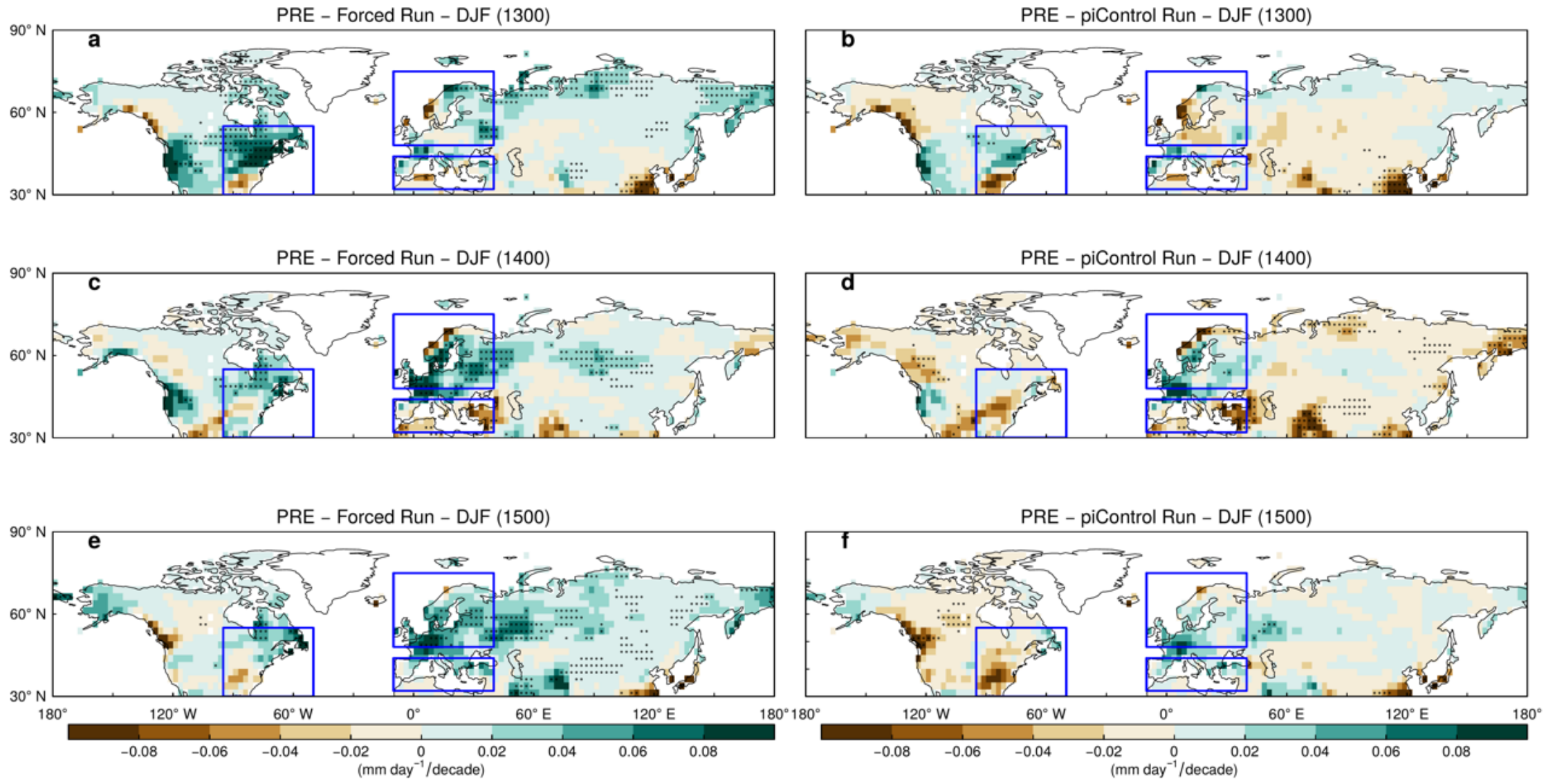

Supplementary Figure 15. Winter precipitation trends over the period 1950–2022 in nudged climate model experiments with run-1300, run-1400, run-1500. **a**,**b**, The winter precipitation trends based on the coupled CESM2 with run-1300 under historical and SSP370 scenarios (**a**) and the paired run-1300 under preindustrial scenario but the whole atmospheric horizontal winds are nudged to the coupled run-1300 (**b**). **c**,**d**, The same as **a**,**b**, but with coupled run-1400 and nudged run1400. **e**,**f**, The same as **a**,**b**, but with coupled run-1500 and nudged run1500. The dot stippling represents significant trends at the 0.05 level (*p*-value < 0.05).

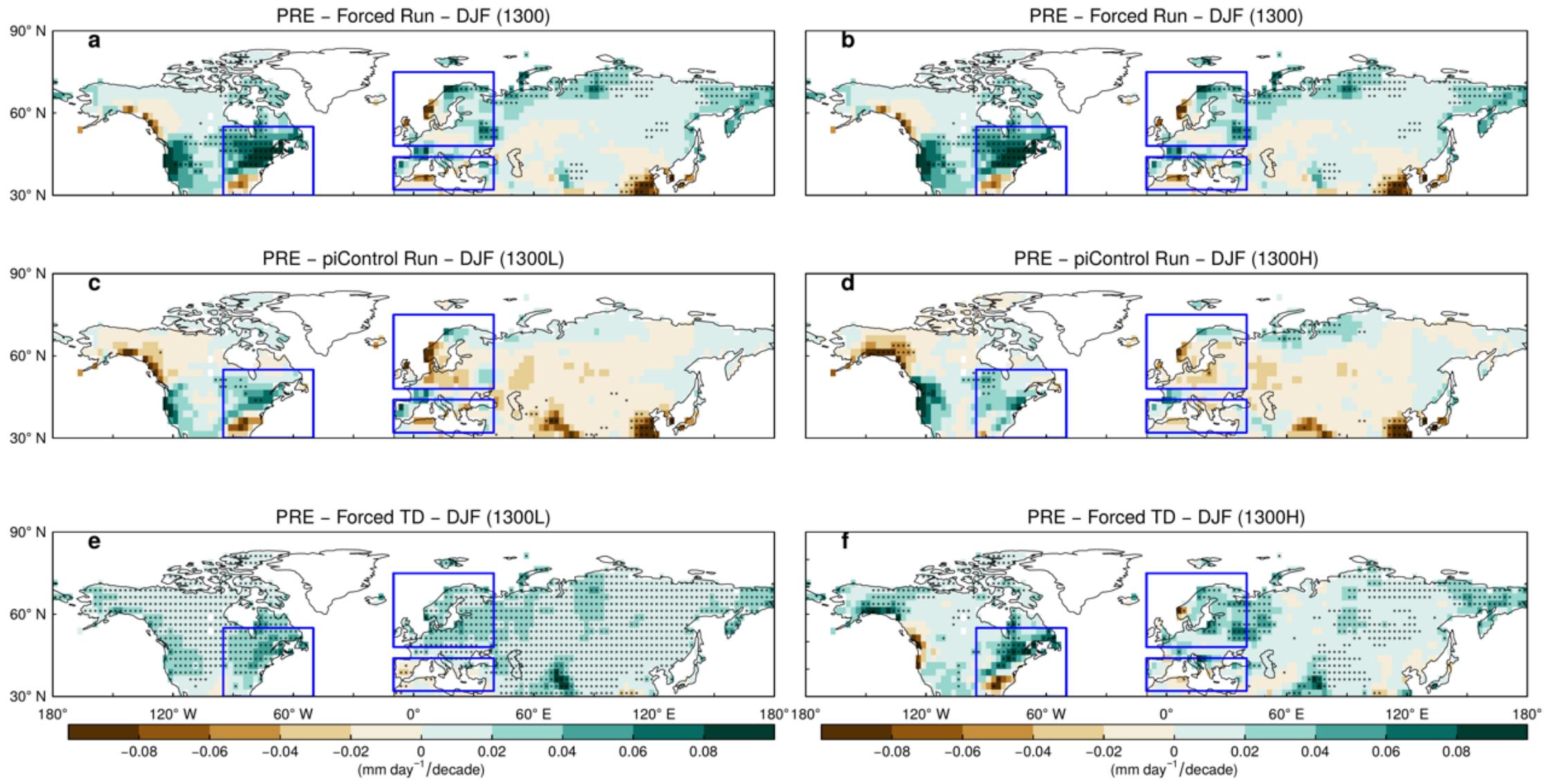

Supplementary Figure 16. Winter precipitation trends over the period 1950–2022 in nudged climate model experiments with run-1300. a,**b**, The winter precipitation trends based on the coupled CESM2 with run-1300 under historical and SSP370 scenarios. **c**,**d**, The paired run-1300 under preindustrial scenario but the horizontal winds above 691hPa (**c**) and above 322hPa (**d**) are nudged to the coupled run-1300. **e**,**f**, The forced thermodynamic precipitation trends based on the 691hPa experiment (**e**) and the 322hPa experiment (**f**). The dot stippling represents significant trends at the 0.05 level (p-value < 0.05)

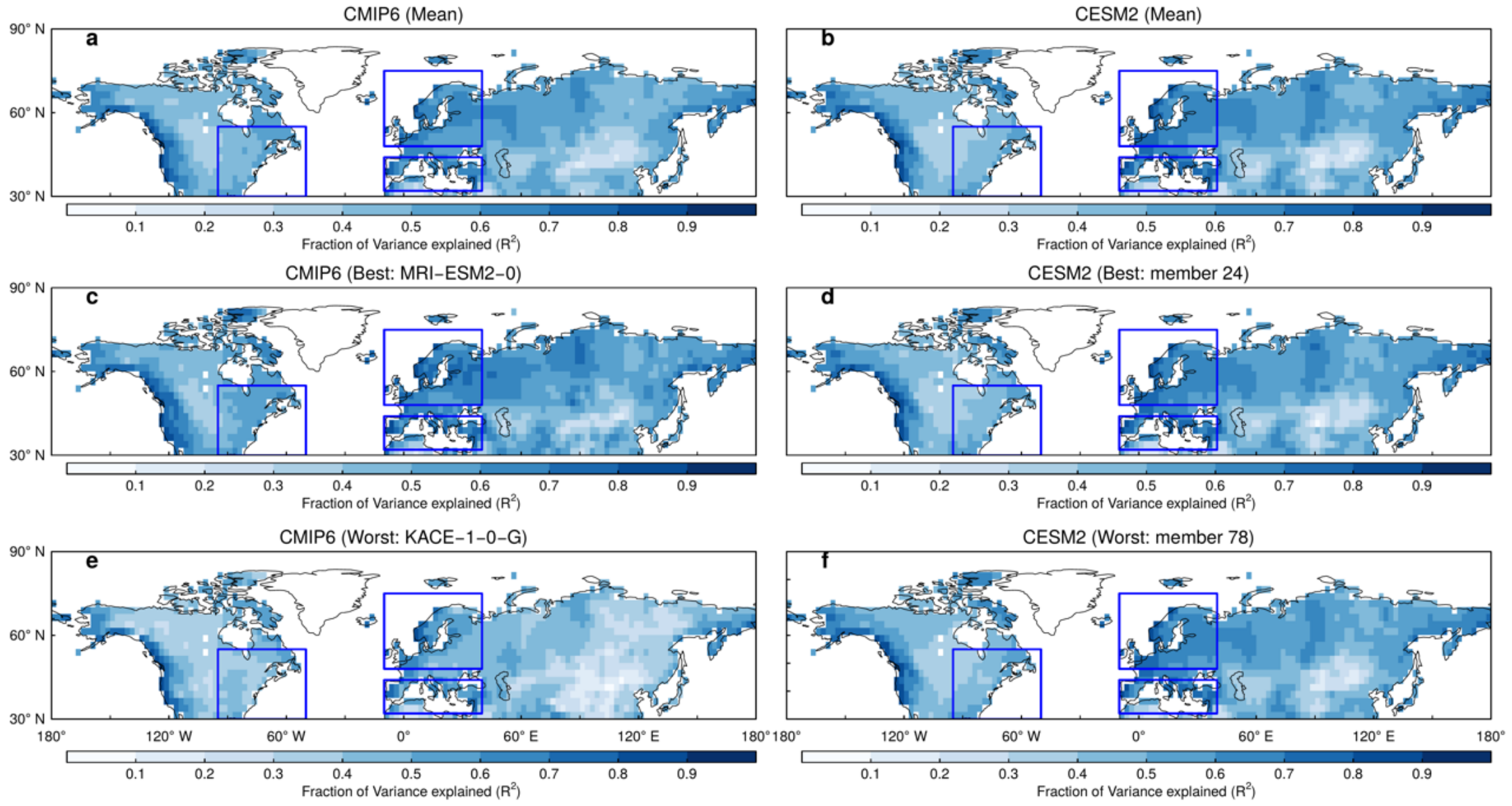

Supplementary Figure 17. Performance of winter precipitation predictions based on the statistical learning approach from the CMIP6 and CESM2 ensembles. a,**b**, The fraction of variance explained (R^2) of predicted winter (DJF) precipitation based on the CMIP6 (**a**) and CESM2 (**b**) ensemble mean during the 1950–2022 period. **c**,**d**, The same as **a**,**b**, but based on the best-performing model in CMIP6 ensemble (**c**) and best-performing member in CESM2 (**d**) ensemble with the highest R^2. **e**,**f**, The same as **a**,**b**, but based on the worst-performing model in CMIP6 ensemble (**e**) and worst-performing member in CESM2 (**f**) ensemble with the lowest R^2. The R^2 measures the variability in winter precipitation explained by the dynamic component (or predicted precipitation component based on the statistical learning approach).
