# Supplementary material for: Uncertain dynamic response of mid-latitude winter precipitation
Source: Nature. 2026 Apr 29;653(8113):110–6. doi: 10.1038/s41586-026-10474-y (PMC13675132; doi:10.1038/s41586-026-10474-y)
Supplement: Supplementary file 2 [file 41586_2026_10474_MOESM2_ESM.pdf]

Peer Review File

Uncertain dynamic response of mid-latitude winter precipitation

Corresponding Author: Dr Lei Gu

Any redactions in this file are there to maintain patient confidentiality, the confidentiality of unpublished data, or to remove third-party material.

This file contains all reviewer reports in order by version, followed by all author rebuttals in order by version.

Attachments originally included by the reviewers as part of their assessment can be found at the end of this file.

Version 1:

Reviewer comments:

Referee #1

(Remarks to the Author)
Review comments on “Strong dynamic response of mid-latitude winter precipitation to human emissions” by Gu, Schumacher, Sippel, Fischer et al.

This is an interesting study that focuses on the detection of anthropogenic forcing to mid-latitude winter precipitation. While previous studies show that unforced internal variability dominates the observed trends of winter precipitation in the northern hemisphere, the authors have shown strong evidence that the human emissions have driven the mid-latitude winter precipitation change through the dynamic response. To demonstrate this, the authors have developed a novel framework that combines statistical learning across multiple reanalysis datasets and climate model simulations with fully coupled nudged circulation CESM2 climate model experiments. The authors have disentangled the anthropogenic forced thermodynamic and dynamic components from unforced internal variability in winter precipitation trends (1950–2022) across mid- to high-latitude regions. They found that while models capture the thermodynamic component, they underestimate the amplitude of the forced dynamic contribution. The authors further extended their analysis to climate projection and found that the circulation responses will intensify, bringing model projected dynamic trends closer to historical reanalysis, particularly in regions where large-scale circulation changes strongly influence precipitation trends such as the Mediterranean. The design of fully coupled nudged circulation CESM2 model experiments is impressive and helpful to the disentangling of anthropogenic forced thermodynamic and dynamic components. The major findings are novel and convincing. The manuscript was technically well written and easy to follow in logic. I recommend accepting it for publication after some technical revisions listed below.

Major comments
1. Definitions of dynamic and thermodynamic terms: The authors have disentangled the dynamic and thermodynamic drivers of precipitation trends using both statistical learning models and nudged CESM2 experiments. They refer to the total precipitation trend (before decomposition) as ‘full precipitation trend’ and the complete circulation-driven trend as the ‘full dynamic’ component. The residual trend, calculated as the original precipitation trend minus the circulation-induced component, is attributed to thermodynamic contributions. In the simulations, the authors estimate the thermodynamic precipitation trends by subtracting the unforced nudged simulations from the externally forced ones. They further subtract this forced thermodynamic component from the 100-member CESM2 ensemble and finally get the full dynamic precipitation components. We all know that changes in precipitation can be caused by either changes in atmospheric moisture (i.e., thermodynamics), or changes in atmospheric circulation (i.e., dynamics), or both (i.e., nonlinear effects). The contributions of these three terms can be directly diagnosed using moisture budget. If the authors directly compare the thermodynamic/dynamic terms derived from the moisture budget, would the conclusions be the same/similar? Otherwise, the similarity and difference of the definitions employed here to traditional moisture budget-based definitions need to be clarified.
2. The study highlights the strong dynamic response of precipitation to anthropogenic forcing, a quantitative estimation of percentage contribution to total precipitation helps to demonstrate the key finding. The “9.7%” contribution listed in the abstract may be misleading. In addition, the authors have done CESM2 nudging experiments in two ways (L74-77), one was

nudged toward standard historical simulation, another was nudged toward ERA5 reanalysis. I once guessed that the latter should be better than the former in reproducing the observed dynamic precipitation trends, since this procedure is like dynamical downscaling of regional climate models driven by reanalysis. Based on your discussion presented in L238-239, the ERA5 nudged simulation still failed in reproducing the observed dynamic precipitation trends. A deeper discussion on the weakness is appreciated in the revision.

Specific comments:
1. L24: "This component accounts for only 9.7% of the full dynamic trend inferred from reanalysis in circulation-dominated regions." Do you mean the model result is only 9.7% of the reanalysis? Otherwise, it would be difficult to regard a "9.5%" contribution as "strong dynamic response".
3. L25-28: "Under rising anthropogenic emissions, circulation responses are projected to intensify, bringing simulated dynamic trends closer to historical reanalysis, particularly in regions where large-scale circulation changes strongly influence precipitation trends such as the Mediterranean.". What does the statement of "bringing simulated dynamic trends closer to historical reanalysis" mean for future projection?
4. L61: "such discrepancies could stem from misrepresentation of regional responses to anthropogenic emissions", it would be better to clearly describe these misrepresented regional processes.
5. L62: replace "variability-driven mismatches" with "internal variability-driven mismatches", since anthropogenic forcing can also affect the variability.
6. L75: "second, circulations from both pre-industrial and historical–SSP simulations are nudged toward ERA5 reanalysis". &L418-419: "As in experiment 1, the difference between forced and preindustrial simulations is interpreted as the anthropogenic forced thermodynamic component". Based on the experimental design, the only difference between the pre-industrial and historical–SSP simulations here are the specified SST and GHGs forcing? Is this understanding correct?
7. L84-85: The patterns of precipitation increase in ENA and NEU closely resemble the day-by-day variability change of precipitation reported by Zhang et al. (2024 Science). Does this mean these regions are climate sensitive?
8. L90: "winter precipitation in these northern mid-latitudes is dominated by large-scale processes (i.e., cyclones)44,45", the references of 44-45 do not support the statement, please check it.
9. L90-93 & L99-103: You may move these technical model evaluations to the supplementary file.
10. L110-111: "This raises questions on how to understand the significant precipitation trends in observations, and whether simulated winter precipitation responses to anthropogenic emissions reflect 'true signals' in the real world?" , then what is your answer?
11. L153, supplementary Fig. s6a,c: are the patterns similar to the traditional thermodynamic/dynamic terms derived from the moisture budget?
12. L164-166: "thermodynamic trends derived from climate models closely match those from reanalysis (Fig.2), indicating that climate models reliably capture the thermodynamic component of the observed changes in precipitation". It would be better to provide quantitative comparisons of percentage contributions of thermodynamic and dynamic components to total precipitation.
13. L194-196: "However, the 'most likely simulation' (Methods) fails to capture this shift in atmospheric dynamics over recent decades (Supplementary Fig. S7d-g), highlighting the inability of climate models to reproduce observed dynamic precipitation trends in Europe". Since the "most likely simulation" is defined based on the high correlation between the dynamic (or thermodynamic) precipitation components from models and the dynamic component derived from ERA5 reanalysis, does this statement mean that these two model realizations reproduce the precipitation component reasonably with incorrect circulation background?
14. L210-211: again, a quantitative comparison is appreciated.
15. L238-239: even the ERA5 nudged simulation still failed in reproducing the observed dynamic precipitation trends, any explanation for this? I once hope that the ERA5 nudged simulation should be better than the freely coupled historical–SSP simulations in this regards.
16. L259: After " In reanalysis, dynamic trends are strongly negative across most grid cells", add "in SEU".
17. Figure 4: no discussion was given for Fig.4g,h,i in the text. In addition, the curves shown on Fig.4 are not clear.
18. L290: "our nudged climate model experiments offer a robust framework for decomposing winter precipitation trends into dynamic and thermodynamic components". Again, it would be better to clarify the differences to traditional dynamic and thermodynamic definitions.
19. L331: "The projected decline in dynamic precipitation trends closely resembles the current ERA5 pattern, lending further support to our interpretation". I cannot understand why the resemblance of future projection to the current observation supports your interpretation here.

(Remarks on code availability)

Referee #2

(Remarks to the Author)
This manuscript compares winter precipitation trends from 1950-2022 in the Northern Hemisphere extratropics across observations, reanalysis, and earth system models. They focus on three regions where the trend is robustly significant: Eastern North America (getting wetter), Northern Europe (getting wetter), and Southern Europe (getting drier). On average, models predict similar patterns in the trend across these three regions as obs/reanalysis but underestimate the magnitude. To understand the source of this discrepancy the authors decompose the trend into combinations of forced and unforced and dynamic and thermodynamic components of the full precipitation trend.

The methods used in the decomposition are derived from past work (e.g., Sippel et al. 2019 for the statistical method used across all datasets including obs/reanalysis and Wehrli et al. 2018 for nudging CESM2 horizontal winds to various target states while keeping the forcing and land use fixed at piControl). The idea of using a statistical method like regularized linear regression across observations and models and validating it using a large ensemble is not entirely new (e.g., ForceSMIP) but has not yet been published for the precipitation trends in focus here. Thus to my knowledge the analysis provided here is novel. The findings are important (and some surprising, such as the significant spatial heterogeneity of the thermodynamic component) and seems worthy of publishing in Nature since it follows up on work that was recently published in the same journal (Vicente-Serrano et al. 2025).

However, there is some overlap in the analysis of the Southern Europe region in this study and recently published work (Doane-Solomon et al. 2025). Interestingly, the conclusions drawn are not the same. In this manuscript the dynamic component in the Mediterranean region is poorly represented by models while the residual (thermodynamic) component is better represented at least with ERA5. In Doane-Solomon et al. 2025 the dynamic component is well represented by models but not the residual. This may be due to differences in the analysis time period and/or differences in the way dynamical adjustment is made. I encourage the authors to investigate this further.

In addition to the omission of relevant literature, there are several other concerns that I would like to see the authors address if this manuscript were to be published. I detail them below. Overall, I can see this manuscript being fit for publication if these concerns are addressed.

Osamu Miyawaki
Dec 3, 2025

- The manuscript focuses on 1950-2022 when Vicente-Serrano et al. (2025) show Mediterranean precipitation trends are sensitive to the considered time period. Doane-Solomon (2025) confirm this, showing trends from 1979-2022 are not statistically significant over the Mediterranean. How sensitive are the conclusions to the time period chosen? Can the authors better motivate why 1950-2022 was chosen?
- There are several statements in the manuscript that aren't sufficiently supported by the presented data. Line 164 states "thermodynamic trends derived from climate models closely match those from reanalysis"but the thermodynamic component of the precipitation response over the Mediterranean using the CRU-NCEP dataset is outside of the climate model distribution. Sensitivity analysis with other reanalysis datasets such as MERRA2 and JRA3Q would be valuable to better quantify the obs/reanalysis spread. Line 194-195 states "the most likely simulation fails to capture this shift in atmospheric dynamics over recent decades" but Supplementary Fig. S7d-g largely show similar large-scale patterns of SLP and u500 as in ERA5. The difference between reanalysis and models seem more subtle than stated in the manuscript, i.e. the issues involve small differences in the magnitude of the trends (CESM2 72 underestimates the magnitude of trends over the North Atlantic) or the exact position of the dipole (e.g. FGOALS dipole is too ear to the east). Condensing this as "fails to capture this shift" seems misleading.
- The assumptions involved in the decomposition (line 403-406) limit the interpretability of the dynamic/thermodynamic decomposition. Comparing the decomposed terms with the maps of SLP, u500, huss, and ET is a useful start to validating the decomposition but the presented analysis is largely qualitative and hand-wavy (e.g. line 199-200). Adding to this concern is that some datasets like CRU show significant anti-correlation between the dynamic and thermodynamic components (Supplementary Figure 6). Unless there is a physical explanation for this anti-correlation I suspect higher-order terms (interaction between dynamics and thermodynamics) may be important. Thus assuming the residual as a purely "thermodynamic" contribution is misleading, especially so to a broad audience such as Nature's (e.g., see the Discussion section in Doane-Solomon et al. 2025 for a more cautious perspective on this).
- It would be nice to see how the methods presented here compare to even simpler methods like in Emori and Brown (2005). In that framework they find the covariance between dynamic and thermodynamic contributions are weak. If the dynamic and thermodynamic components as derived using Emori and Brown (2005) are similar to the methods used here, it may help alleviate my concerns about interpretability above.
- It is not clear to me why a single-member multi-model CMIP6 ensemble is included here. This is not sufficiently motivated in the manuscript. A seemingly more relevant ensemble is the subset of models that run single-model initial-condition large ensembles (SMILE). This work looks at one such dataset (CESM2-LE) but there are several others in the CMIP6 family forced with the SSP370 scenario (e.g., see Maher et al. 2023). Doane-Solomon et al. (2025) consider multiple SMILE ensembles and show the forced component of the precipitation response in the Mediterranean differ in sign across SMILEs. This is a more insightful quantification of model uncertainty of the forced response than can be obtained from a multi-model ensemble of the r1i1p1f1 run.
- The manuscript misses some key references/comparisons to relevant work, e.g. Seager et al. (2024), Doane-Solomon et al. (2025).

Emori, S., & Brown, S. J. (2005). Dynamic and thermodynamic changes in mean and extreme precipitation under changed climate. Geophysical Research Letters, 32(17), 529.

Wehrli, K., Guillod, B. P., Hauser, M., Leclair, M., & Seneviratne, S. I. (2018). Assessing the Dynamic Versus Thermodynamic Origin of Climate Model Biases. Geophysical Research Letters, 45(16), 8471-8479.

Maher, N., Wills, R. C. J., DiNezio, P., Klavans, J., Milinski, S., Sanchez, S. C. et al. (2023). The future of the El Niño–Southern Oscillation: using large ensembles to illuminate time-varying responses and inter-model differences. Earth System Dynamics, 14(2), 413-431.

Seager, R., Wu, Y., Cherchi, A., Simpson, I. R., Osborn, T. J., Kushnir, Y. et al. (2024). Recent and near-term future changes in impacts-relevant seasonal hydroclimate in the world's Mediterranean climate regions. International Journal of Climatology, 44(11), 3792-3820.

Doane-Solomon, R., Woollings, T., & Simpson, I. R. (2025). Dynamic Contributions to Recent Observed Wintertime Precipitation Trends in Mediterranean-Type Climate Regions. Geophysical Research Letters, 52(12).

(Remarks on code availability)
I did not review the code because I am not familiar with R.

Referee #3

(Remarks to the Author)
Previous studies have shown that climate models tend to underestimate North Atlantic atmospheric circulation trends, including associated precipitation trends in North America and Europe, with observed trends being at the edge of the model spread. This mismatch is due to either 1) the models underestimating the forced trend or 2) the models underestimating internal variability, or 3) an unusually large realization of internal variability in the observed record. This paper investigates this issue in more detail by using two novel methods to distinguish between thermodynamic and dynamic parts of observed circulation and precipitation trends. The two methods, one based on nudged climate model simulations and the second on an advanced linear regression model, are novel, and provide some interesting results. However, the fundamental flaw of the paper is that the title and abstract grossly overstate the conclusions presented in the main paper. This is further explained in my main comment.

Main comment:

The title of the paper claims that 'human emissions' have led to strong circulation changes that have led to strong midlatitude winter precipitation trends. In addition, the abstract claims that models 'underestimate the amplitude of the forced dynamic contribution'. However, such strong statements are not supported by the main body of the paper. These statements are based on the finding that the forced dynamic part of the precipitation trends is smaller than the observed full dynamic trend, which is the result of anthropogenic forcing plus internal variability (e.g. line 227-235). The mismatch between the observed full dynamic trend and modelled forced dynamic trend could be due to either 1) the forced trends being underestimated by the models (as the authors claim in the abstract) or 2) large internal variability in the observations. The authors have not shown any evidence that can distinguish between 1) and 2).

For the authors to show that the forced trends are in reality 'strong', and that models underestimate this response, the authors would have to quantify the observed forced dynamic trend. The fundamental problem, however, is that the forced response in observations cannot be determined because it represents a single realization. The observed trends are the result of the forced response plus internal variability, and the study is not able to quantify the relative magnitude of the two components, as also stated in l. 274-275 ('Because ERA5 reanalysis reflects a single realization, ideal separation into internal, forced dynamic and thermodynamic components is not possible'). In short, I did not find any evidence to support the notion that the observed trends are mainly the result of human emissions, as the trends could also have been caused by internal variability.

Compared to the claims made in the title and abstract, the authors are more cautious and balanced in the main paper stating that '.., climate models likely underestimate the circulation response to human forcings' (l. 237) and 'climate models likely underestimate the magnitude of the externally forced circulation changes in this region.' (l. 263) and 'This suggests that current climate models may underestimate forced dynamic contributions…' (l. 307). But this caution is not reflected in the abstract nor title of the paper, which I therefore find very misleading.

Other comments:
1) In some instances, it hard to follow the reasoning of the authors due to (to me) non-intuitive notations. For example, I don't understand why the pre-industrial simulations with nudged circulation from the historical/ssp370 simulations are denoted by 'Nudged-Ex3'.
2) l. 164-165: This statement does not seem to be consistent with Figure 2b, with the CRU-NCEP derived thermodynamic trend being outside the model spread.
3) L. 185-186: 'the most likely simulation'. Not clear what is meant with that. After some digging I see that a description is given in the methods section, but that description is unclear as well. Is it the ensemble member with the highest spatial or temporal correlation? Why is that selected, and how should it be interpreted?
4) L. 217: Not clear to me what is exactly meant with 'the multi-model mean signal'
5) L. 225: 'These result imply that thermodynamic regulation of precipitation is largely driven by anthropogenic forcing'. This is too strong a statement. I would change this to something like 'these results suggest that in the observed record, changes in thermodynamic regulators of precipitation are mainly driven by anthopogenic forcing, with internal variability playing a secondary role'
6) L. 251: 'access' 'assess'?

(Remarks on code availability)

Version 2:

Reviewer comments:

Referee #1

(Remarks to the Author)
This is the revised version of the manuscript that I have previously reviewed. I would like to thank the authors for their efforts in revising the manuscript and for their point-by-point responses to my comments. The authors have performed a significant amount of additional work, especially for the comparison of thermodynamic and dynamic components of precipitation derived from traditional moisture budget-based estimation and their definition. My concerns regarding the technical details for model experiments design are also clarified. The revisions have addressed nearly all my initial concerns. I recommend accepting it for publication in Nature after some further minor and technical revisions.

Specific comments:
1) In response to my major comment#2, the authors explained that "the Forced DY (Nudged-ERA5) is less reliable: it is derived by subtracting forced TD (Nudged-ERA5) from CESM2-LE ensemble mean, but the ERA5-nudging uses a prescribed ocean while CESM2-LE is fully interactive. This might explain why the improved constraint from ERA5 winds does not translate into a cleaner forced DY estimate". These distinctions are discussed in the revised "Attribution to external forcing" section as "Differences between the two configurations partly reflect contrasting ocean boundary conditions: the free-running setup uses a fully coupled ocean, whereas the ERA5-nudged configuration prescribes oceans." The weakness of experimental design can be acknowledged in the "Constrained climate model with ERA5 winds (experiment 2)" part of Method section.
2) Fig. 1. Observational and simulated trends in winter precipitation 1950–2022. Please show the results of Fig.1e-g derived from the CMIP6 MME and CESM2-LE MME as supplementary figure. This helps the readers to quantitatively understand the weaknesses of climate models.
3) L139-148："In parallel, we run nudged CESM2 experiments that take dynamic conditions from free running historical and future (SSP3.7-0) simulations with anthropogenic greenhouse gas and aerosol emissions as well as land use changes, and prescribe these dynamic conditions to control simulations without external forcing (Nudged-F). To assess the influence of observed circulation patterns, we additionally run CESM2 nudged experiments using horizontal winds from ERA5 (Nudged-E). Subtracting the unforced nudged simulations from the externally forced ones yields idealized thermodynamic precipitation trends (Fig. 2,TD(Nudged)). Further subtracting this forced thermodynamic component from the 100-member CESM2 ensemble produces full dynamic precipitation components (Fig. 2, DY(Nudged)) that are independent of the statistical learning approach". It is a bit complex for the readers to understand this statement, especially for those who are not expertized in climate modeling. To help the readers easily understand the modeling strategy, you may add one Table in the Method section and thus clarify how the thermodynamic and dynamic components are estimated/derived.
4) L202-204: "Accordingly, the simulation that best reproduces dynamic precipitation trends does not correspond to the highest correlations in either SLP or U500. This indicates limitations of climate models to reproduce observed dynamic precipitation trends in Europe." One interesting question here is: where do the best-matching dynamic precipitation trends come from? Any explanation?
5) L457-459: "Specifically, this experiment is conducted in an atmosphere-only configuration (AMIP style) with prescribed ocean boundary conditions." AMIP type simulation means that the AGCM was driven by historical SST with year by year change. I understand that your sea surface temperature is derived from the preindustrial run and thus does not have observed year by year variability. Is this understanding correct?
6) L463-465: "We find that precipitation trends from the CESM2 forced simulation closely match those from ERA5, confirming the robustness of externally applied forcing in climate models (Supplementary Fig. 13)." In climate modelling community, we generally used the term "forced simulation" to describe the coupled model historical simulations driven by anthropogenic and natural external forcing factors. To avoid misunderstanding, I would suggest you replace "the CESM2 forced simulation" with "the nudged CESM2 AGCM simulation".

(Remarks on code availability)

Referee #2

(Remarks to the Author)
The authors have made substantial revisions in response to the other reviewers' and my comments. The revised title and abstract are more balanced and accurately reflect the study's findings. The addition of JRA-3Q data, moisture budget diagnostics, and omega500 binning following Emori and Brown (2005) address my previous concerns about the interpretability and robustness of the dynamic/thermodynamic decomposition. I have one minor comment:

Line 91-92: Climate sensitivity refers to the temperature response to a doubling of CO_2. Increased regional precipitation variability does not directly imply elevated climate sensitivity in the standard definition of climate sensitivity. Consider removing the reference to climate sensitivity.

(Remarks on code availability)

Referee #3

(Remarks to the Author)
The authors have toned down the overly strong statements in the original submission, addressing my main concerns. I don't have any further comments, except that the following sentence should be improved: (line 169-170): 'Overall, our results suggest that despite such biases may affect climatology, thermodynamic precipitation trends are captured by climate models with high confidence', as it does not read well.

(Remarks on code availability)

Open Access This Peer Review File is licensed under a Creative Commons Attribution 4.0 International License, which permits use, sharing, adaptation, distribution and reproduction in any medium or format, as long as you give appropriate credit to the original author(s) and the source, provide a link to the Creative Commons license, and indicate if changes were made.
In cases where reviewers are anonymous, credit should be given to 'Anonymous Referee' and the source.
The images or other third party material in this Peer Review File are included in the article's Creative Commons license, unless indicated otherwise in a credit line to the material. If material is not included in the article's Creative Commons license and your intended use is not permitted by statutory regulation or exceeds the permitted use, you will need to obtain permission directly from the copyright holder.
To view a copy of this license, visit https://creativecommons.org/licenses/by/4.0/

Reply to Reviewers' comments

Legend
Reviewers' comments
Authors' responses
Direct quotes from the revised manuscript

Reviewer #1 Evaluations:
This is an interesting study that focuses on the detection of anthropogenic forcing to mid-latitude winter precipitation. While previous studies show that unforced internal variability dominates the observed trends of winter precipitation in the northern hemisphere, the authors have shown strong evidence that the human emissions have driven the mid-latitude winter precipitation change through the dynamic response. To demonstrate this, the authors have developed a novel framework that combines statistical learning across multiple reanalysis datasets and climate model simulations with fully coupled nudged circulation CESM2 climate model experiments. The authors have disentangled the anthropogenic forced thermodynamic and dynamic components from unforced internal variability in winter precipitation trends (1950–2022) across mid- to high-latitude regions. They found that while models capture the thermodynamic component, they underestimate the amplitude of the forced dynamic contribution. The authors further extended their analysis to climate projection and found that the circulation responses will intensify, bringing model projected dynamic trends closer to historical reanalysis, particularly in regions where large-scale circulation changes strongly influence precipitation trends such as the Mediterranean. The design of fully coupled nudged circulation CESM2 model experiments is impressive and helpful to the disentangling of anthropogenic forced thermodynamic and dynamic components. The major findings are novel and convincing. The manuscript was technically well written and easy to follow in logic. I recommend accepting it for publication after some technical revisions listed below.

Reply: We thank the reviewer for the supportive and insightful feedback. In the revised manuscript, we have clarified the definition of dynamic versus thermodynamic contributions. Additionally, we have included another reanalysis dataset – JRA3Q, which is available from 1950 – together with ERA5 to perform moisture budget diagnostics and a simple empirical method (based on daily 500 hPa vertical velocity) to systematically decompose precipitation trends into different components. These additional analyses further support our main conclusions. We hope you find the revised version suitable for publication.

Major comments

1. Definitions of dynamic and thermodynamic terms: The authors have

disentangled the dynamic and thermodynamic drivers of precipitation trends using both statistical learning models and nudged CESM2 experiments. They refer to the total precipitation trend (before decomposition) as 'full precipitation trend' and the complete circulation-driven trend as the 'full dynamic' component. The residual trend, calculated as the original precipitation trend minus the circulation-induced component, is attributed to thermodynamic contributions. In the simulations, the authors estimate the thermodynamic precipitation trends by subtracting the unforced nudged simulations from the externally forced ones. They further subtract this forced thermodynamic component from the 100-member CESM2 ensemble and finally get the full dynamic precipitation components. We all know that changes in precipitation can be caused by either changes in atmospheric moisture (i.e., thermodynamics), or changes in atmospheric circulation (i.e., dynamics), or both (i.e., nonlinear effects). The contributions of these three terms can be directly diagnosed using moisture budget. If the authors directly compare the thermodynamic/dynamic terms derived from the moisture budget, would the conclusions be the same/similar? Otherwise, the similarity and difference of the definitions employed here to traditional moisture budget-based definitions need to be clarified.

Reply: We appreciate your consolidated comments to strengthen our analysis. Following your suggestions, we have employed a new reanalysis dataset (JRA-3Q, 45 pressure levels), together with ERA5 (37 pressure levels), to perform the proposed moisture budget analysis. Specifically, the moisture budget framework decomposes changes in precipitation and evapotranspiration into contributions from horizontal (u, v) and vertical (ω) moisture advection (Chou and Lan, 2012; Jiang et al., 2023). Moreover, we have applied another simple method which takes daily mean 500hPa vertical velocity as a proxy of the strength of dynamic disturbance (suggested by **Reviewer 2**, Emori and Brown, 2005) to decompose precipitation trends into dynamic, thermodynamic, and nonlinear contributions. We have revised the ***Outlook*** (Second paragraph) section as follows:

In this study, thermodynamic influence is defined broadly to encompass all non-circulation factors, such as changes in lapse rate, land–sea thermal contrast, evapotranspiration, humidity, transient eddies, and nonlinear effects. This definition is reflected in the thermodynamic contributions identified by the moisture budget analysis (Chou and Lan, 2012; Jiang et al., 2023). This moisture budget method typically restricts thermodynamic effects to temperature- and humidity-related changes in vertical and horizontal moisture advection, thus yielding different thermodynamic partitioning relative to our statistical learning approach (Supplementary Figs. 16–17). By contrast, dynamic contributions to total precipitation trends are generally consistent across the two methods, supporting the robustness of the decomposition. To provide an additional comparison, we also apply an empirical binning approach using 500hPa

vertical velocity as the proxy for the strength of dynamic disturbance (Emori and Brown, 2005). Although the magnitude of the dynamic contribution obtained from this method is systematically smaller, the sign is consistent with results from the statistical learning and moisture budget. This further confirms that the circulation-wetting in NEU/ENA and circulation-drying in SEU is not an artifact.

Chou, C., & Lan, C. W. (2012). Changes in the annual range of precipitation under global warming. Journal of Climate, 25(1), 222-235.

Jiang, J., Zhou, T., Qian, Y., et al. (2023). Precipitation regime changes in High Mountain Asia driven by cleaner air. Nature, 623(7987), 544-549.

Emori, S., & Brown, S. J. (2005). Dynamic and thermodynamic changes in mean and extreme precipitation under changed climate. Geophysical Research Letters, 32(17).

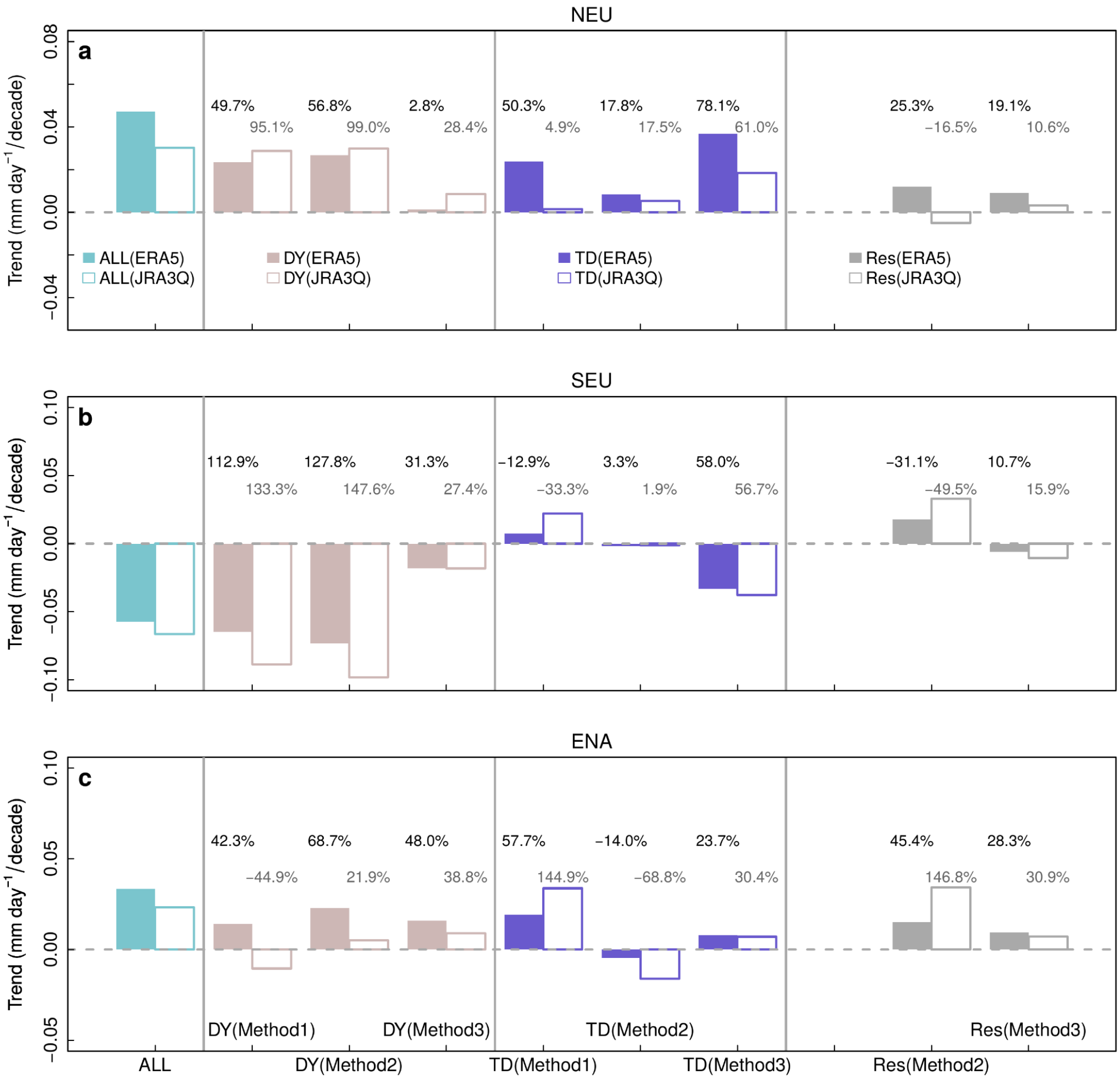

Supplementary Figure 16. Dynamic and thermodynamic contribution to winter precipitation trends. a, Winter precipitation trends over the northern Europe (NEU) during 1950–2022 (ALL) decomposed into dynamic (DY), thermodynamic (TD), and residual (Res) components using ERA5 and JRA-3Q reanalysis datasets. Method 1 represents the elastic-net model; Method 2 represents the moisture budget analysis; Method 3 represents results using daily mean 500 hPa vertical velocity as the proxy of dynamic disturbance. **b**, **c**, The same as (**a**), but for total and decomposed trends over southern Europe (SEU) and over eastern North America (ENA). The black (grey) numbers

represent contributions from ERA5 (JRA-3Q) dataset.

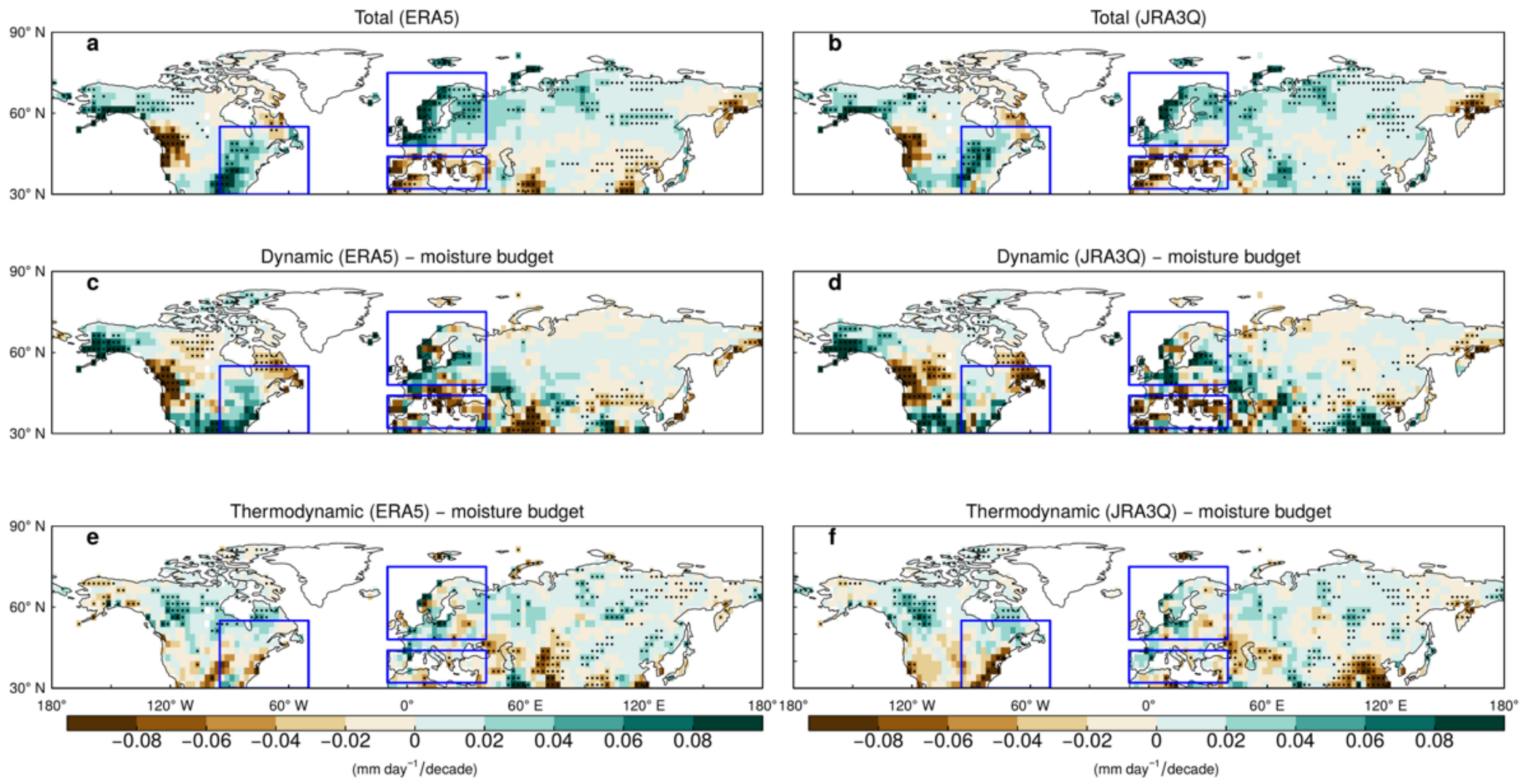

Supplementary Figure 17. Dynamic and thermodynamic winter precipitation trends based on moisture budget diagnostics. a,**c**,**e**, The total (**a**), dynamic (**c**), and thermodynamic (**e**) winter precipitation trends based on moisture budget diagnostics from the ERA5 reanalysis dataset. Note that the dynamic and thermodynamic trends include respective contributions from both horizontal and vertical moisture advection. **b**,**d**,**f**, The same as **a**,**c**,**e**, but from the JRA-3Q reanalysis dataset. The dot stippling represents significant trends at the 0.05 level (p-value < 0.5).

2. The study highlights the strong dynamic response of precipitation to anthropogenic forcing, a quantitative estimation of percentage contribution to total precipitation helps to demonstrate the key finding. The "9.7%" contribution listed in the abstract may be misleading. In addition, the authors have done CESM2 nudging experiments in two ways (L74-77), one was nudged toward standard historical simulation, another was nudged toward ERA5 reanalysis. I once guessed that the latter should be better than the former in reproducing the observed dynamic precipitation trends, since this procedure is like dynamical downscaling of regional climate models driven by reanalysis. Based on your discussion presented in L238-239, the ERA5 nudged simulation still failed in reproducing the observed dynamic precipitation trends. A deeper discussion on the weakness is appreciated in the revision.

Reply: We apologize for our previous unclear statements. We have revised the **Abstract** to better convey our key finding, please refer to the following revision.

The reviewer's expectation is, in principle, correct: nudging to ERA5 winds should better constrain the simulation towards observed circulation pattern. This expectation, however, holds more cleanly for total precipitation and the thermodynamic component than for the dynamic component. The ERA5-nudged simulation serves as a useful overall check: total precipitation trends in the forced AMIP run closely match ERA5 reanalysis (Supplementary Fig. 13), providing confidence that CESM2 responds plausibly to observed circulation. Specifically, forced TD (Nudged-ERA5) is internally

consistent: both the forced and preindustrial AMIP-style runs use prescribed oceans, so their difference robustly isolates the forced thermodynamic response. Forced DY (Nudged-ERA5) is less reliable: it is derived by subtracting forced TD (Nudged-ERA5) from CESM2-LE ensemble mean, but the ERA5-nudging uses a prescribed ocean while CESM2-LE is fully interactive. This might explain why the improved constraint from ERA5 winds does not translate into a cleaner forced DY estimate. These distinctions and their implications are now discussed in detail in the revised ***Attribution to external forcing*** section as follows:

Abstract: The thermodynamic contribution is broadly reproduced by climate models, whereas the dynamic contribution can exceed model estimates. In circulation-dominated regions such as the Mediterranean, the forced dynamic signal from model simulations explains only 9.7% of the observed dynamic trend, rendering detection challenging. Under continued anthropogenic emissions, projected circulation response intensifies and more closely resembles present-day reanalysis patterns. Although internal variability in observations may contribute to this similarity, the results suggest an uncertain yet potentially emerging role of dynamic response in shaping regional winter precipitation trends.

Attribution to external forcing section: In the nudging experiments (Fig. 3e-h), the thermodynamic component is estimated directly from paired simulations that share identical circulations but differ in anthropogenic forcing. Three such pairs are available in the free-running configuration (Fig. 3e,f), whereas only one pair is available in the ERA5-nudged configuration (Fig. 3g,h). We effectively assume that internal thermodynamic variability is (i) smaller than the forced component and (ii) easily averaged out. The consistency of thermodynamic changes across the three free-running pairs supports this assumption (Supplementary Fig. 12), although it cannot be independently verified for the single ERA5-nudged pair. The forced dynamic contribution is then obtained by subtracting the nudging-based thermodynamic estimate from the CESM2-LE ensemble-mean. Differences between the two configurations partly reflect contrasting ocean boundary conditions: the free-running setup uses a fully coupled ocean, whereas the ERA5-nudged configuration prescribes oceans. Despite these structural differences, both nudging approaches yield results broadly consistent with the statistical learning framework. Across all four methods, the forced thermodynamic contribution is positive over NEU and ENA (Fig. 3b,d,f,h), while the forced dynamic contribution is predominantly negative over SEU (Fig. 3a,c,e,g). Although the magnitude of the forced dynamic signal over SEU remains uncertain, its negative sign is robust across statistical learning approaches (Fig. 3a,c) and physical model experiments (Fig. 3e,g).

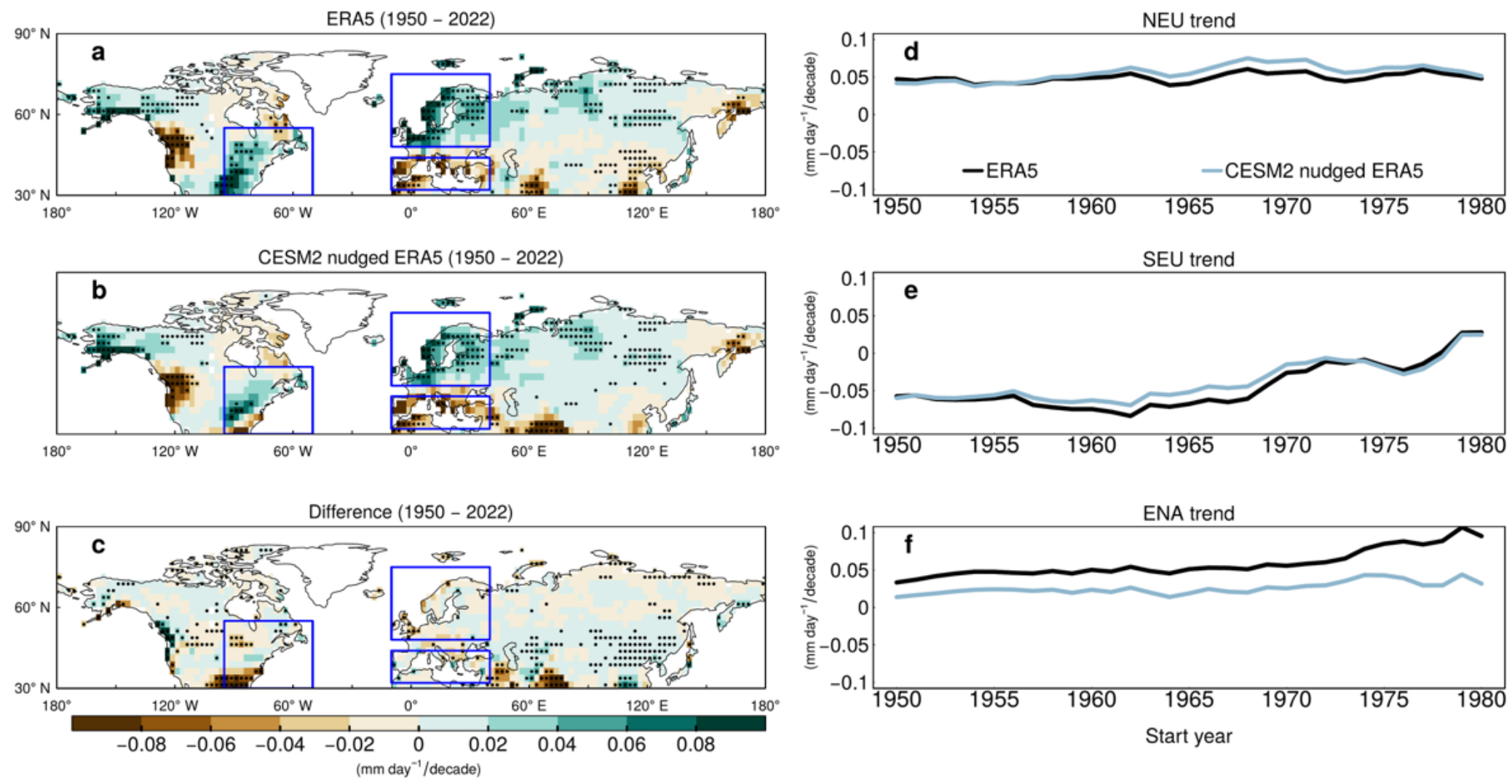

Supplementary Figure 13. Comparison of winter precipitation trends between ERA5 and CESM2 nudged to ERA5 simulation. a, Winter precipitation trends from ERA5 reanalysis. **b,** Winter precipitation trends estimated by the CESM2 forced simulation driven with ERA5 wind background. **c,** The difference of winter precipitation trends between the CESM2 forced simulation and the ERA5 reanalysis. The dot stippling represents significance at the 0.05 level (p-value < 0.05). **d-f**, The magnitude of linear trends from the ERA5 reanalysis and the CESM2 nudged ERA5 simulation ending in 2022 as a function of start year over northern Europe (NEU, **d**), southern Europe (SEU, **e**), and eastern North America (ENA, **f**).

Specific comments:

1. L24: "This component accounts for only 9.7% of the full dynamic trend inferred from reanalysis in circulation-dominated regions." Do you mean the model result is only 9.7% of the reanalysis? Otherwise, it would be difficult to regard a "9.7%" contribution as "strong dynamic response".

Reply: Thank you for pointing this out. Yes, the forced dynamic contribution only accounts for 9.7% of the reanalysis dynamics over SEU, leaving the residual–unforced dynamic contribution–unexpectedly large. Therefore, we argue that climate models may underestimate forced dynamic component in the real world and the 'true' dynamic response may be strong(er). We have revised the ***Abstract*** as follows:

Understanding changes in precipitation is crucial for ecosystems, climate resilience, and human society[1,2,3]. Studies have documented the respective contributions of anthropogenic forcing and internal variability to precipitation trends[4,5,6,7], yet discrepancies persist between observed and simulated patterns. In Northern Hemisphere winter, these mismatches are often attributed to unforced internal variability that dominates observed trends[8]. However, growing evidence also indicates that climate models underestimate the total response of precipitation to human forcings[9,10,11]. Here, we develop a novel framework that combines statistical learning across multiple reanalysis datasets and climate model simulations with fully coupled nudged-circulation CESM2 climate model experiments. We systematically disentangle the anthropogenic forced thermodynamic and dynamic components from internal

variability in winter precipitation trends (1950–2022) across mid-latitude regions. The thermodynamic contribution is broadly reproduced by climate models, whereas the dynamic contribution can exceed model estimates. In circulation-dominated regions such as the Mediterranean, the forced dynamic signal from model simulations explains only 9.7% of the observed dynamic trend, rendering detection challenging. Under continued anthropogenic emissions, projected circulation response intensifies and more closely resembles present-day reanalysis patterns. Although internal variability in observations may contribute to this similarity, the results suggest an uncertain yet potentially emerging role of dynamic response in shaping regional winter precipitation trends.

2. L25-28: "Under rising anthropogenic emissions, circulation responses are projected to intensify, bringing simulated dynamic trends closer to historical reanalysis, particularly in regions where large-scale circulation changes strongly influence precipitation trends such as the Mediterranean.". What does the statement of "bringing simulated dynamic trends closer to historical reanalysis" mean for future projection?.

Reply: Our signal-to-noise ratio analysis indicates that future projections exhibit increasingly robust sea level pressure signals, thereby reducing the discrepancy between reanalysis-based trends and the ensemble mean response. This implies that circulation-related signals emerge earlier in reanalysis than that suggested by climate model simulations. We have rephrased the statement in the revised ***Abstract*** as follows:

In circulation-dominated regions such as the Mediterranean, the forced dynamic signal from model simulations explains only 9.7% of the observed dynamic trend, rendering detection challenging. Under continued anthropogenic emissions, projected circulation response intensifies and more closely resembles present-day reanalysis patterns. Although internal variability in observations may contribute to this similarity, the results suggest an uncertain yet potentially emerging role of dynamic response in shaping regional winter precipitation trends.

3. L61: "such discrepancies could stem from misrepresentation of regional responses to anthropogenic emissions", it would be better to clearly describe these misrepresented regional processes.

Reply: Thanks for this useful comment, we have included the possible misrepresented regional processes in the revised ***Introduction*** section as follows:

Current climate models do not reproduce the response of North Atlantic jet stream to external forcing (Smith et al., 2025; Carruthers et al., 2025). In addition, the biased representation of climate change effects on precipitation-temperature-humidity coupling in climate models also contributes to the mismatch (Lenderink et al., 2025).

Carruthers, James G., Hayley J. Fowler, Daniel Bannister, and Selma B. Guerreiro. "Dynamical adjustment reveals spatial patterns of wetting and drying in European winter precipitation." Environmental Research Letters 20, no. 11 (2025): 114085.
Lenderink, Geert, Nikolina Ban, Erwan Brisson, Ségolène Berthou, Virginia Edith Cortés-Hernández, Elizabeth Kendon, Hayley Fowler, and Hylke de Vries. "Are dependencies of extreme rainfall on humidity more reliable in convection-permitting climate models?." Hydrology and Earth System Sciences Discussions 2024 (2024): 1-31.
Smith, D. M., N. J. Dunstone, R. Eade, S. C. Hardiman, L. Hermanson, A. A. Scaife, and M. Seabrook. "Mitigation needed to avoid unprecedented multi-decadal North Atlantic Oscillation magnitude." Nature Climate Change (2025): 1-8.

4. L62: replace “variability-driven mismatches” with “internal variability-driven mismatches”, since anthropogenic forcing can also affect the variability.

Reply: Thanks very much and done.

5. L75: “second, circulations from both pre-industrial and historical–SSP simulations are nudged toward ERA5 reanalysis”. &L418-419: “As in experiment 1, the difference between forced and preindustrial simulations is interpreted as the anthropogenic forced thermodynamic component”. Based on the experimental design, the only difference between the pre-industrial and historical–SSP simulations here are the specified SST and GHGs forcing? Is this understanding correct?

Reply: Yes, the only difference between the pre-industrial and historical-SSP simulations are GHG forcing, land-use and land-cover distributions, and the **prescribed** sea surface temperature (SST) and sea ice concentration (SIC). Notably, this experiment is conducted in an AMIP-style configuration, whereas the free-running nudging experiments are performed in a fully coupled framework. We have expanded the details in the revised ***Method*** as follows:

Specifically, this experiment is conducted in an atmosphere-only configuration (AMIP style) with prescribed ocean boundary conditions. The sea surface temperature for the preindustrial simulation is estimated by removing the forced component from the HadISST using a low-frequency filtering method[65]. The relationship between sea surface temperature and sea ice is derived from the HadISST dataset, and the preindustrial sea ice concentration is subsequently generated based on the reconstructed pre-industrial sea surface temperature.

6. L84-85: The patterns of precipitation increase in ENA and NEU closely resemble the day-by-day variability change of precipitation reported by Zhang et al. (2024 Science). Does this mean these regions are climate sensitive?

Reply: Agree. We have briefly included this in the revised ***Recent precipitation trends in boreal winter*** section as follows:

ENA and NEU exhibit significant increases in precipitation (0.03 and 0.05 mm day^{-1}/decade) while SEU shows a significantly negative trend of –0.06 mm day^{-1}/decade, despite some grid-scale discrepancies among datasets (Supplementary 2). The upward trends in ENA and NEU are accompanied by increasing precipitation variability (Zhang et al., 2024), suggesting elevated climate sensitivity and an intensified hydrological cycle in these regions.

Zhang, W., Zhou, T. & Wu, P. Anthropogenic amplification of precipitation variability over the past century. *Science* 385, 427–432 (2024).

7. L90: "winter precipitation in these northern mid-latitudes is dominated by large-scale processes (i.e., cyclones)44,45", the references of 44-45 do not support the statement, please check it.

Reply: We have replaced the references as follows:

Winter precipitation in these northern mid-latitudes is dominated by large-scale processes (Ummenhofer et al., 2017).

Ummenhofer, C. C., Seo, H., Kwon, Y. O., Parfitt, R., Brands, S., & Joyce, T. M. (2017). Emerging European winter precipitation pattern linked to atmospheric circulation changes over the North Atlantic region in recent decades. Geophysical Research Letters, 44(16), 8557-8566.

8. L90-93 & L99-103: You may move these technical model evaluations to the supplementary file.

Reply: We have moved these model evaluations to the supplementary file.

9. L110-111: "This raises questions on how to understand the significant precipitation trends in observations, and whether simulated winter precipitation responses to anthropogenic emissions reflect 'true signals' in the real world?", then what is your answer?

Reply: We have briefly discussed this point in the revised ***Outlook*** (Second paragraph) section as follows:

Observed significant trends reflect the combined effects of internal variability, thermodynamic and dynamic precipitation response to external forcing. Simulated winter precipitation response to anthropogenic emissions do not always capture the full

magnitude of signals observed in the real world, though internal variability may account for much of this discrepancy.

10. L153, supplementary Fig. s6a,c: are the patterns similar to the traditional thermodynamic/dynamic terms derived from the moisture budget?

Reply: Please refer to the response to **Major comment 1** for a thorough comparison and discussion.

11. L164-166: "thermodynamic trends derived from climate models closely match those from reanalysis (Fig.2), indicating that climate models reliably capture the thermodynamic component of the observed changes in precipitation". It would be better to provide quantitative comparisons of percentage contributions of thermodynamic and dynamic components to total precipitation.

Reply: Thanks for this insightful suggestion! To assess whether the decomposed dynamic and thermodynamic precipitation trends encompass observation-based trends, we have revised Fig. 2 to explicitly label the range of simulated percentage contributions alongside the decomposed contributions from reanalysis (using ERA5 reanalysis as the benchmark).

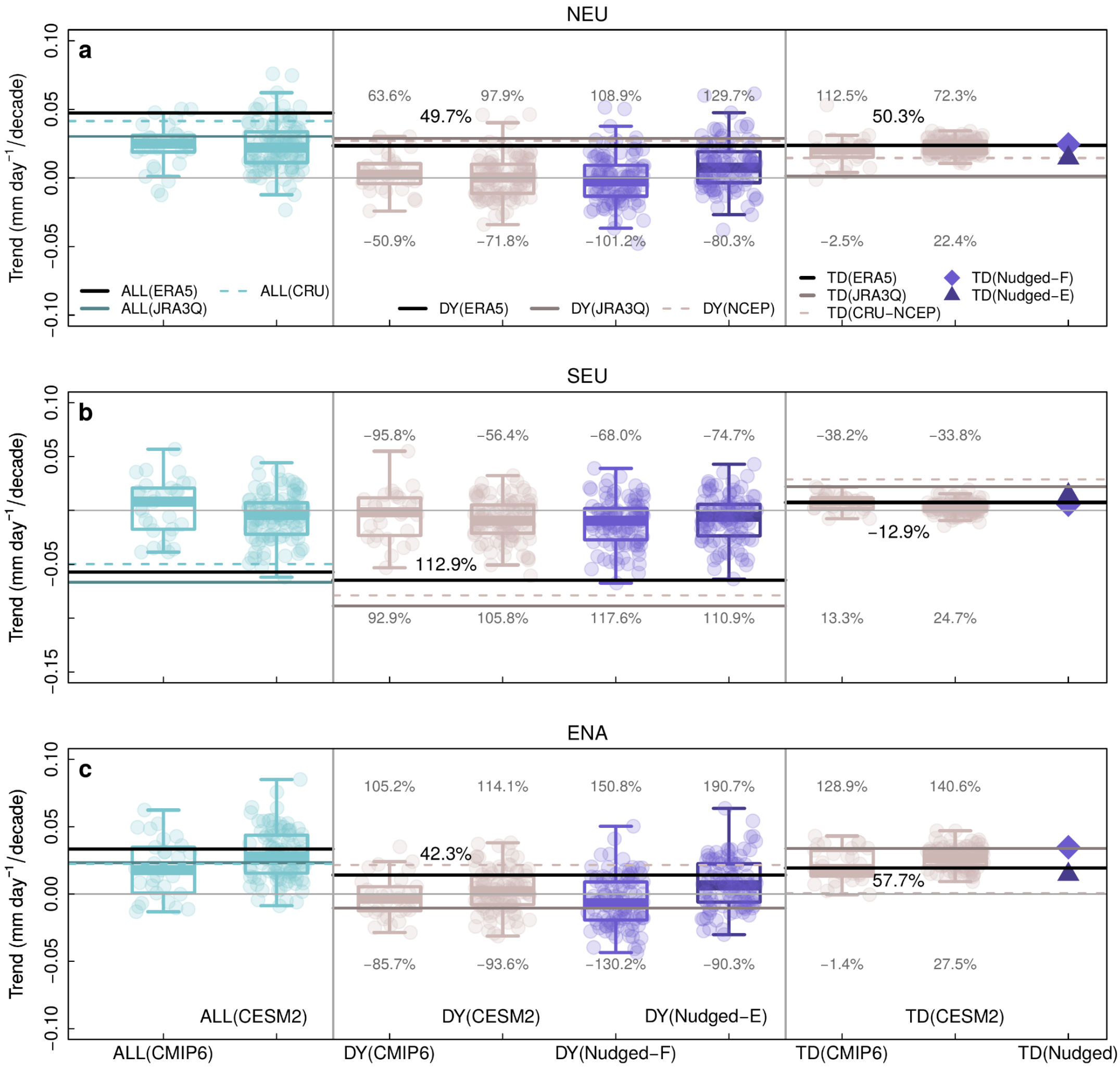

Fig. 2. Total, dynamic, and thermodynamic precipitation trends for winter precipitation. a, Winter precipitation trends (ALL) over the northern Europe (NEU) during 1950–2022 decomposed into dynamic (DY) and thermodynamic (TD) components (mm day^{-1} / decade). 'CMIP6' and 'CESM2', trends based on the elastic-net model from the CMIP6 and CESM2 ensembles. 'Nudged-F' denotes decomposed trends based on the free-nudging climate model experiments (Methods); 'Nudged-E' denotes decomposed trends from the nudged experiments derived with ERA5 winds. Horizontal lines represent trends from the ERA5, CRU, and JRA-3Q datasets. DY(NCEP) is estimated by using sea level pressure from the NCEP reanalysis, TD(CRU-NCEP) is then estimated by subtracting the DY(NCEP) from the ALL(CRU) precipitation trends. **b,c,** The same as **(a),** but for precipitation trends over southern Europe (SEU) and over eastern North America (ENA). Black numbers denote dynamic and thermodynamic contributions relative to total precipitation trends from the ERA5. Gray numbers represent dynamic and thermodynamic contributions from model simulations relative to total precipitation trends using ERA5 as the benchmark.

12. L194-196: "However, the 'most likely simulation' (Methods) fails to capture this shift in atmospheric dynamics over recent decades (Supplementary Fig. S7d-g), highlighting the inability of climate models to reproduce observed dynamic precipitation trends in Europe". Since the "most likely simulation" is defined based on the high correlation between the dynamic (or thermodynamic) precipitation components from models and the dynamic component derived from ERA5 reanalysis, does this statement mean that these two model realizations reproduce the precipitation

component reasonably with incorrect circulation background?

Reply: Yes, these two model realizations reproduce the precipitation component reasonably despite an inconsistent circulation background. Specifically, the 'most likely simulation' (now revised as 'best-matching simulation'), identified based on the highest correlation of dynamic precipitation trends between model simulations and ERA5 reanalysis, does not coincide with the simulation that best reproduces the large-scale circulation patterns (e.g., SLP background). We have clarified this point in the revised ***Physical drivers of decomposed precipitation trends*** section as follows:

However, the 'best-matching simulation' (Methods) captures little of the observed shift in atmospheric dynamics over recent decades (Supplementary Fig. 5). The pattern correlations (above 30°N) of *SLP* (*U500*) between CMIP6 and CESM2-LE simulations and ERA5 are 0.05±0.31 and 0.11±0.34 (0.00±0.20 and –0.01±0.27), respectively (Supplementary Fig. 7). Accordingly, the simulation that best reproduces dynamic precipitation trends does not correspond to the highest correlations in either *SLP* or *U500*. This indicates limitations of climate models to reproduce observed dynamic precipitation trends over Europe.

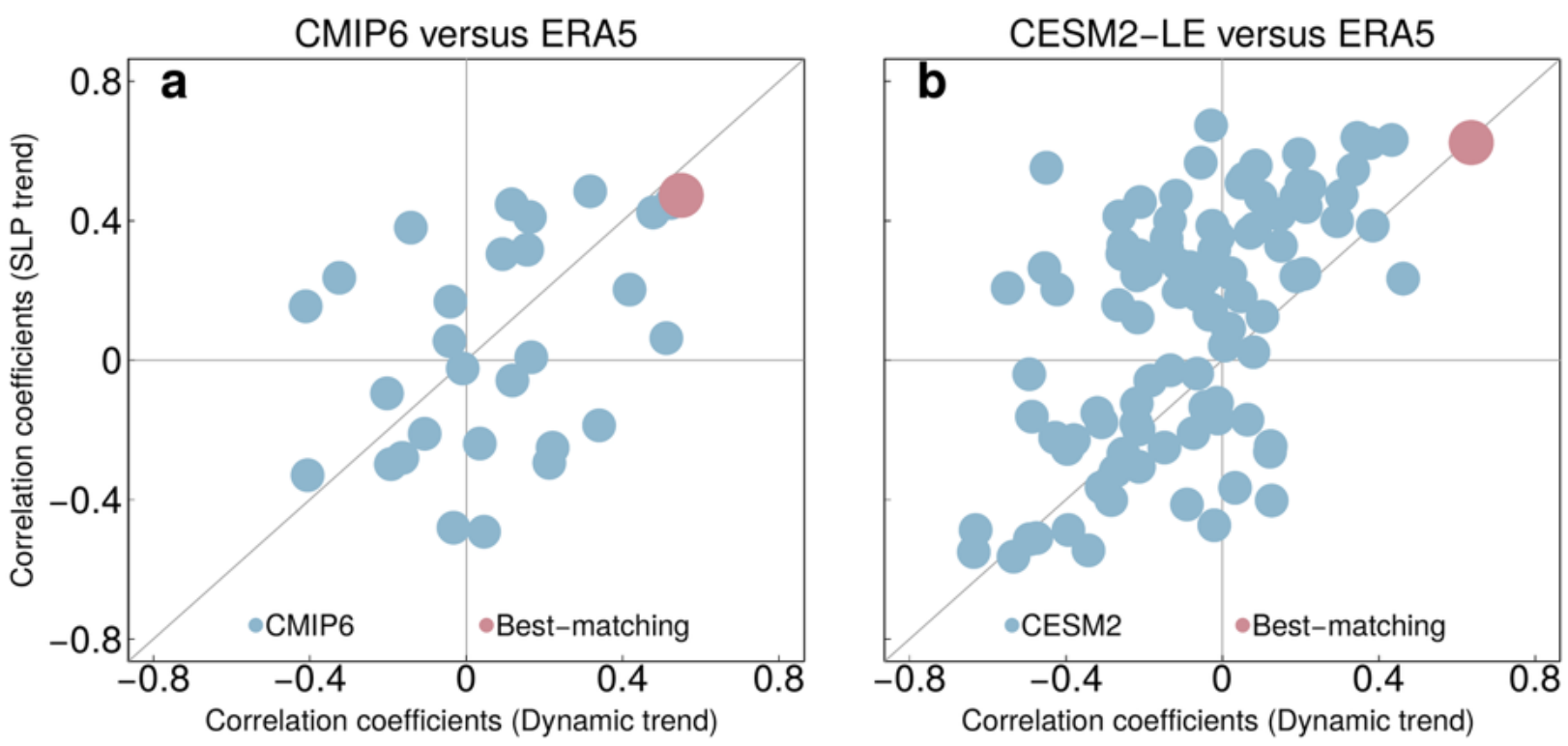

Supplementary Figure 7. The Pearson correlation coefficients of dynamic precipitation trend and dynamic indicator (sea level pressure, SLP) trend between climate model simulations and the ERA5 reanalysis. a, The correlation coefficients between the CMIP6 ensemble simulations and the ERA5 reanalysis. *x*-axis denotes the correlation of dynamic precipitation trend; *y*-axis denotes the correlation of SLP trend. **b**, The same as **a**, but correlation coefficients between the CESM2-LE and the ERA5.

13. L210-211: again, a quantitative comparison is appreciated.

Reply: Thanks very much for pointing out this. We have clarified the quantitative comparison in the revised ***Physical drivers of decomposed precipitation trends*** section as follow:

Despite modest spatial heterogeneity, the ability of climate models to reproduce trends in thermodynamic indicators (for example, *R*=0.33±0.13 between CMIP6 and ERA5 evapotranspiration patterns and *R*=0.40±0.06 between CESM2 and ERA5 patterns,

Supplementary Fig. 9) supports their ability to capture changes in observed thermodynamic precipitation.

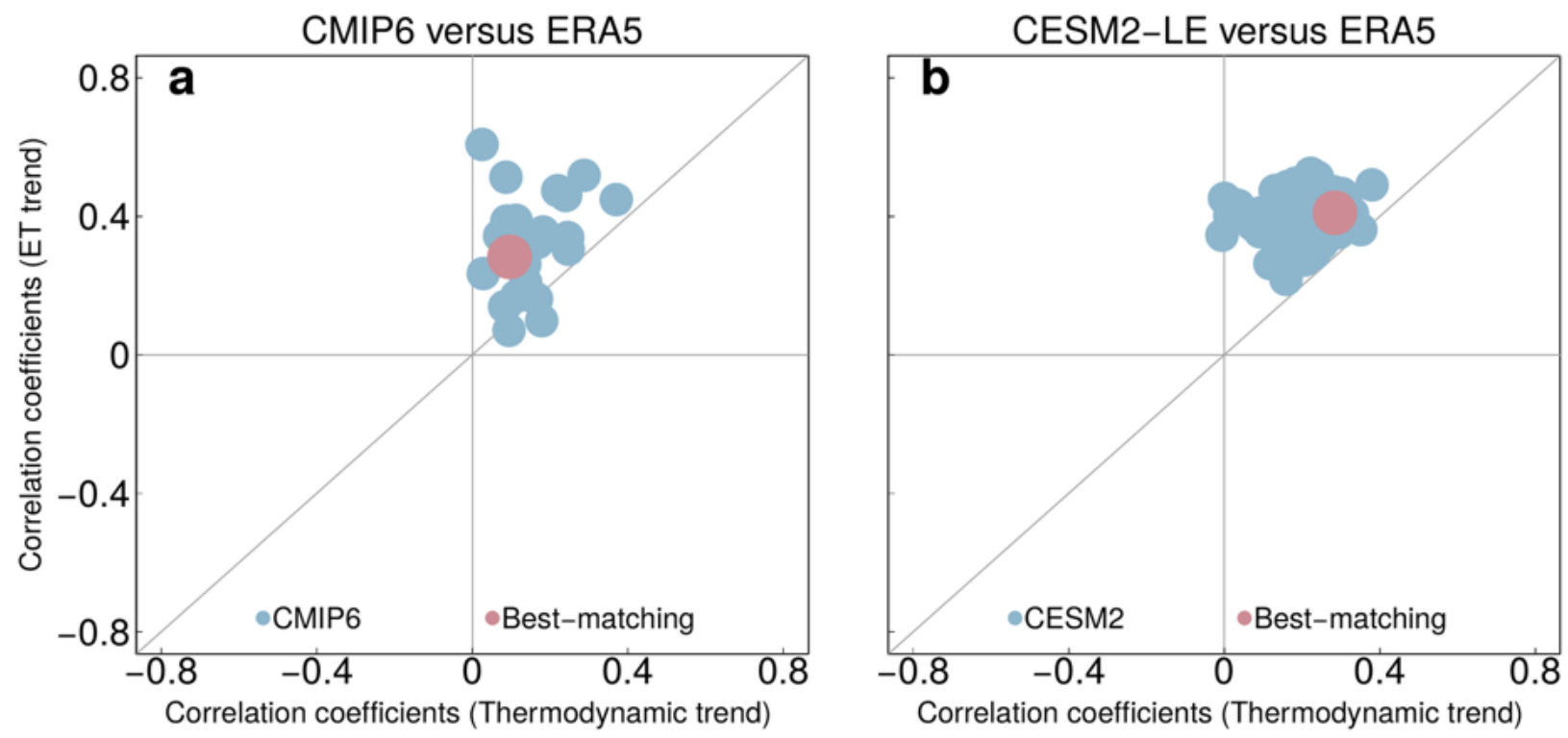

Supplementary Figure 9. The Pearson correlation coefficients of thermodynamic precipitation trend and thermodynamic indicator (evapotranspiration, ET) trend between climate model simulations and the ERA5 reanalysis. a, The correlation coefficients between the CMIP6 ensemble simulations and the ERA5 reanalysis. *x*-axis denotes the correlation of thermodynamic precipitation trend; *y*-axis denotes the correlation of ET trend. **b**, The same as **a**, but correlation coefficients between the CESM2-LE and the ERA5.

14. L238-239: even the ERA5 nudged simulation still failed in reproducing the observed dynamic precipitation trends, any explanation for this? I once hope that the ERA5 nudged simulation should be better than the freely coupled historical–SSP simulations in this regard.

Reply: The ERA5 nudged simulation robustly reproduces the observed total precipitation trends, thereby allowing an estimation of anthropogenically forced thermodynamic precipitation trends (Fig. 3h). However, its AMIP-style configuration is not directly consistent with the standard CESM2 model setup. This structural difference may introduce additional uncertainty when deriving forced dynamic precipitation trends (Fig. 3g) by subtracting forced thermodynamic trends from the CESM2 multi-member mean trends. Please refer to the response to **Major comment 2** for a thorough comparison and discussion.

15. L259: After "In reanalysis, dynamic trends are strongly negative across most grid cells", add "in SEU".

Reply: Done.

16. Figure 4: no discussion was given for Fig.4g,h,i in the text. In addition, the curves shown on Fig.4 are not clear.

Reply: Thanks for point this out. We have clarified Fig. 4g,h,i (now moving it to

Supplementary Fig. 14) in the revised ***Attribution to external forcing*** section and replot the figure as follows:

Total thermodynamic trends are partly attributable to external forcing (Supplementary Fig. 14g,i) and exceed the magnitude of the forced dynamic trends. The residual internal thermodynamic component (Supplementary Fig. 14h) shows no systematic regional signal, indicating that internal variability modulates the thermodynamic contribution at the grid-cell level without altering regional-scale trends. Note that the thermodynamic component in our framework is defined broadly to include contributions from evapotranspiration, sub-monthly transient eddies, and nonlinear interactions between dynamic and thermodynamic processes.

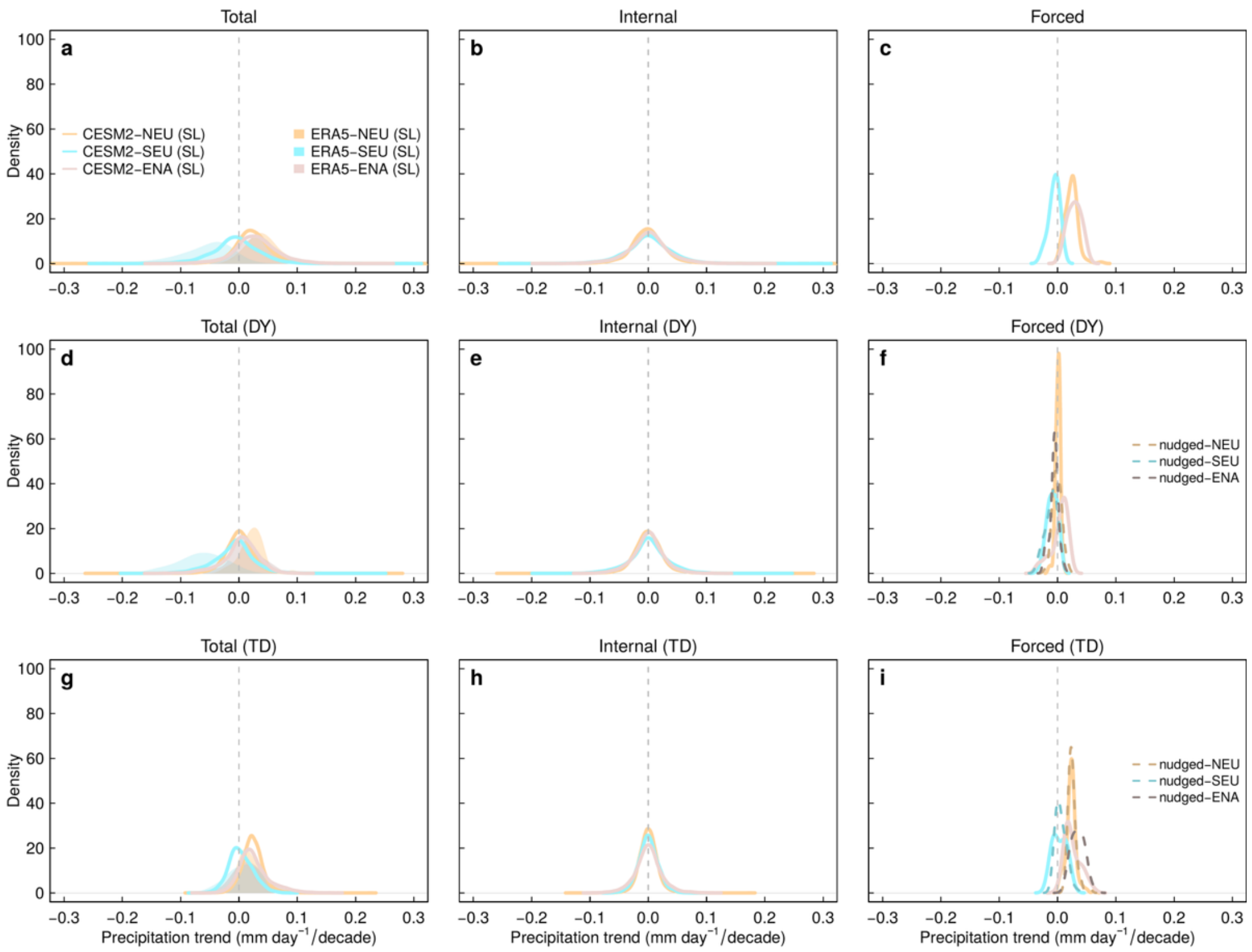

Supplementary Fig. 14. Density plots of precipitation trends decomposed into internal, forced, dynamic, and thermodynamic components for northern Europe (NEU), southern Europe (SEU), and eastern North America (ENA) during the period 1950–2022. a-c, Total, total internal, and total anthropogenic forced precipitation trends. **d-f,** Full dynamic (DY), internal dynamic (DY), and anthropogenic forced dynamic (DY) precipitation trends. **g-i,** Full thermodynamic (TD), internal thermodynamic (TD), and anthropogenic forced thermodynamic (TD) precipitation trends. Density curve estimation and decomposition methods are detailed in Methods. *x*-axis denotes trends and *y*-axis denotes number of grid points. The decomposition is based on the CESM2 large ensemble using statistical learning models and nudged climate model experiments. Because ERA5 reanalysis reflects a single realization, ideal separation into internal, forced, dynamic, and thermodynamic components is not possible. For comparison, polygon-based density plots are used to represent ERA5-derived results in panels **a,d,** and **g.**

17. L290: "our nudged climate model experiments offer a robust framework for decomposing winter precipitation trends into dynamic and thermodynamic components". Again, it would be better to clarify the differences to traditional dynamic and thermodynamic definitions.

Reply: Great suggestion to improve the manuscript. Please refer to the response to **Major comment 1** for a thorough comparison and discussion.

18. L331: "The projected decline in dynamic precipitation trends closely resembles the current ERA5 pattern, lending further support to our interpretation". I cannot understand why the resemblance of future projection to the current observation supports your interpretation here.

Reply: Sorry for the vague sentence. We have clarified this point in the revised ***Outlook*** (Last paragraph) section as follows:

The projected decline in dynamic precipitation trends over the SEU, also reported by Carruthers et al., (2025), closely resembles the present-day pattern in ERA5. This correspondence may arise either because internally driven variability in observations happens to resemble the projected forced response, and/or because climate models underestimate the magnitude of the forced dynamic contribution. In either case, improving the representation of large-scale circulation is critical for increasing confidence in regional precipitation projections.

Carruthers, James G., Hayley J. Fowler, Daniel Bannister, and Selma B. Guerreiro. Dynamical adjustment reveals spatial patterns of wetting and drying in European winter precipitation. Environmental Research Letters 20, no. 11 (2025): 114085.

Reviewer #2 Evaluations:
This manuscript compares winter precipitation trends from 1950-2022 in the Northern Hemisphere extra-tropics across observations, reanalysis, and earth system models. They focus on three regions where the trend is robustly significant: Eastern North America (getting wetter), Northern Europe (getting wetter), and Southern Europe (getting drier). On average, models predict similar patterns in the trend across these three regions as obs/reanalysis but underestimate the magnitude. To understand the source of this discrepancy the authors decompose the trend into combinations of forced and unforced and dynamic and thermodynamic components of the full precipitation trend.
The methods used in the decomposition are derived from past work (e.g., Sippel et al. 2019 for the statistical method used across all datasets including obs/reanalysis and Wehrli et al. 2018 for nudging CESM2 horizontal winds to various target states while keeping the forcing and land use fixed at piControl). The idea of using a statistical method like regularized linear regression across observations and models and validating it using a large ensemble is not entirely new (e.g., ForceSMIP) but has not yet been published for the precipitation trends in focus here. Thus to my knowledge the analysis provided here is novel. The findings are important (and some surprising, such as the significant spatial heterogeneity of the thermodynamic component) and seems worthy of publishing in Nature since it follows up on work that was recently published in the same journal (Vicente-Serrano et al. 2025).
However, there is some overlap in the analysis of the Southern Europe region in this study and recently published work (Doane-Solomon et al. 2025). Interestingly, the conclusions drawn are not the same. In this manuscript the dynamic component in the Mediterranean region is poorly represented by models while the residual (thermodynamic) component is better represented at least with ERA5. In Doane-Solomon et al. 2025 the dynamic component is well represented by models but not the residual. This may be due to differences in the analysis time period and/or differences in the way dynamical adjustment is made. I encourage the authors to investigate this further.
In addition to the omission of relevant literature, there are several other concerns that I would like to see the authors address if this manuscript were to be published. I detail them below. Overall, I can see this manuscript being fit for publication if these concerns are addressed.

Osamu Miyawaki
Dec 3, 2025.

Reply: Dear Dr. Miyawaki, we appreciate your insightful feedbacks. As your suggestions, we have investigated the sensitivity of our decomposition results to different time periods, and have included another reanalysis dataset – JRA3Q for comparison. Moreover, we have analyzed the dynamic, thermodynamic, and interaction

effects using another two approaches – moisture budget diagnostic and omega 500hPa indicator – in comparison to our statistical learning approach. All your concerns have also been addressed, and we hope you find this revised manuscript suitable for publication.

1. The manuscript focuses on 1950–2022 when Vicente-Serrano et al. (2025) show Mediterranean precipitation trends are sensitive to the considered time period. Doane-Solomon (2025) confirms this, showing trends from 1979–2022 are not statistically significant over the Mediterranean. How sensitive are the conclusions to the time period chosen? Can the authors better motivate why 1950-–2022 was chosen?

Reply: We acknowledge that the relative contributions of dynamic and thermodynamic processes vary across different time periods, particularly over the Mediterranean. Given the large uncertainty in precipitation trends, we therefore focus on longer records to obtain decomposed contributions with greater confidence. Shorter periods (for example, 1980–2022) are more strongly influenced by internal variability, which can obscure the emergence of anthropogenic signals. Please refer to details in the revised ***Outlook*** (Fourth paragraph) section as follows:

We find significant drying for 1950–2022, while shorter periods show weaker or no trends (Supplementary Fig. 3). Similar circulation-associated drying has been reported for 1921–2015 (Guo et al., 2019) and 1959–2021 (Seager et al., 2024), yet is not reproduced by CMIP6 models. In contrast, Doane-Solomon et al. (2025) show that SEU precipitation trends during 1980–2022 are insignificant, with a weak dynamic component that is well captured by model ensembles. This period-dependence demonstrates strong internal variability rather than directly constraining the forced component's magnitude (Supplementary Fig. 19). Strong multi-decadal NAO oscillations may overwhelm a moderate forced signal (Supplementary Fig. 20), explaining why only certain sufficiently long and recent periods reveal the emerging anthropogenic contribution.

Guo, R., Deser, C., Terray, L., & Lehner, F. (2019). Human influence on winter precipitation trends (1921–2015) over North America and Eurasia revealed by dynamical adjustment. Geophysical Research Letters, 46(6), 3426-3434.

Seager, R., Wu, Y., Cherchi, A., Simpson, I. R., Osborn, T. J., Kushnir, Y. et al. (2024). Recent and near-term future changes in impacts-relevant seasonal hydroclimate in the world's Mediterranean climate regions. International Journal of Climatology, 44(11), 3792-3820.

Doane-Solomon, R., Woollings, T., & Simpson, I. R. (2025). Dynamic Contributions to Recent Observed Wintertime Precipitation Trends in Mediterranean-Type Climate Regions. Geophysical Research Letters, 52(12).

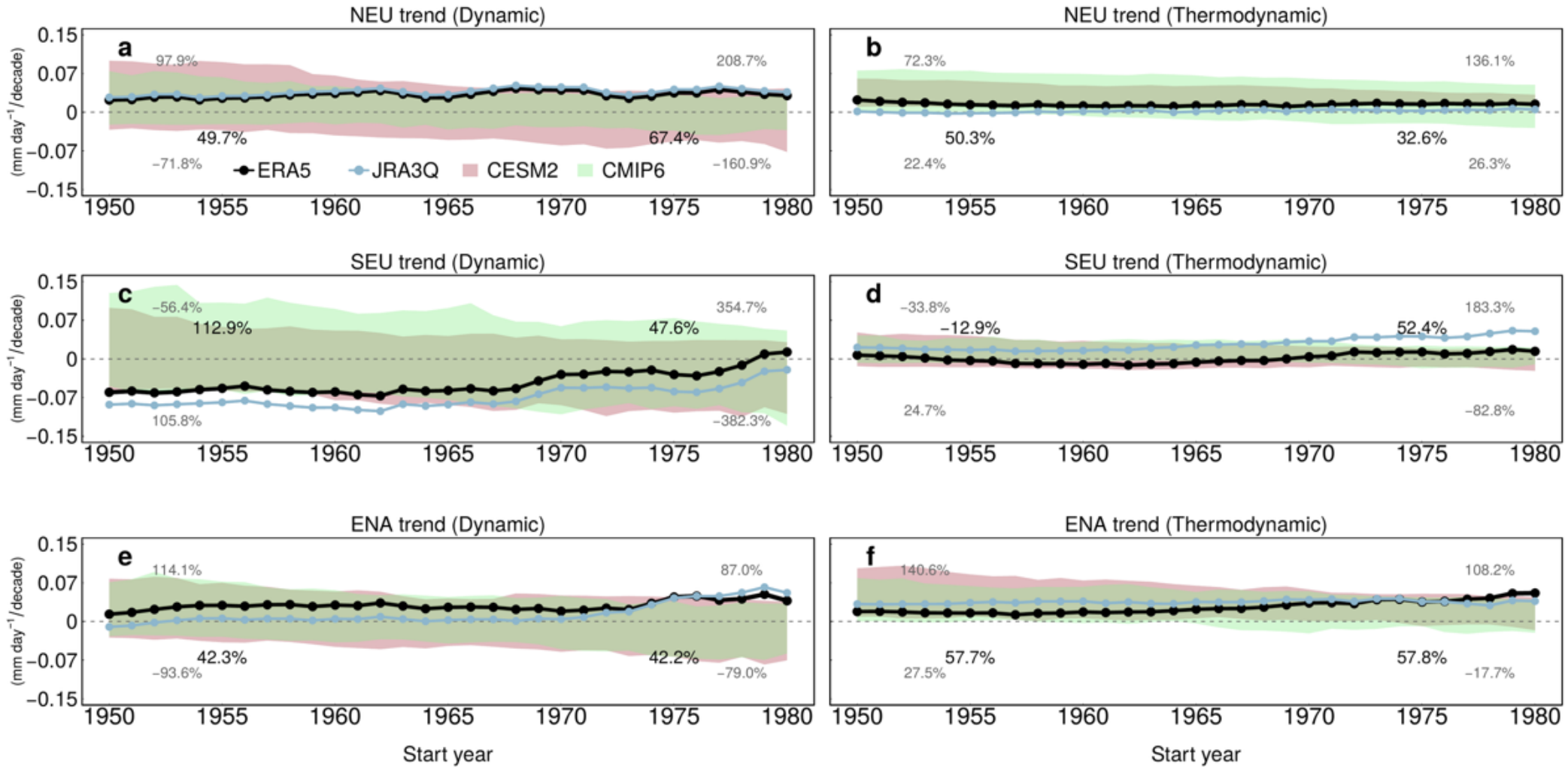

Supplementary Figure 19. The magnitude of linear dynamic and thermodynamic precipitation trends ending in 2022 as a function of start year. a,**b**, The magnitudes of linear dynamic (**a**) and thermodynamic (**b**) precipitation trends ending in 2022 as a function of start year from the ERA5, JRA-3Q, CMIP6 and CESM2 ensembles over northern Europe (NEU). **c**,**d**, The same as **a**,**b**, but for dynamic (**c**) and thermodynamic (**d**) precipitation trends over southern Europe (SEU). **e**,**f**, The same as **a**,**b**, but for dynamic (**c**) and thermodynamic (**d**) precipitation trends over eastern North America (ENA). The grey numbers show dynamic (**a**,**c**,**e**) versus thermodynamic (**b**,**d**,**f**) contributions (for the start years of 1950 and 1980) from CESM2 large ensemble relative to total precipitation trends using ERA5 as the benchmark; the black numbers show respective contributions of decomposed dynamic versus thermodynamic trends estimated from the ERA5 reanalysis.

2. There are several statements in the manuscript that aren't sufficiently supported by the presented data. Line 164 states "thermodynamic trends derived from climate models closely match those from reanalysis" but the thermodynamic component of the precipitation response over the Mediterranean using the CRU-NCEP dataset is outside of the climate model distribution. Sensitivity analysis with other reanalysis datasets such as MERRA2 and JRA3Q would be valuable to better quantify the obs/reanalysis spread. Line 194-195 states "the most likely simulation fails to capture this shift in atmospheric dynamics over recent decades" but Supplementary Fig. S7d-g largely show similar large-scale patterns of SLP and u500 as in ERA5. The difference between reanalysis and models seem more subtle than stated in the manuscript, i.e. the issues involve small differences in the magnitude of the trends (CESM2 72 underestimates the magnitude of trends over the North Atlantic) or the exact position of the dipole (e.g. FGOALS dipole is too ear to the east). Condensing this as "fails to capture this shift" seems misleading.

Reply: We agree that introducing other reanalysis datasets is helpful to quantify the obs/reanalysis spread, therefore include the JRA-3Q reanalysis as a comparison, which spans from 1950. The fact that the thermodynamic component of the Mediterranean precipitation trend derived from the CRU-NCEP dataset lies outside the model range, may partly reflect inconsistencies between CRU and NCEP products. Using JRA-3Q

and ERA5, we find that the CMIP6 spread encompasses the thermodynamic component over SEU. To better clarify our decomposition results from reanalysis and climate model simulations, we demonstrate the percentile contributions in the revised Fig. 2. In addition, we have toned down qualitative statements such as 'fails to capture this shift' and replaced them with quantitative descriptions in the revised ***Physical drivers of decomposed precipitation trends*** section as follows:

However, the 'best-matching simulation' (Methods) captures little of the observed shift in atmospheric dynamics over recent decades (Supplementary Fig. 5). The pattern correlations (above 30°N) of *SLP* (*U500*) between CMIP6 and CESM2-LE simulations and ERA5 are 0.05±0.31 and 0.11±0.34 (0.00±0.20 and –0.01±0.27), respectively (Supplementary Fig. 7). Accordingly, the simulation that best reproduces dynamic precipitation trends does not correspond to the highest correlations in either *SLP* or *U500*. This indicates limitations of climate models to reproduce observed dynamic precipitation trends in Europe.

Despite modest spatial heterogeneity, the ability of climate models to reproduce trends in thermodynamic indicators (for example, R=0.33±0.13 between CMIP6 and ERA5 evapotranspiration patterns and R=0.40±0.06 between CESM2 and ERA5 patterns, Supplementary Fig. 9) supports their ability to capture changes in observed thermodynamic precipitation.

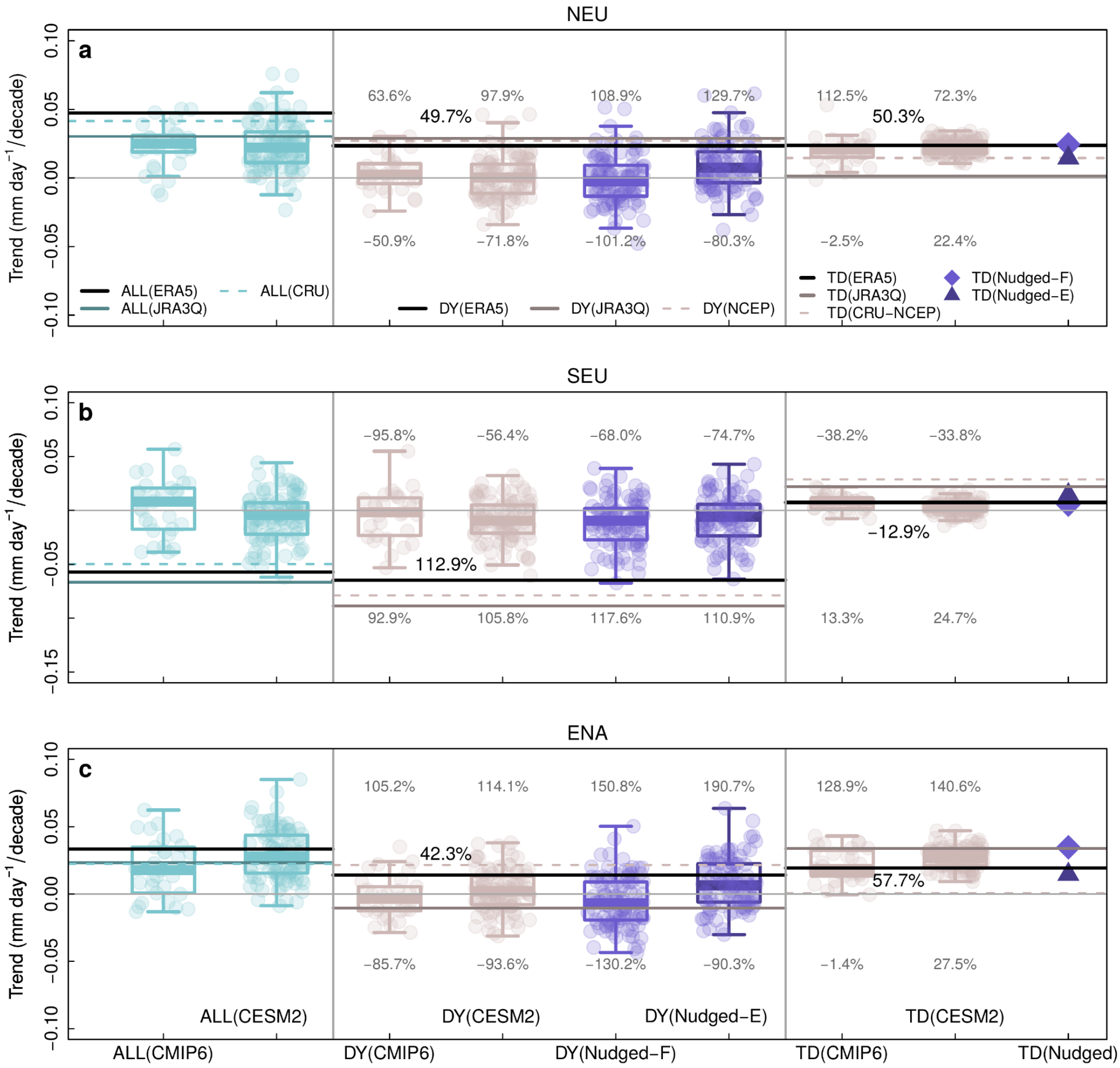

Fig. 2. Total, dynamic, and thermodynamic precipitation trends for winter (DJF) precipitation. a, Winter precipitation trends (ALL) over the northern Europe (NEU) during 1950–2022 decomposed into dynamic (DY) and thermodynamic (TD) components (mm day^{-1} / decade). 'CMIP6' and 'CESM2', trends based on the elastic-net model from the CMIP6 and CESM2 ensembles. 'Nudged-F' denotes decomposed trends based on the free-nudging climate model experiments (Methods); 'Nudged-E' denotes decomposed trends from the nudged experiments derived with ERA5 winds. Horizontal lines represent trends from the ERA5, CRU, and JRA-3Q datasets. DY(NCEP) is estimated by using sea level pressure from the NCEP reanalysis, TD(CRU-NCEP) is then estimated by subtracting the DY(NCEP) from the ALL(CRU) precipitation trends. **b,c,** The same as **(a),** but for precipitation trends over southern Europe (SEU) and over eastern North America (ENA). Black numbers denote dynamic and thermodynamic contributions relative to total precipitation trends from the ERA5. Gray numbers represent dynamic and thermodynamic contributions from model simulations relative to total precipitation trends using ERA5 as the benchmark.

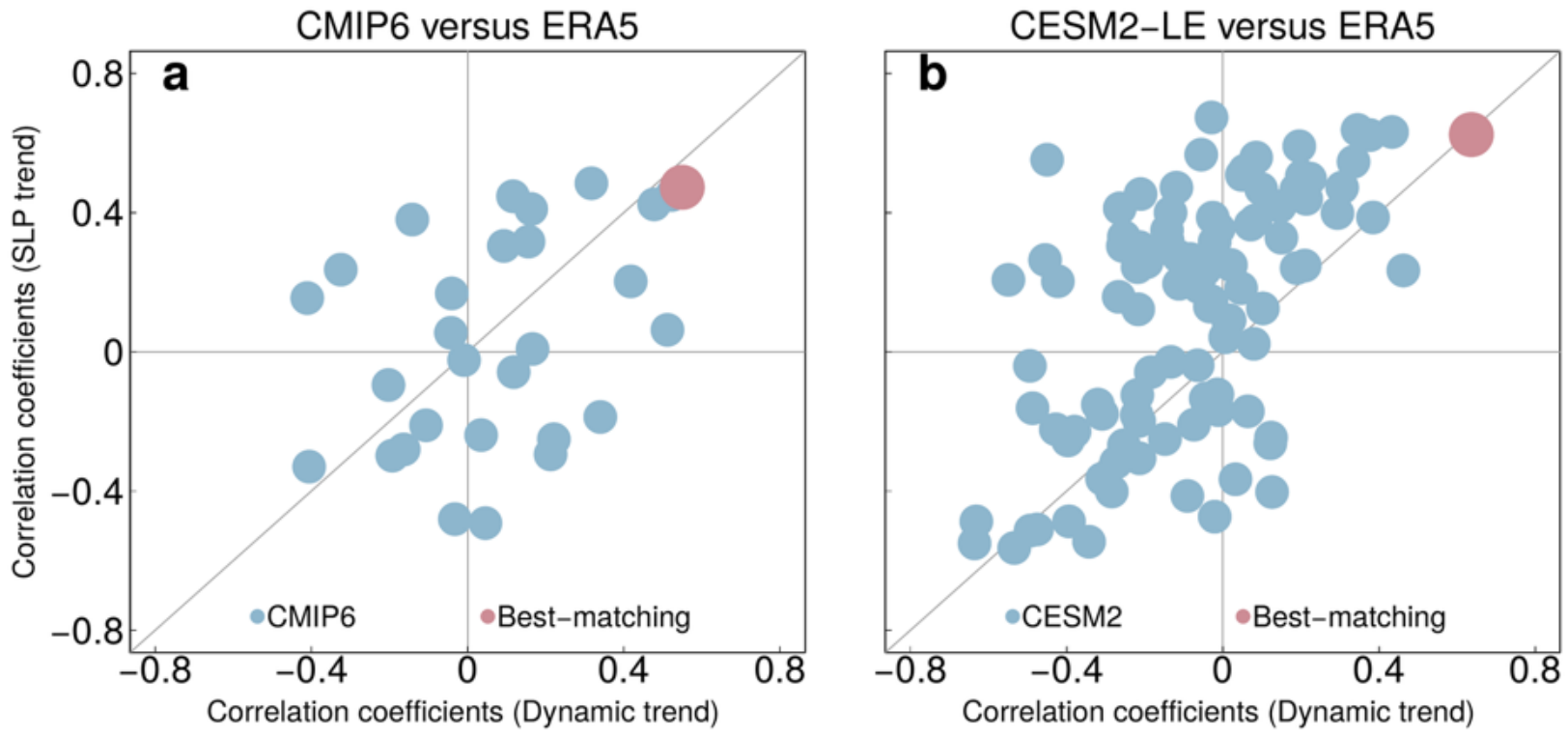

Supplementary Figure 7. The Pearson correlation of dynamic precipitation trend and dynamic indicator (sea level pressure) trend between climate model simulations and the ERA5 reanalysis. a, The correlation coefficients between the CMIP6 ensemble simulations and the ERA5 reanalysis. *x*-axis denotes the correlation of dynamic precipitation trend; *y*-axis denotes the correlation of sea level pressure (SLP) trend. **b**, The same as **a**, but correlation between the CESM2-LE and the ERA5.

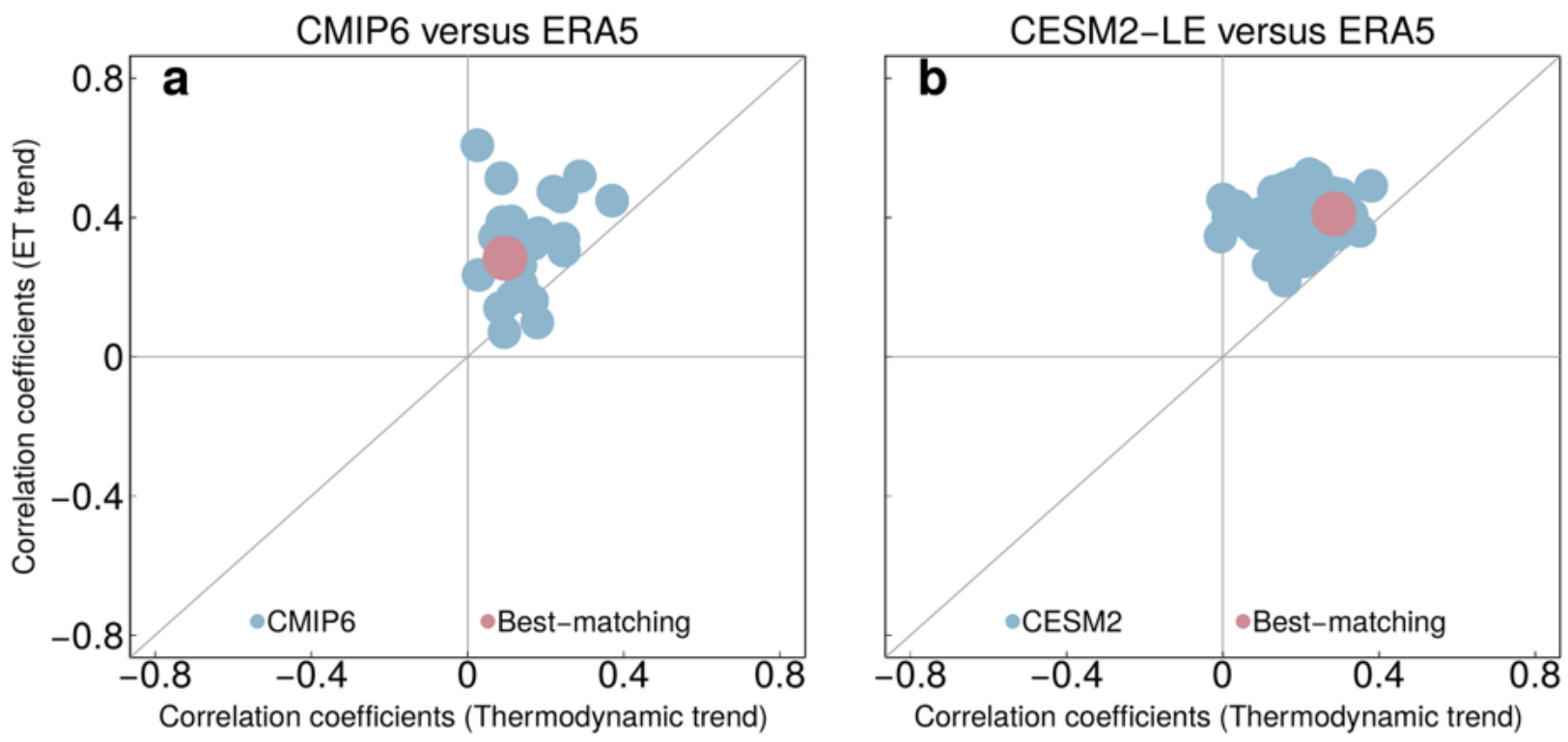

Supplementary Figure 9. The Pearson correlation of thermodynamic precipitation trend and thermodynamic indicator (evapotranspiration) trend between climate model simulations and the ERA5 reanalysis. a, The correlation coefficients between the CMIP6 ensemble simulations and the ERA5 reanalysis. *x*-axis denotes the correlation of thermodynamic precipitation trend; *y*-axis denotes the correlation of evapotranspiration (ET) trend. **b**, The same as **a**, but correlation between the CESM2-LE and the ERA5.

3. The assumptions involved in the decomposition (line 403-406) limit the interpretability of the dynamic/thermodynamic decomposition. Comparing the decomposed terms with the maps of SLP, u500, huss, and ET is a useful start to validating the decomposition but the presented analysis is largely qualitative and hand-wavy (e.g. line 199-200). Adding to this concern is that some datasets like CRU show significant anti-correlation between the dynamic and thermodynamic components (Supplementary Figure 6). Unless there is a physical explanation for this anti-correlation I suspect higher-order terms (interaction between dynamics and thermodynamics) may be important. Thus assuming the residual as a purely

"thermodynamic" contribution is misleading, especially so to a broad audience such as Nature's (e.g., see the Discussion section in Doane-Solomon et al. 2025 for a more cautious perspective on this).

Reply: We have expanded analysis of dynamic and thermodynamic indicators (*SLP*, *U500*, *HUSS*, and *ET*) with quantitative descriptions, please refer to **response 2** and Supplementary Figs. 7 and 9. The apparent anti-correlation between the dynamic and thermodynamic components from CRU dataset may arise from inconsistencies between the CRU and NCEP product. To address this issue, we include the JRA-3Q reanalysis spanning from 1950 as you suggested. The anti-correlation problem has been well mitigated, please see the revised Supplementary Fig. 4c-d. We further clarify that the thermodynamic influence is defined to encompass all non-circulation factors.

To fully address your concern, we additionally employ two independent approaches to decompose dynamic and thermodynamic contributions: 1) based on 37 pressure levels from ERA5 and 45 pressure levels from JRA-3Q, using a monthly-scale moisture budget decomposition that explicitly separates vertical and horizontal moisture advection (Chou and Lan, 2012; Jiang et al., 2023); 2) a daily-scale decomposition based on omega at 500hPa level of ERA5 and JRA-3Q following Emori and Brown (2005). We find that the nonlinear interactions are small; however, sub-monthly transient eddies, which is attributed to thermodynamic influence in this study can be substantial. Details can be found in the revised ***Attribution to external forcing*** and ***Outlook*** (Second paragraph) sections as follows:

Attribution to external forcing: Total thermodynamic trends are partly attributable to external forcing (Supplementary Fig. 14g,i) and exceed the magnitude of the forced dynamic trends. The residual internal thermodynamic component (Supplementary Fig. 14h) shows no systematic regional signal, indicating that internal variability modulates the thermodynamic contribution at the grid-cell level without altering regional-scale trends. Note that the thermodynamic component in our framework is defined broadly to include contributions from evapotranspiration, sub-monthly transient eddies, and nonlinear interactions between dynamic and thermodynamic processes.

Outlook: In this study, thermodynamic influence is defined broadly to encompass all non-circulation factors, such as changes in lapse rate, land–sea thermal contrast, evapotranspiration, humidity, transient eddies, and nonlinear effects. This definition is reflected in the thermodynamic contributions identified by the moisture budget analysis (Chou and Lan, 2012; Jiang et al., 2023). This moisture budget method typically restricts thermodynamic effects to temperature- and humidity-related changes in vertical and horizontal moisture advection, thus yielding different thermodynamic partitioning relative to our statistical learning approach (Supplementary Figs. 16–17). By contrast, dynamic contributions to total precipitation trends are generally consistent

across the two methods, supporting the robustness of the decomposition. To provide an additional comparison, we also apply an empirical binning approach using 500hPa vertical velocity as the proxy for the strength of dynamic disturbance (Emori and Brown, 2005). Although the magnitude of the dynamic contribution obtained from this method is systematically smaller, the sign is consistent with results from the statistical learning and moisture budget. This further confirms that the circulation-wetting in NEU/ENA and circulation-drying in SEU is not an artifact.

Chou, C., & Lan, C. W. (2012). Changes in the annual range of precipitation under global warming. Journal of Climate, 25(1), 222-235.

Jiang, J., Zhou, T., Qian, Y., et al. (2023). Precipitation regime changes in High Mountain Asia driven by cleaner air. Nature, 623(7987), 544-549.

Emori, S., & Brown, S. J. (2005). Dynamic and thermodynamic changes in mean and extreme precipitation under changed climate. Geophysical Research Letters, 32(17).

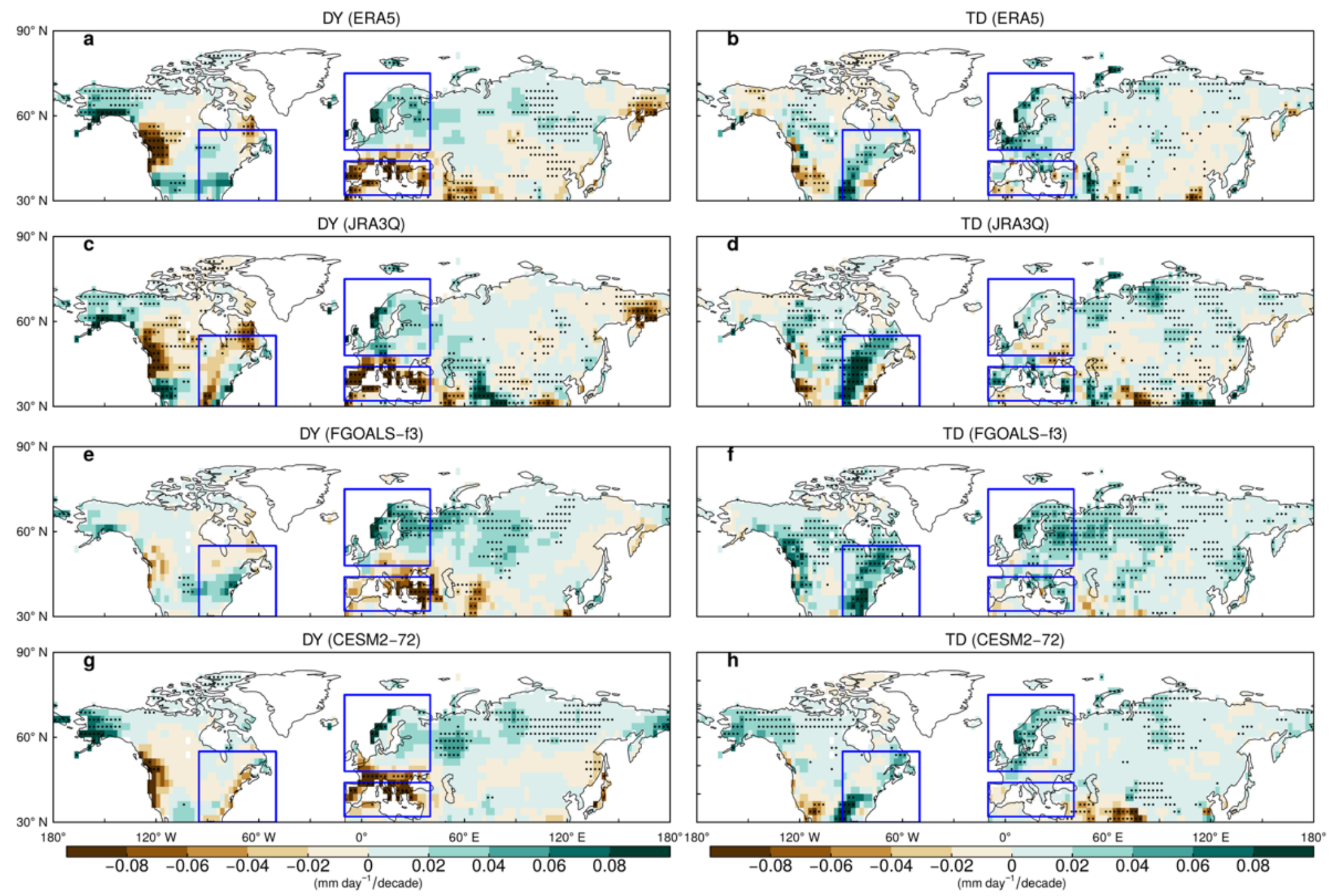

Supplementary Figure 4. Spatial maps of the dynamic and thermodynamic winter precipitation trends in observations and model simulations. a-d, The dynamic (DY, **a,c**) and thermodynamic (TD, **b,d**) winter precipitation changes using the elastic-net model from the ERA5 and JRA-Q datasets. **e,f,** The same as **a,b**, but the decomposed DY and TD trends are from the model in CMIP6 ensemble which shows the highest correlation coefficients towards the decomposed trends from the ERA5. **g,h,** The same as **e,f,** but from the member in CESM2 large ensemble. The dot stippling represents significant trends at the 0.05 level (p-value < 0.05).

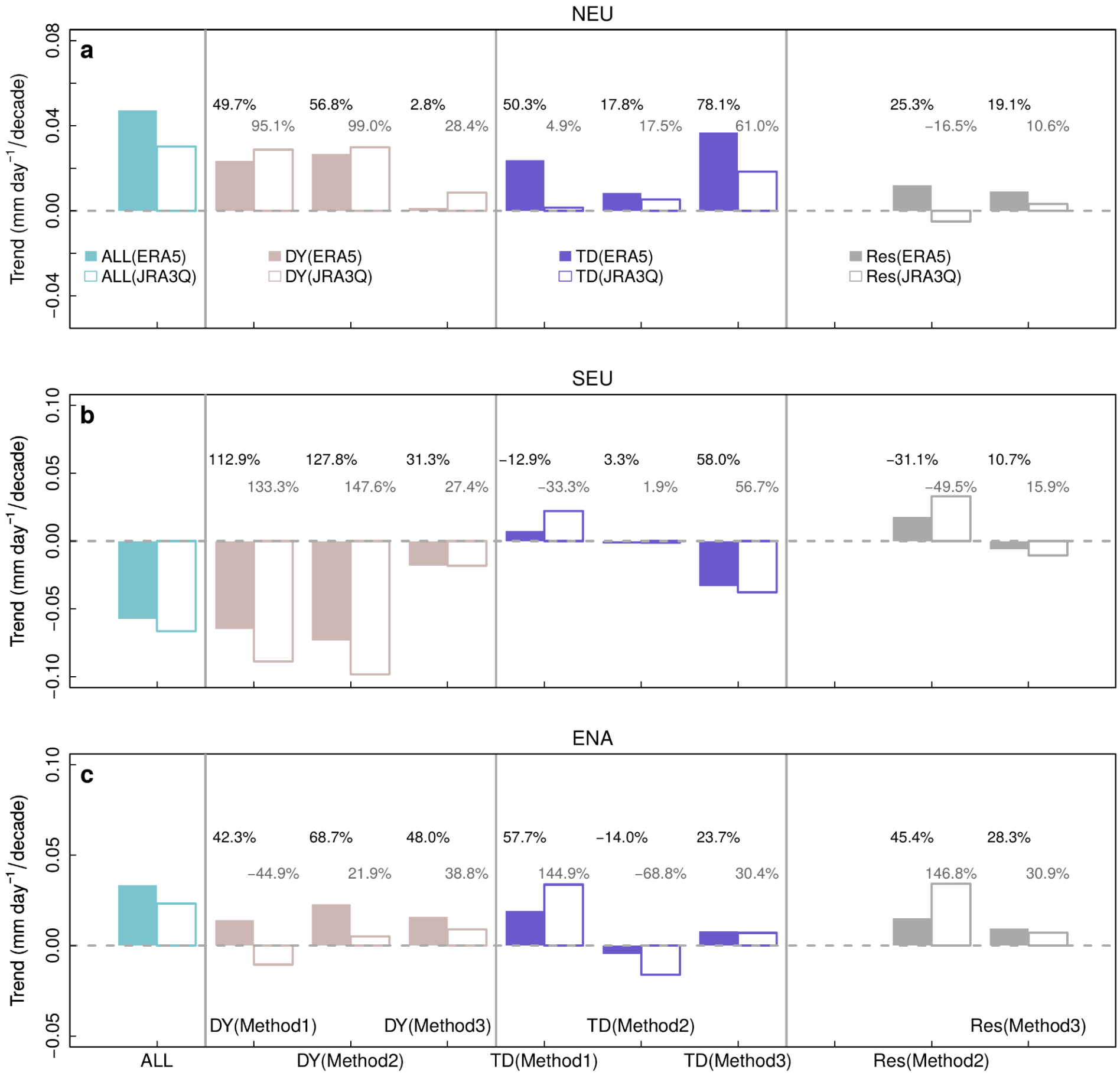

Supplementary Figure 16. Dynamic and thermodynamic contribution to winter precipitation trends. a, Winter precipitation trends over the northern Europe (NEU) during 1950–2022 (ALL) decomposed into dynamic (DY), thermodynamic (TD), and residual (Res) components using ERA5 and JRA-3Q reanalysis datasets. Method 1 represents the decomposed results based on the elastic-net model; Method 2 represents results based on the moisture budget analysis (Methods); Method 3 represents results using daily mean 500 hPa vertical velocity as the proxy of dynamic disturbance (Methods). **b,c,** The same as (**a**), but for total and decomposed trends over southern Europe (SEU) and over eastern North America (ENA). The black numbers represent contributions from ERA5 dataset and the grey numbers represent contributions from JRA-3Q dataset.

4. It would be nice to see how the methods presented here compare to even simpler methods like in Emori and Brown (2005). In that framework they find the covariance between dynamic and thermodynamic contributions are weak. If the dynamic and thermodynamic components as derived using Emori and Brown (2005) are similar to the methods used here, it may help alleviate my concerns about interpretability above.

Reply: We have included the decomposition method by Emori and Brown (2005) as a comparison in the revised manuscript. The decomposed dynamic and thermodynamic contributions vary across different approaches. The magnitude of dynamic precipitation trends is the largest in the moisture budget analysis, intermediate in the statistical learning method, and smallest when inferred from the omega 500hPa (ω500) method. This spread primarily reflects methodological differences in how dynamical processes are represented, and we have provided explanations regarding these comparisons in the

revised manuscript. The moisture budget analysis explicitly accounts for both horizontal (u, v) and vertical (ω) moisture advection, whereas the statistical learning framework emphasizes horizontal dynamic contributions. The substantially larger dynamic component inferred from the statistical learning approach relative to the ω500 method further suggests that, at the monthly scale, horizontal moisture convergence and divergence play a more prominent role than vertical moisture advection. We repeated the decomposition using a range of bin widths (10–50 pa s^{-1}), yielding qualitatively similar outcomes. A more detailed discussion of the methodological spread in the decomposed dynamic and thermodynamic trends is provided in **response 3**. The corresponding spatial patterns derived from the moisture budget analysis and the Emori and Brown (2005) framework are as follows:

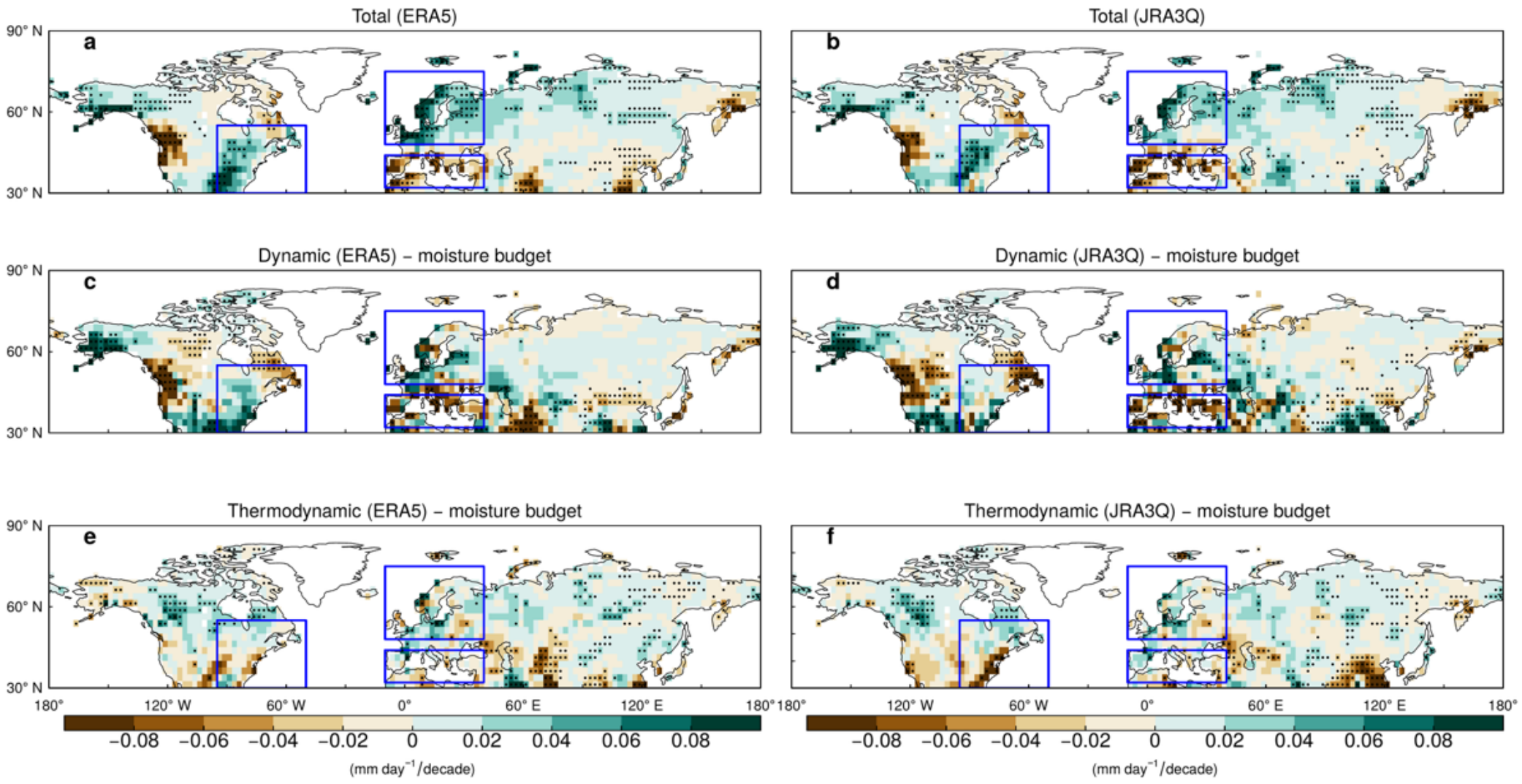

Supplementary Figure 17. Dynamic and thermodynamic winter precipitation trends based on moisture budget diagnostics. a,**c**,**e**, The total (**a**), dynamic (**c**), and thermodynamic (**e**) winter precipitation trends based on moisture budget diagnostics from the ERA5 reanalysis dataset. Note that the dynamic and thermodynamic trends include respective contributions from both horizontal and vertical moisture advection. **b**,**d**,**f**, The same as **a**,**c**,**e**, but from the JRA-3Q reanalysis dataset. The dot stippling represents significant trends at the 0.05 level (*p*-value < 0.5).

5. It is not clear to me why a single-member multi-model CMIP6 ensemble is included here. This is not sufficiently motivated in the manuscript. A seemingly more relevant ensemble is the subset of models that run single-model initial-condition large ensembles (SMILE). This work looks at one such dataset (CESM2-LE) but there are several others in the CMIP6 family forced with the SSP370 scenario (e.g., see Maher et al. 2023). Doane-Solomon et al. (2025) consider multiple SMILE ensembles and show the forced component of the precipitation response in the Mediterranean differ in sign across SMILEs. This is a more insightful quantification of model uncertainty of the forced response than can be obtained from a multi-model ensemble of the r1i1p1f1

run.

Reply: Thanks for this insightful comment. Climate models differ in their structural configurations and representations of physical processes, which can lead to model-specific biases. Training the statistical regression model on a multi-model ensemble helps mitigate such biases by leveraging the spread across models, an approach that has been adopted in previous studies (Singh et al., 2023; Egli et al., 2024). We have discussed this point in the revised ***Outlook*** (First paragraph) section as follows:

Moreover, the statistical decomposition results are largely insensitive to the choice of climate model ensemble. In addition to the multi-member large ensemble (CESM2-LE), we further employ a multi-model ensemble based on the first realization from each model for comparison. This strategy captures a broader range of process representations across climate models and facilitates the derivation of a more generalized statistical framework, consistent with previous studies (Singh et al., 2023; Egli et al., 2024). The resulting spread further suggests that inter-model differences are comparable to internal variability. Future work incorporating multiple large ensembles from different climate models is needed to disentangle individual model responses and underlying processes (Maher et al., 2023).

Singh, J., Sippel, S., & Fischer, E. M. (2023). Circulation dampened heat extremes intensification over the Midwest USA and amplified over Western Europe. Communications Earth & Environment, 4(1), 432.

Egli, M., Humphrey, V., Sippel, S., & Knutti, R. (2024). A distinct role for aerosol and GHG forcing in historical CMIP6 evapotranspiration trends. Earth's Future, 12(10), e2024EF004973.

Maher, N., Wills, R. C. J., DiNezio, P., Klavans, J., Milinski, S., Sanchez, S. C. et al. (2023). The future of the El Niño–Southern Oscillation: using large ensembles to illuminate time-varying responses and inter-model differences. Earth System Dynamics, 14(2), 413-431.

6. The manuscript misses some key references/comparisons to relevant work, e.g. Seager et al. (2024), Doane-Solomon et al. (2025).

Reply: We have cited these related references in the revised manuscript; and compared them with our results in the revised ***Outlook*** (Fourth and last paragraphs) Section as follows:

The detectability of such a signal depends critically on the period examined. We find significant drying for 1950–2022, while shorter periods show weaker or no trends (Supplementary Fig. 3). Similar circulation-associated drying has been reported for 1921–2015 (Guo et al., 2019) and 1959–2021 (Seager et al., 2024), yet is not reproduced by CMIP6 models. In contrast, Doane-Solomon et al. (2025) show that SEU precipitation trends during 1980–2022 are insignificant, with a weak dynamic component that is well captured by model ensembles. This period-dependence demonstrates strong internal variability rather than directly constraining the forced

component's magnitude (Supplementary Fig. 19). Strong multi-decadal NAO oscillations may overwhelm a moderate forced signal (Supplementary Fig. 20), explaining why only certain sufficiently long and recent periods reveal the emerging anthropogenic contribution.

Looking ahead, projections under increasing anthropogenic emissions imply a continued weakening of circulation over the Mediterranean, manifested as a strengthened signal-to-noise ratio[65] of sea level pressure trends (Supplementary Fig. 21). The projected decline in dynamic precipitation trends over the SEU, also reported by Carruthers et al., (2025), closely resembles the present-day pattern in ERA5. This correspondence may arise either because internally driven variability in observations happens to resemble the projected forced response, and/or because climate models underestimate the magnitude of the forced dynamic contribution. In either case, improving the representation of large-scale circulation is critical for increasing confidence in regional precipitation projections.

Reviewer #3 Evaluations:

Previous studies have shown that climate models tend to underestimate North Atlantic atmospheric circulation trends, including associated precipitation trends in North America and Europe, with observed trends being at the edge of the model spread. This mismatch is due to either 1) the models underestimating the forced trend or 2) the models underestimating internal variability, or 3) an unusually large realization of internal variability in the observed record. This paper investigates this issue in more detail by using two novel methods to distinguish between thermodynamic and dynamic parts of observed circulation and precipitation trends. The two methods, one based on nudged climate model simulations and the second on an advanced linear regression model, are novel, and provide some interesting results. However, the fundamental flaw of the paper is that the title and abstract grossly overstate the conclusions presented in the main paper. This is further explained in my main comment.

Reply: We appreciate the reviewer's feedback and suggestions; we have revised the **Title** and **Abstract**. All your concerns have been carefully addressed in the revised manuscript; and we hope you find it suitable for publication.

Main comment:

1. The title of the paper claims that 'human emissions' have led to strong circulation changes that have led to strong midlatitude winter precipitation trends. In addition, the abstract claims that models 'underestimate the amplitude of the forced dynamic contribution'. However, such strong statements are not supported by the main body of the paper. These statements are based on the finding that the forced dynamic part of the precipitation trends is smaller than the observed full dynamic trend, which is the result of anthropogenic forcing plus internal variability (e.g. line 227-235). The mismatch between the observed full dynamic trend and modelled forced dynamic trend could be due to either 1) the forced trends being underestimated by the models (as the authors claim in the abstract) or 2) large internal variability in the observations. The authors have not shown any evidence that can distinguish between 1) and 2).

Reply: We have revised the title and across our manuscript to avoid strong statement. Our signal-to-noise analysis show that projected multi-model/multi-member mean trends under future increasing emissions closely resemble the present-day pattern in reanalysis (Supplementary Figs. 5a and 21). This similarity suggests that circulation-related signals may emerge earlier than in model simulations, in line with a recent study (Carruthers et al., 2025), and/or because internally driven variability in observations happens to resemble the projected forced dynamic response. Together with your comments, we have changed the title and added detailed explanations in the revised ***Outlook*** (last paragraph) section as follows:

Title: Uncertain dynamic response of mid-latitude winter precipitation to human

emissions

Outlook: The projected decline in dynamic precipitation trends over the SEU, also reported by Carruthers et al., (2025), closely resembles the present-day pattern in ERA5. This correspondence may arise either because internally driven variability in observations happens to resemble the projected forced response, and/or because climate models underestimate the magnitude of the forced dynamic contribution. In either case, improving the representation of large-scale circulation is critical for increasing confidence in regional precipitation projections.

Carruthers, James G., Hayley J. Fowler, Daniel Bannister, and Selma B. Guerreiro. Dynamical adjustment reveals spatial patterns of wetting and drying in European winter precipitation. Environmental Research Letters 20, no. 11 (2025): 114085.

2. For the authors to show that the forced trends are in reality 'strong', and that models underestimate this response, the authors would have to quantify the observed forced dynamic trend. The fundamental problem, however, is that the forced response in observations cannot be determined because it represents a single realization. The observed trends are the result of the forced response plus internal variability, and the study is not able to quantify the relative magnitude of the two components, as also stated in l. 274-275 ('Because ERA5 reanalysis reflects a single realization, ideal separation into internal, forced dynamic and thermodynamic components is not possible'). In short, I did not find any evidence to support the notion that the observed trends are mainly the result of human emissions, as the trends could also have been caused by internal variability.

Reply: Thank you for pointing out overstatements in our previous version. Our intention is not to imply that the forced trends necessarily dominate the observed trends, but rather that a forced signal may be emerging in the real world. Taking precipitation trends over the Mediterranean as an example, the forced circulation-related trends account only for around 9.7% of observed dynamic trends, rendering the forced signal difficult to detect. In addition, the forced dynamic response over the Mediterranean (Fig. 3a,c,e,g) is partly offset by the forced thermodynamic response (Fig. 3b,d,f,h), suggesting the strong sensitivity of precipitation to competing mechanisms in this region. Furthermore, in a setting where multi-decadal internal variability is substantial, the detectability of forced dynamic/thermodynamic signal also depends on the period examined. We provided a more detailed discussion in the revised ***Outlook*** (Fourth paragraph) as follows:

Our results are consistent with theirs: the forced dynamic signal in climate models is

weak relative to internal variability, rendering detection inherently difficult. Whether this signal is genuinely small or systematically underestimated by models cannot by resolved from the observational record alone. As forcing intensifies, the dynamic signal may break the long-term stationarity documented by Vicente-Serrano et al. (2025). Recent evidence that multi-decadal NAO increases in response to greenhouse gases (Smith et al., 2025) supports this interpretation, indicating the potential emergence of a forced circulation signal. The detectability of such a signal depends critically on the period examined. We find significant drying for 1950–2022, while shorter periods show weaker or no trends (Supplementary Fig. 3). Similar circulation-associated drying has been reported for 1921–2015 (Guo et al., 2019) and 1959–2021 (Seager et al., 2024), yet is not reproduced by CMIP6 models. In contrast, Doane-Solomon et al. (2025) show that SEU precipitation trends during 1980–2022 are insignificant, with a weak dynamic component that is well captured by model ensembles. This period-dependence demonstrates strong internal variability rather than directly constraining the forced component's magnitude (Supplementary Fig. 19). Strong multi-decadal NAO oscillations may overwhelm a moderate forced signal (Supplementary Fig. 20), explaining why only certain sufficiently long and recent periods reveal the emerging anthropogenic contribution.

3. Compared to the claims made in the title and abstract, the authors are more cautious and balanced in the main paper stating that '.., climate models likely underestimate the circulation response to human forcings' (l. 237) and 'climate models likely underestimate the magnitude of the externally forced circulation changes in this region.' (l. 263) and 'This suggests that current climate models may underestimate forced dynamic contributions…' (l. 307). But this caution is not reflected in the abstract nor title of the paper, which I therefore find very misleading.

Reply: We have toned down the Title and Abstract as follows:

Title: Uncertain dynamic response of mid-latitude winter precipitation to human emissions

The thermodynamic contribution is broadly reproduced by climate models, whereas the dynamic contribution can exceed model estimates. In circulation-dominated regions such as the Mediterranean, the forced dynamic signal from model simulations explains only 9.7% of the observed dynamic trend, rendering detection challenging. Under continued anthropogenic emissions, projected circulation response intensifies and more closely resembles present-day reanalysis patterns. Although internal variability in observations may contribute to this similarity, the results suggest an uncertain yet potentially emerging role of dynamic response in shaping regional winter precipitation trends.

Other comments:

1) In some instances, it hard to follow the reasoning of the authors due to (to me) non-intuitive notations. For example, I don't understand why the pre-industrial simulations with nudged circulation from the historical/ssp370 simulations are denoted by 'Nudged-Ex3'.

Reply: We originally used 'Ex3' to denote the experiment comprising three paired free-running nudging simulations. To improve clarity and facilitate readability, we have renamed 'Nudged-Ex3' as 'Nudged free-running' and have carefully corrected and standardized all related notations throughout the manuscript.

2) l. 164-165: This statement does not seem to be consistent with Figure 2b, with the CRU-NCEP derived thermodynamic trend being outside the model spread.

Reply: Thank you for pointing out it. Thermodynamic trends derived from the CRU-NCEP dataset may be associated with low confidence because of inconsistencies between the CRU and NCEP products. To further validate our main results, we therefore include the JRA-3Q reanalysis spanning from 1950 as an independent comparison. As expected, the JRA3Q and ERA5-based thermodynamic precipitation trends are better encompassed by climate model ensembles than those derived from CRU-NCEP. Anyway, we have toned down the corresponding statements in the revised ***Precipitation trends decomposition*** Section as follows:

Importantly, thermodynamic trends derived from climate models generally agree with those from reanalysis (Fig. 2), indicating that climate models are able to capture the thermodynamic component of the observed changes in precipitation, in line with previous findings (Guo et al., 2019). This differs from assessments of the mean climate state, which have reported large biases in simulated thermodynamic processes (Wehrli et al., 2018). Overall, our results suggest that despite such biases may affect climatology, thermodynamic precipitation trends are captured by climate models with high confidence.

Guo, R., Deser, C., Terray, L., & Lehner, F. (2019). Human influence on winter precipitation trends (1921–2015) over North America and Eurasia revealed by dynamical adjustment. Geophysical Research Letters, 46(6), 3426-3434.

Wehrli, K., Guillod, B. P., Hauser, M., Leclair, M., & Seneviratne, S. I. (2018). Assessing the Dynamic Versus Thermodynamic Origin of Climate Model Biases. Geophysical Research Letters, 45(16), 8471-8479.

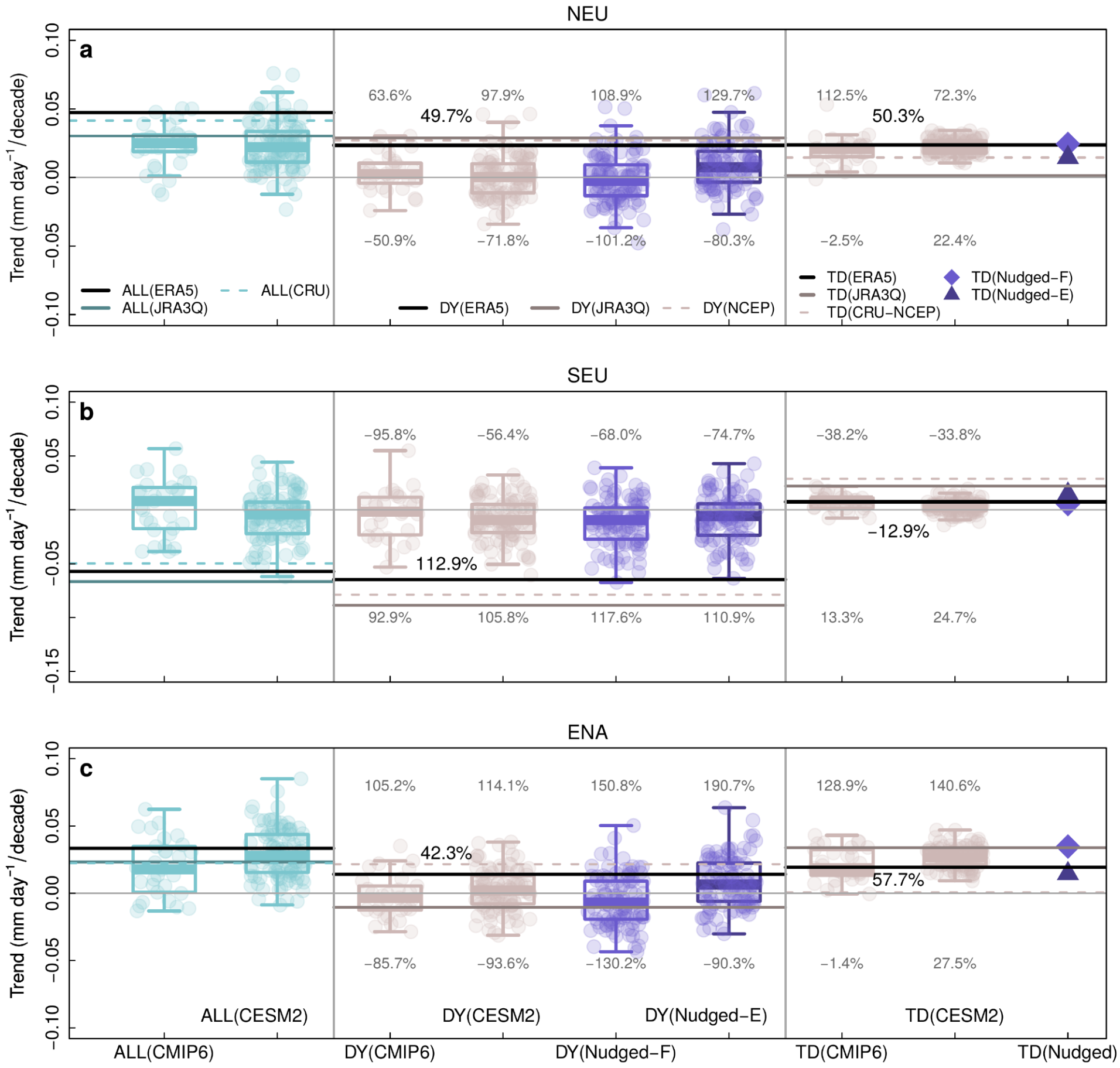

Fig. 2. Total, dynamic, and thermodynamic precipitation trends for winter precipitation. a, Winter precipitation trends (ALL) over the northern Europe (NEU) during 1950–2022 decomposed into dynamic (DY) and thermodynamic (TD) components (mm day^{-1} / decade). 'CMIP6' and 'CESM2', trends based on the elastic-net model from the CMIP6 and CESM2 ensembles. 'Nudged-F' denotes decomposed trends based on the free-nudging climate model experiments (Methods); 'Nudged-E' denotes decomposed trends from the nudged experiments derived with ERA5 winds. Horizontal lines represent trends from the ERA5, CRU, and JRA-3Q datasets. DY(NCEP) is estimated by using sea level pressure from the NCEP reanalysis, TD(CRU-NCEP) is then estimated by subtracting the DY(NCEP) from the ALL(CRU) precipitation trends. **b,c,** The same as **(a),** but for precipitation trends over southern Europe (SEU) and over eastern North America (ENA). Black numbers denote dynamic and thermodynamic contributions relative to total precipitation trends from the ERA5. Gray numbers represent dynamic and thermodynamic contributions from model simulations relative to total precipitation trends using ERA5 as the benchmark.

3) L. 185-186: 'the most likely simulation'. Not clear what is meant with that. After some digging I see that a description is given in the methods section, but that description is unclear as well. Is it the ensemble member with the highest spatial or temporal correlation? Why is that selected, and how should it be interpreted?

Reply: We have rephrased 'the most likely simulation' as 'the best-matching simulation', which is identified as the member exhibiting the highest spatial correlation (above 30°N) between simulated and ERA5-derived total and dynamic precipitation trends. Notably, the trend metric itself implicitly accounts for the temporal coherence of the underlying time series across grid points. Actually, the best matching simulations

do not necessarily coincide with those that best reproduce the thermodynamic precipitation trends, nor with those that most accurately capture the large-scale circulation background (e.g., *SLP* background). In other words, the best matching realizations reproduce the total and dynamic precipitation trends reasonably well, despite inconsistencies in the thermodynamic and circulation backgrounds. We have discussed this point in the revised ***Physical drivers of decomposed precipitation trends*** section as follows:

However, the 'best-matching simulation' (Methods) captures little of the observed shift in atmospheric dynamics over recent decades (Supplementary Fig. 5). The pattern correlations (above 30°N) of *SLP* (*U500*) between CMIP6 and CESM2-LE simulations and ERA5 are 0.05±0.31 and 0.11±0.34 (0.00±0.20 and –0.01±0.27), respectively (Supplementary Fig. 7). Accordingly, the simulation that best reproduces dynamic precipitation trends does not correspond to the highest correlations in either *SLP* or *U500*. This indicates limitations of climate models to reproduce observed dynamic precipitation trends in Europe.

Despite modest spatial heterogeneity, the ability of climate models to reproduce trends in thermodynamic indicators (for example, R=0.33±0.13 between CMIP6 and ERA5 evapotranspiration patterns and R=0.40±0.06 between CESM2 and ERA5 patterns, Supplementary Fig. 9) supports their ability to capture changes in observed thermodynamic precipitation.

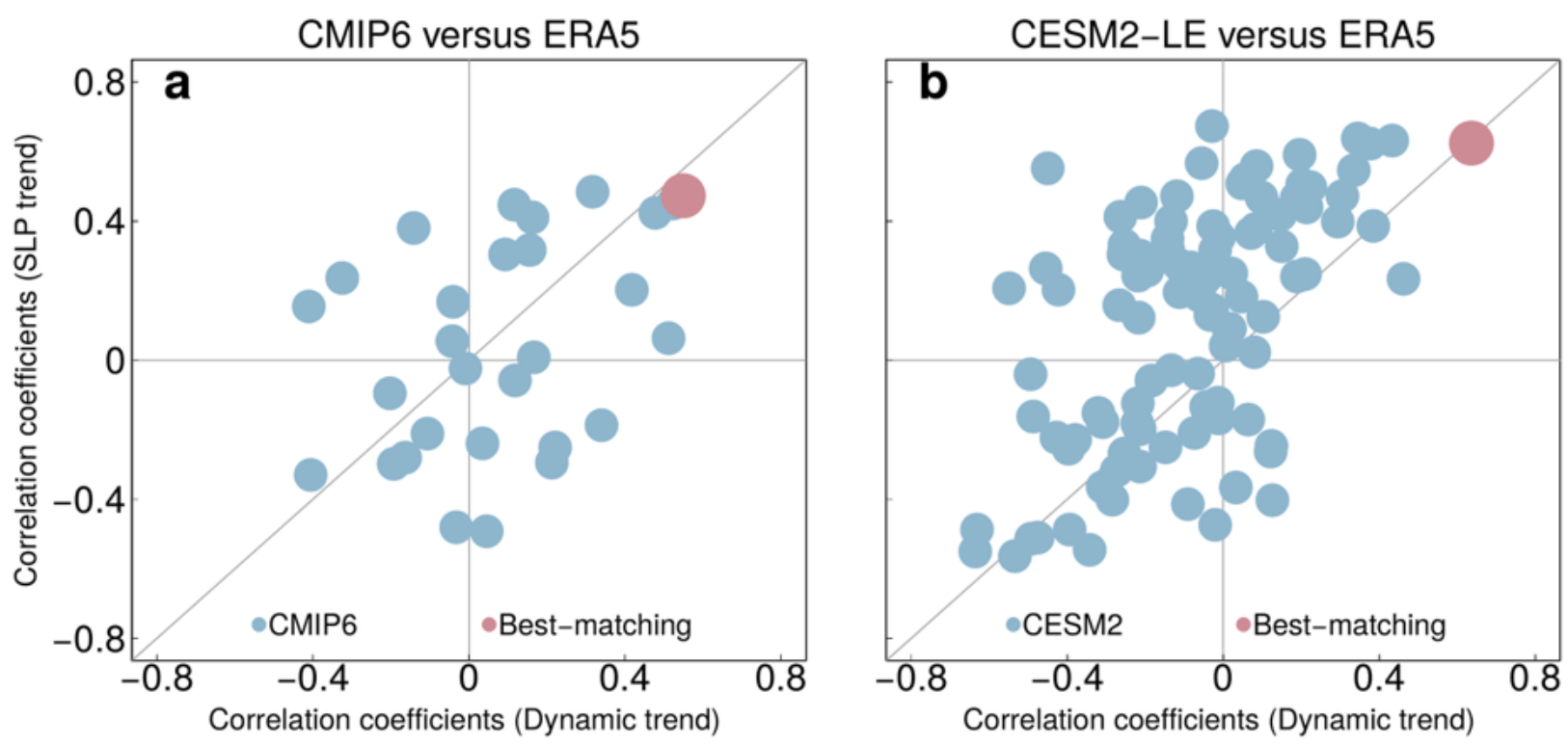

Supplementary Figure 7. The Pearson correlation of dynamic precipitation trend and dynamic indicator trend between climate model simulations and the ERA5 reanalysis. a, The correlation coefficients between the CMIP6 ensemble simulations and the ERA5 reanalysis. *x*-axis denotes the correlation of dynamic precipitation trend; *y*-axis denotes the correlation of sea level pressure (SLP) trend. **b**, The same as **a**, but correlation between the CESM2-LE and the ERA5.

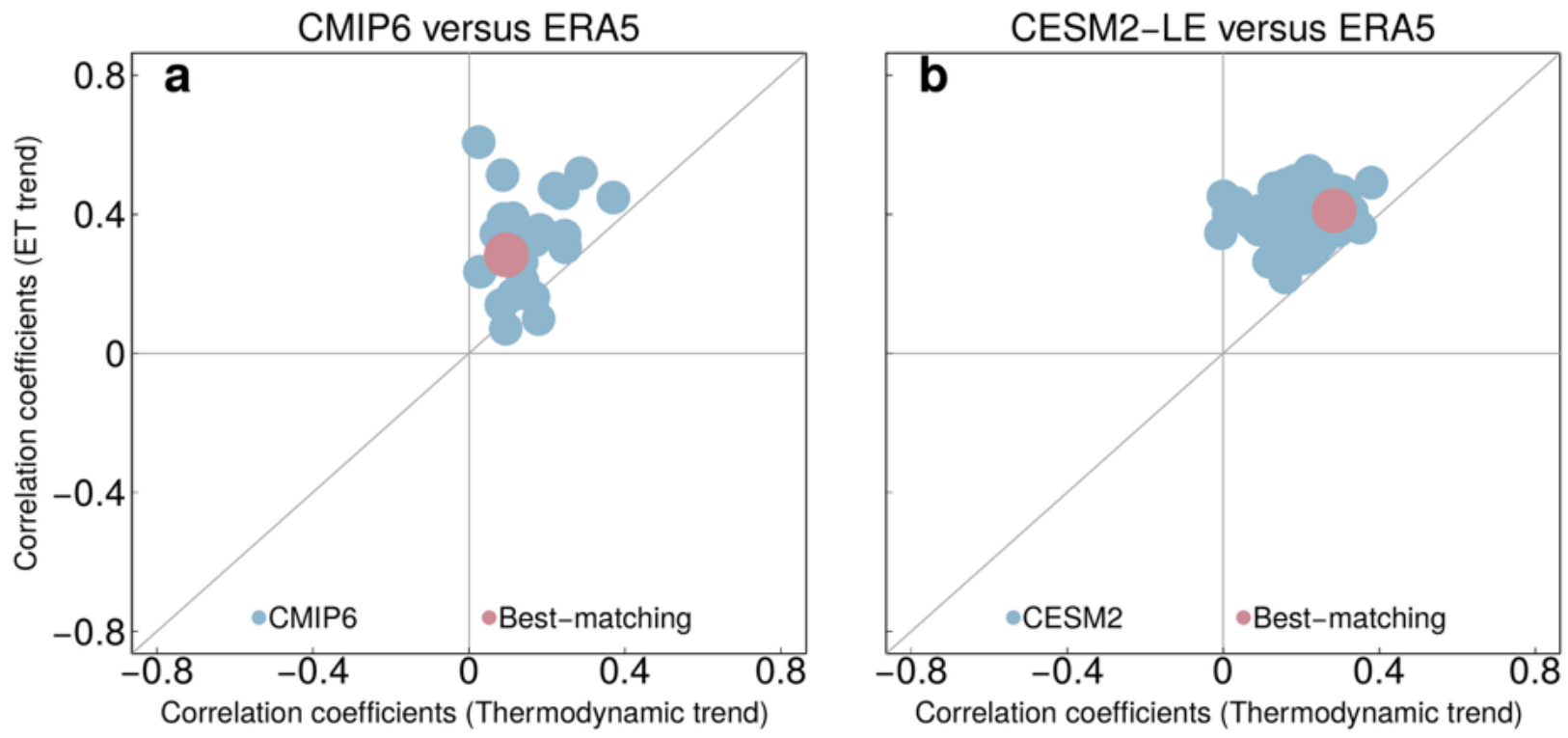

Supplementary Figure 9. The Pearson correlation of thermodynamic precipitation trend and thermodynamic indicator trend between climate model simulations and the ERA5 reanalysis. **a**, The correlation coefficients between the CMIP6 ensemble simulations and the ERA5 reanalysis. *x*-axis denotes the correlation of thermodynamic precipitation trend; *y*-axis denotes the correlation of evapotranspiration (ET) trend. **b**, The same as **a**, but correlation between the CESM2-LE and the ERA5.

4) L. 217: Not clear to me what is exactly meant with 'the multi-model mean signal'

Reply: Sorry for this vague statement. We have revised the phrase as "the multi-model mean trends".

5) L. 225: 'These result imply that thermodynamic regulation of precipitation is largely driven by anthropogenic forcing'. This is too strong a statement. I would change this to something like 'these results suggest that in the observed record, changes in thermodynamic regulators of precipitation are mainly driven by anthropogenic forcing, with internal variability playing a secondary role'

Reply: Insightful comment! We have revised the statement as follows:

These results suggest that, in the observational record, changes in thermodynamic precipitation are mainly driven by anthropogenic forcing, with internal variability playing a secondary role.

6) L. 251: 'access' or 'assess'?.

Reply: Thank you for spotting this typo. It should be 'assess'. The revised manuscript has been double-checked.

Reply to Reviewers' comments

Legend
Reviewers' comments
Authors' responses
Direct quotes from the revised manuscript

Reviewer #1 Remarks to the Author:
This is the revised version of the manuscript that I have previously reviewed. I would like to thank the authors for their efforts in revising the manuscript and for their point-by-point responses to my comments. The authors have performed a significant amount of additional work, especially for the comparison of thermodynamic and dynamic components of precipitation derived from traditional moisture budget-based estimation and their definition. My concerns regarding the technical details for model experiments design are also clarified. The revisions have addressed nearly all my initial concerns. I recommend accepting it for publication in Nature after some further minor and technical revisions.

Reply: We thank the reviewer for the supportive feedback. As suggested, we have added a figure illustrating the evolution of winter precipitation over NEU, SEU, and ENA from CMIP6 and CESM2-LE ensembles. We have also included a table documenting the estimation of thermodynamic and dynamic components within the CESM2 experimental framework. We hope that these revisions have improved the clarity of the manuscript and the revised version is now suitable for publication.

Specific comments

1. In response to my major comment#2, the authors explained that "the Forced DY (Nudged-ERA5) is less reliable: it is derived by subtracting forced TD (Nudged-ERA5) from CESM2-LE ensemble mean, but the ERA5-nudging uses a prescribed ocean while CESM2-LE is fully interactive. This might explain why the improved constraint from ERA5 winds does not translate into a cleaner forced DY estimate". These distinctions are discussed in the revised "Attribution to external forcing" section as "Differences between the two configurations partly reflect contrasting ocean boundary conditions: the free-running setup uses a fully coupled ocean, whereas the ERA5-nudged configuration prescribes oceans." The weakness of experimental design can be acknowledged in the "Constrained climate model with ERA5 winds (experiment 2)" part of Method section.

Reply: We thank the reviewer for this insightful suggestion. We have acknowledged the weakness of experimental design in the revised ***Method*** section as follows:

As in experiment 1, the difference between forced and preindustrial simulations is interpreted as the anthropogenic forced thermodynamic component. The forced dynamic precipitation component is then estimated by subtracting this forced thermodynamic component from the CESM2-LE ensemble mean. Note that the ERA5-nudging experiment uses prescribed sea surface conditions, whereas CESM2-LE is fully coupled. Consequently, the imposed constraint from ERA5 winds does not necessarily yield a cleaner estimate of the forced dynamic component, as atmosphere-ocean coupling processes that interact with circulation and precipitation are not consistently represented across the two frameworks.

2. Fig. 1. Observational and simulated trends in winter precipitation 1950–2022. Please show the results of Fig.1e-g derived from the CMIP6 MME and CESM2-LE MME as supplementary figure. This helps the readers to quantitatively understand the weaknesses of climate models.

Reply: Thank you very much for this insightful comment. We have included the line plot of CMIP6 MME and CESM2-LE MME in the revised Extended Data Fig. 2 as follows:

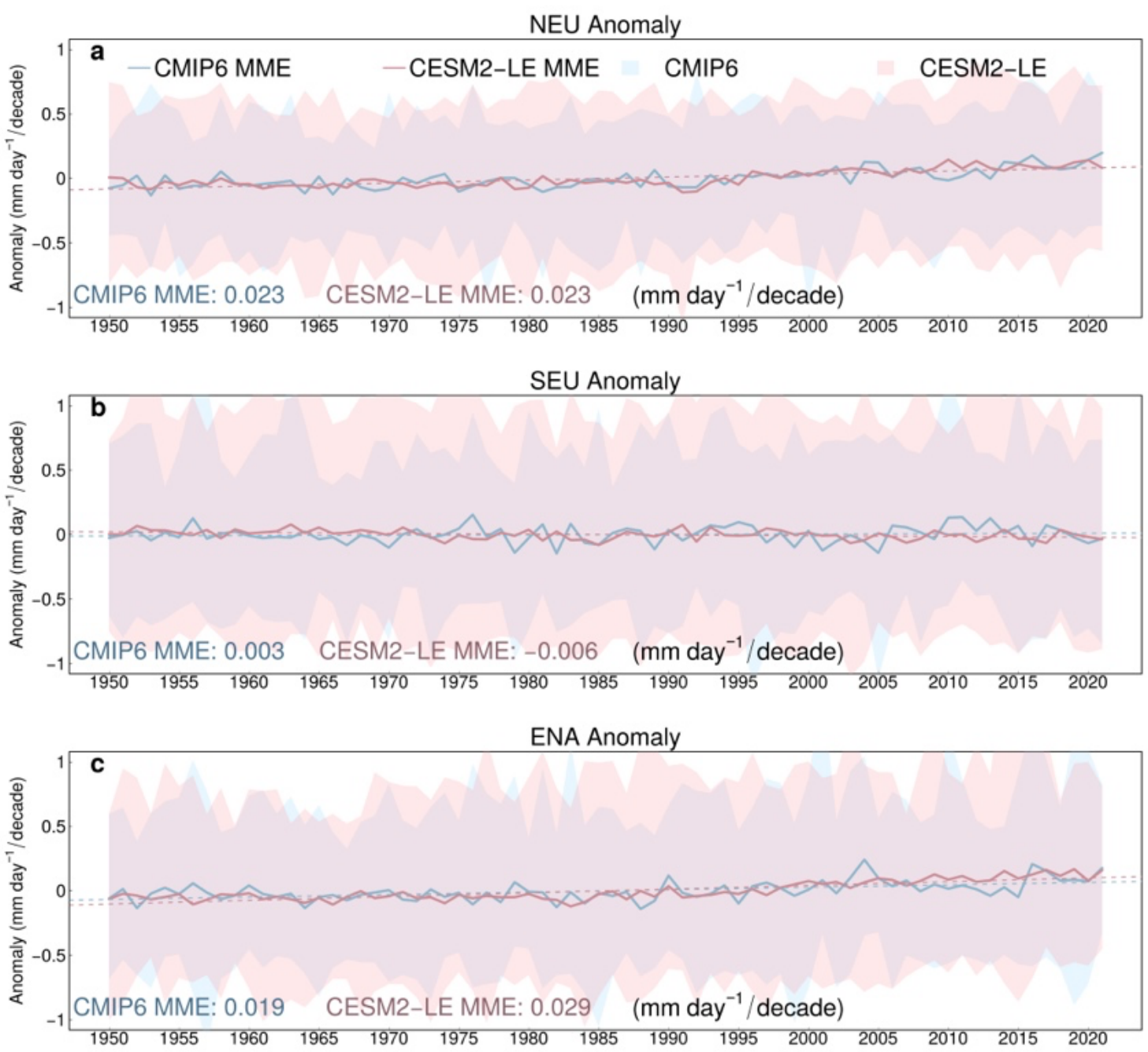

Extended Data Fig. 2 | Simulated winter precipitation trends (1950–2022). a, Evolution and trends of winter precipitation over NEU from CMIP6 and CESM2-LE ensembles. Lines denote ensemble means (multi-model mean for CMIP6; multi-member mean for CESM2-LE), and shading denotes the spread (minimum to maximum) of precipitation evolution from the CESM2-LE and CMIP6 ensembles. **b-c,** As in **a,** but for SEU **(b)** and ENA **(c).**

3. L139-148: "In parallel, we run nudged CESM2 experiments that take dynamic

conditions from free running historical and future (SSP3.7-0) simulations with anthropogenic greenhouse gas and aerosol emissions as well as land use changes, and prescribe these dynamic conditions to control simulations without external forcing (Nudged-F). To assess the influence of observed circulation patterns, we additionally run CESM2 nudged experiments using horizontal winds from ERA5 (Nudged-E). Subtracting the unforced nudged simulations from the externally forced ones yields idealized thermodynamic precipitation trends (Fig. 2,TD(Nudged)). Further subtracting this forced thermodynamic component from the 100-member CESM2 ensemble produces full dynamic precipitation components (Fig. 2, DY(Nudged)) that are independent of the statistical learning approach". It is a bit complex for the readers to understand this statement, especially for those who are not expertized in climate modeling. To help the readers easily understand the modeling strategy, you may add one Table in the Method section and thus clarify how the thermodynamic and dynamic components are estimated/derived.

Reply: Good suggestion. We have added the following Table in the ***Method*** section as follows:

Extended Data Table 1 | Experimental design and component decomposition of precipitation

Experiment	Simulation	Description	Thermodynamic component	Dynamic component
**Exp. 1**	Exp. 1a Fully-coupled transient CESM2 run (Run #1 – Run #3)	Fully-coupled simulations with historical and SSP3-7.0 forcing	TD(Nudged-F) = Exp. 1a – Exp. 1b	DY(Nudged-F) = Exp. 1c – TD(Nudged-F)
	Exp. 1b Nudged CESM runs (Run #1 – Run #3)	Simulations with prescribed horizontal winds from fully-coupled transient run but with fixed pre-industrial forcing		
	Exp. 1c CESM2 large ensemble mean (open-source data)	Ensemble mean of CESM2 simulations with historical and SSP3-7.0 forcing		
**Exp. 2**	Exp. 2a Historical CESM2 AMIP run nudged with ERA5 horizontal winds	Simulation with prescribed observed SST nudged with ERA5 winds and forced	TD(Nudged-E) = Exp. 2a – Exp. 2b	DY(Nudged-E) = Exp. 1c – TD(Nudged-E)

		with transient historical forcing		
	Exp. 2b Pre-industrial CESM2 AMIP run nudged with ERA5 horizontal winds	Pre-industrial simulation with prescribed SST (detrended observed SST) nudged with ERA5 winds and forced with fixed pre-industrial forcing		

4. L202-204: "Accordingly, the simulation that best reproduces dynamic precipitation trends does not correspond to the highest correlations in either SLP or U500. This indicates limitations of climate models to reproduce observed dynamic precipitation trends in Europe." One interesting question here is: where do the best-matching dynamic precipitation trends come from? Any explanation?

Reply: In-depth question. This mismatch likely arises from regional heterogeneity in the relationship between SLP/U500 and dynamic precipitation trends. We briefly discussed it in the revised ***Methods*** section as follows:

Note that the simulation that best reproduces dynamic precipitation trends does not exhibit the highest correlations in SLP or U500. This likely reflects regional heterogeneity in the relationship between circulation and precipitation. Large-scale circulation patterns (SLP and U500) exert spatially varying controls on precipitation, being more influential in some regions than others. Meanwhile, the fidelity of simulated circulation trends relative to reanalysis also varies regionally. Consequently, regions where circulation both strongly influences precipitation and is well reproduced by models do not necessarily align with those that dominate hemispheric-average metrics.

5. L457-459: "Specifically, this experiment is conducted in an atmosphere-only configuration (AMIP style) with prescribed ocean boundary conditions." AMIP type simulation means that the AGCM was driven by historical SST with year-by-year change. I understand that your sea surface temperature is derived from the pre-industrial run and thus does not have observed year-by-year variability. Is this understanding correct?

Reply: For the "historical and SSP370" simulation, the AGCM uses observed SST from the Met Office Hadley Centre, which includes observed year-to-year variability. For the "pre-industrial" simulation, the forced component is removed from the observed SST using a low-frequency filtering method (Wills et al., 2020). The interannual variability is largely preserved, but may differ in amplitude from the observed SST due to the

removal of low-frequency variability. This has been included in the revised ***Method*** section as follows:

This approach preserves the year-to-year variability while altering the low-frequency background state, following Ref 60.

[60] Wills, R.C., Battisti, D.S., Armour, K.C., Schneider, T. and Deser, C., 2020. Pattern recognition methods to separate forced responses from internal variability in climate model ensembles and observations. Journal of Climate, 33(20), pp.8693-8719.

6. L463-465: "We find that precipitation trends from the CESM2 forced simulation closely match those from ERA5, confirming the robustness of externally applied forcing in climate models (Supplementary Fig. 13)." In climate modelling community, we generally used the term "forced simulation" to describe the coupled model historical simulations driven by anthropogenic and natural external forcing factors. To avoid misunderstanding, I would suggest you replace "the CESM2 forced simulation" with "the nudged CESM2 AGCM simulation".

Reply: Thank you for this correction! We have replaced "the CESM2 forced simulation" with "the nudged CESM2 AGCM simulation" in the revised manuscript.

Reviewer #2 Remarks to the Author:

The authors have made substantial revisions in response to the other reviewers' and my comments. The revised title and abstract are more balanced and accurately reflect the study's findings. The addition of JRA-3Q data, moisture budget diagnostics, and omega500 binning following Emori and Brown (2005) address my previous concerns about the interpretability and robustness of the dynamic/thermodynamic decomposition. I have one minor comment: Line 91-92: Climate sensitivity refers to the temperature response to a doubling of CO2. Increased regional precipitation variability does not directly imply elevated climate sensitivity in the standard definition of climate sensitivity. Consider removing the reference to climate sensitivity.

Reply: Dear Dr. Miyawaki, we appreciate your positive evaluation. As suggested, we have removed 'climate sensitivity' and rephrased the sentence in the manuscript as follows:

The upward trends in ENA and NEU are accompanied by increasing precipitation variability[3], indicating an intensified hydrological cycle in these regions.

Reviewer #3 Evaluations:

The authors have toned down the overly strong statements in the original submission, addressing my main concerns. I don't have any further comments, except that the following sentence should be improved: (line 169-170): 'Overall, our results suggest that despite such biases may affect climatology, thermodynamic precipitation trends are captured by climate models with high confidence', as it does not read well.

Reply: We appreciate the reviewer's positive feedback; we have revised the sentence as follows:

This suggests that climate models capture the forced thermodynamic response more faithfully than the underlying climatology.
